# Molecular characteristics of *S-RNase* alleles as the determinant of self-incompatibility in the style of *Fragaria viridis*

**DOI:** 10.1038/s41438-021-00623-x

**Published:** 2021-08-01

**Authors:** Jianke Du, Chunfeng Ge, Tingting Li, Sanhong Wang, Zhihong Gao, Hidenori Sassa, Yushan Qiao

**Affiliations:** 1 Laboratory of Fruit Crop Biotechnology, College of Horticulture, Nanjing Agricultural University, Nanjing, 210095 Jiangsu China; 2 Laboratory of Genetics and Plant Breeding, Graduate School of Horticulture, Chiba University, Matsudo, 271-8510 Chiba Japan; 3 Present Address: Institute of Botany, Jiangsu Province and Chinese Academy of Sciences, Nanjing, 210014 Jiangsu China

**Keywords:** Self incompatability, Plant breeding

## Abstract

Strawberry (*Fragaria* spp.) is a member of the Rosoideae subfamily in the family Rosaceae. The self-incompatibility (SI) of some diploid species is a key agronomic trait that acts as a basic pollination barrier; however, the genetic mechanism underlying SI control in strawberry remains unclear. Two candidate *S-RNase*s (*S*_*a*_- and *S*_*b*_-RNase) identified in the transcriptome of the styles of the self-incompatible *Fragaria viridis* 42 were confirmed to be SI determinants at the S locus following genotype identification and intraspecific hybridization using selfing progenies. Whole-genome collinearity and RNase T2 family analysis revealed that only an *S* locus exists in *Fragaria*; however, none of the compatible species contained *S-RNase*. Although the results of interspecific hybridization experiments showed that *F. viridis* (SI) styles could accept pollen from *F. mandshurica* (self-compatible), the reciprocal cross was incompatible. *S*_*a*_ and *S*_*b*_-RNase contain large introns, and their noncoding sequences (promotors and introns) can be transcribed into long noncoding RNAs (lncRNAs). Overall, the genus *Fragaria* exhibits *S*-RNase-based gametophytic SI, and *S-RNase* loss occurs at the *S* locus of compatible germplasms. In addition, a type of SI-independent unilateral incompatibility exists between compatible and incompatible *Fragaria* species. Furthermore, the large introns and neighboring lncRNAs in *S-RNase* in *Fragaria* could offer clues about *S-RNase* expression strategies.

## Introduction

The germplasm of low ploidy wild strawberry contains abundant genetic resources that control valuable traits and is a potential resource for improving cultivated strawberry. However, pollination obstacles between styles and pollen have considerably hampered strawberry breeding^1–6^. Incompatibility includes self-incompatibility (SI) and interspecific incompatibility, and it has been demonstrated that both have common genetic factors and share some intermediate mechanisms in Solanaceae^7,8^. SI is a type of pollination barrier that serves as a basis for studying other incompatibility mechanisms^7,8^. Therefore, the elucidation of the molecular mechanism underlying SI will enable the development of strategies aimed at improving molecular breeding in strawberry.

Gametophytic SI (GSI) is a genetic mechanism that inhibits the self-pollination and growth of inbred pollen tubes. The phenotype is determined by the S haplotype of the pollen, which results in the generation of same-genotype pollen tubes that cannot normally extend to the ovary in the style^9^; this process may involve multiple genetic mechanisms. To date, two GSI mechanisms have been identified, including *S*-RNase-based GSI, identified in Solanaceae, Plantaginaceae, and Rosaceae, and signal transduction GSI, identified in Papaveraceae^7,10,11^. Strawberries belong to the genus *Fragaria* in the subfamily Rosoideae (family Rosaceae). Although there is broad consensus that the family Rosaceae is divided into three subfamilies (Dryadoideae, Rosoideae, and Amygdaloideae), the genetic relationships among the subfamilies remain controversial^12–14^. The tribes Maleae (which includes *Malus* and *Pyrus*) and Amygdaleae (which includes *Prunus*) in the subfamily Amygdaloideae exhibit *S*-RNase-based GSI, and its mechanism has been studied extensively. Among them, the genera *Malus* and *Pyrus* have similar GSI mechanisms that are distinct from the mechanism in the genus *Prunus*^15^. However, little is known about the SI mechanism in the genus *Fragaria* in the subfamily Rosoideae^6,16–19^.

Although Rosaceae members exhibit the *S*-RNase-based GSI system, some self-compatible variants exist in nature^16,17^. Recently, increased research on SI has helped unravel the mechanisms by which some mutations exert their effects. The major reasons for the occurrence of self-compatibility (SC) include the sequence loss of key genes^20,21^ or the loss of function of S-encoded proteins because of sequence variations at the *S* locus^22,23^. SI requires a certain *S*-RNase threshold, and the abnormal expression of *S*-RNase can overcome the SI in a species^24,25^. There are some indications that the *S* intron sequence and *S* locus methylation level can influence the expression of *S-RNase*^26,27^. Flowering plants of the same species, including strawberry, can exhibit both SI and SC. Unilateral incompatibility (UI) often exists between the two types and is considered to be related to SI^28^. In addition, the UI observed in SC × SC crosses supports the existence of additional interspecific barriers that are not dependent on SI^7,8^. The reasons underlying the compatibility of strawberries have not been elucidated, and to our knowledge, there have been no studies on whether there is a types of UI between SI and SC species in *Fragaria* that is not associated with *S*-RNase-based SI.

*Fragaria* spp. exhibit SI, with *Fragaria viridis*, *F. nubicola*, *F. pentaphylla*, and *F. nipponica* exhibiting SI and *F. vesca* and *F. nilgerrensis* exhibiting SC^1,6,29–31^. Bosković et al.^6^ suggested that there are two unlinked RNase loci that control strawberry incompatibility that are different from the single *S* locus in Amygdaloideae^17^. The presence of any locus can result in the specific rejection of cognate pollen, in addition to the nonspecific rejection of pollen with *Sn* and *Tn* null allele genotypes. The polypeptides encoded by the two loci are also different from those in the Amygdaloideae subfamily. The above results are based on analyses of style RNase; however, not all RNases expressed in styles are *S-RNase*^6,18^. Therefore, whether there are two S control loci still requires further verification. Similarly, *S*-RNases that determine the style type in incompatible species of the genus *Fragaria* need to be explored further.

It is speculated that the genus *Fragaria* exhibits *S*-RNase-based SI similar to that in the Maleae and Amygdaleae tribes^6,18^. In the present study, two *S-RNase* candidates (*S*_*a*_, *S*_*b*_-RNase) were obtained from the style expression database of *F. viridis* 42. The sequence and expression analysis of the two candidates, the identification of the genotypes of selfing lines, and intraspecific hybridization between different S genotype lines confirmed that *S*_*a*_ and *S*_*b*_-RNase are alleles and style SI determinants in *F. viridis*. Expression analysis of *S-RNase* and the whole-genome identification of the RNase T2 family genes showed that *S-RNase* was lost in self-compatible strawberry germplasms. Only one *S* locus was found on chromosome 6 based on collinearity analysis using the *F. vesca* genome and RNase T2 family genes analysis in *F. viridis*. Interspecific hybridization revealed a principle inconsistent with the SI × SC rule in *Fragaria* plants, in addition to having no correlation with the *S*-RNase genotype. Furthermore, UI was observed between compatible strawberries. In addition, the *S-RNase* of the genus *Fragaria* is a very large gene (*S*_*a*_ and *S*_*b*_-RNase are 30 and 23 kb, respectively), and the noncoding sequence of the genes (intron and promotor) can transcribe long noncoding RNAs (lncRNAs); these may provide new insights into the mechanism of the regulation of *S*-RNase expression.

## Results

### Screening and analysis of *S*-RNase candidate genes

#### Screening of RNase T2 family genes in *F. viridis* and *F. vesca*

*S*-RNase belongs to Class III in the RNase T2 protein family^32^. In contrast to non-*S*-RNase genes, *S*-RNase, as a candidate SI determinant, has specific characteristics that distinguish it from other RNase genes^11,15,18^. RNase T2 family gene members were screened using HMM, and 14 and 13 genes were screened from the proteome database in *F. vesca* and *F. viridis*, respectively. Detailed information about the characteristics of the selected RNase T2 family genes is shown in Table 1. Based on this information, SI determinant candidates in *F. viridis* were selected, and SC analysis in *F. vesca* was performed. Flower balls containing styles from wild *F. viridis* 42 at 0 h and 24 h after self-pollination were selected for transcriptome sequencing and for further use in the establishment of a style proteome database. The basic data used to obtain the proteome data for *F. viridis* have been submitted to the Sequence Read Archive database. The accession numbers are PRJNA361176, PRJNA361185, PRJNA361192, PRJNA361199, PRJNA361204, and PRJNA361208.

**Table 1:** Characteristics of the RNase T2 gene family in *F. vesca* and *F. viridis*

Gene ID	Chromosome location	Intron number	pI	Molecular weight	Signal peptide	Domain size	Number of amino acids	Pattern 4
FvH4_4g31300.1	Chr4:30502482	7	5.38	102.0	N	>60%	916	CPSSNG
FvH4_4g31290.1	Chr4:30499449	3	5.23	25.7	Y23	>60%	229	CPSSNG
FvH4_2g17310.1	Chr2:14946893	7	8.17	27.6	Y28	>60%	245	—
FvH4_5g24800.1	Chr5:16134164	3	9.37	26.6	Y26	>60%	239	—
FvH4_1g10040.1	Chr1:5448902	1	8.08	26.0	Y19	>60%	230	—
FvH4_6g07740.1	Chr6:4649328	1	6.21	25.2	Y21	>60%	220	—
FvH4_6g07690.1	Chr6:4634036	1	6.28	26.2	Y15	>60%	226	—
FvH4_5g33850.1	Chr5:24565949	1	8.07	31.3	Y43	>60%	270	—
FvH4_1g19170.1	Chr1:11366424	1	8.95	22.7	Y23	>60%	198	—
FvH4_4g18130.1	Chr4:22047584	1	8.09	27.7	Y19	>60%	241	—
FvH4_2g25650.1	Chr2: 20739797	3	9.39	25.6	N	<60%	223	—
FvH4_6g22290.1	Chr6: 15948673	1	7.66	27.4	Y17	<60%	238	—
FvH4_2g25620.1	Chr2: 20733334	2	9.81	15.5	Y20	<60%	133	—
FvH4_5g24550.1	Chr5: 15908338	2	8.55	12.3	Y26	<60%	109	—
Unigene18150.1	Chr4:30504261	2	4.58	25.2	Y27	>60%	231	CPSSSG
Unigene13465.1	Chr4:30500729	3	5.35	25.8	Y23	>60%	229	CPSSNG
Unigene11523.1	Chr2:14950589	6	5.76	30.5	Y29	>60%	278	—
Unigene23139.1	Chr6:5067585	1	8.53	26.2	Y26	>60%	228	—
CL6424.Contig1.1	Chr1:5449834	1	8.57	25.8	Y19	>60%	229	—
Unigene10929.1	nr.	2	8.68	25.2	Y23	>60%	218	—
Unigene7320.1	nr.	2	8.55	25.6	Y24	>60%	221	—
Unigene9248.2	Chr5:24566583	0	5.47	12.4	N	<60%	104	—
Unigene638.1	Chr6:4649354	1	5.26	12.4	Y19	<60%	109	—
Unigene31475.1	Chr6:4634931	1	6.88	8.8	N	<60%	79	—
Unigene23536.2	Chr1:4771772	0	6.52	5.6	N	<60%	50	—
Unigene27236.1	Chr2:14949271	0	5.86	9.1	N	<60%	79	—
CL6424.Contig2.2	Chr1:5449478	1	8.94	8.5	N	<60%	75	—

The genes with a “FvH4” prefix are from *F. vesca*, and those with “CL” or “Unigene” prefixes are from *F. viridis*. The “.1” suffix represents the first transcript of the gene in *F. vesca*, and the “.1” and “.2” suffixes represent the first and second open reading frames (ORFs) of the gene, respectively, in *F. viridis*. All identified genes of the RNase T2 family from *F. vesca* were consistent with the results of a previous screening^43^. The gene position and intron number for *F. vesca* were obtained according to the GFF file (*F. vesca* genome_v4.0.a1). The position information for *F. viridis* was determined mainly based on the *F. vesca* genome_v4.0.a1 reference and was located using BLASTn. Intron number and position analyses were based on the characteristic structure of the intron boundary (the intron analysis of only Unigene23139.1 refers to the *F. nilgerrensis* genome, v1.0). “nr.” means that the location information could not be obtained. In the signal peptide column, “N” indicates a lack of signal peptide information, “Y” indicates the presence of signal peptide information, and the number indicates the size of the signal peptide. The domain size is represented as a percentage, which is the percentage of the RNase T2 domain contained in the selected protein within the full-length domain. Pattern 4 (RNase T2 lineage amino acid pattern 4) is a type of amino acid sequence; if an RNase T2 family member contains the pattern 4, it is not an *S*-RNase or *S*-lineage gene^18^. The domain analysis of the RNase T2 family genes in *F. vesca and F. viridis* (see Supplementary Fig. S1) and chromosomal localization information are presented in Supplementary Figs. S2 and S3, respectively. The size of the gene and the gene structure are shown in Supplementary Fig. S4. The intron position and number for Unigene10929.1 and Unigene7320.1 were analyzed, as shown in Supplementary Figs. S5, S6, and Supplementary Dataset S1.

#### *S*-RNase prediction in *F. viridis* and *F. vesca*

The presence of a signal peptide is one of the prerequisites for the extracellular secretion of proteins^22,33,34^, and 12 and 8 deduced proteins containing signal peptides were obtained from *F. vesca* and *F. viridis*, respectively. The functional domain of RNase T2 is a basic structure that contains essential functional units and plays a key role in mediating GSI responses^35–38^. We set a domain integrity >60% as the condition for screening *S*-RNase and further filtered 3 and 1 nonstandard proteins in *F. vesca* and *F. viridis*, respectively. The isoelectric point (pI) of *S*-RNase has been reported to range from 8 to 10^18,32,39,40^. Based on the pI, three genes were simultaneously eliminated from *F. vesca* and *F. viridis* based on the last filtered result. As the number of *S-RNase* introns does not exceed two^39,41^, four proteins were obtained from *F. vesca* (FvH4_1g10040.1, FvH4_5g33850.1, FvH4_1g19170.1, and FvH4_4g18130.1), and four were obtained from *F. viridis* (Unigene 23139.1, CL6424.Contig1.1, Unigene 10929.1, and Unigene7320.1). *S*-RNase exhibits a high degree of polymorphism, with amino acid identity ranging from 30 to 90%^11,16,37,42^. Further identity analysis of the four selected proteins in *F. viridis* revealed two combinations that satisfied the conditions. The amino acid similarity between Unigene10929.1 and Unigene7320.1 was 56.48%, and that between Unigene23139.1 and Unigene7320.1 was 30.88% (Supplementary Table S1). The similarity between *S*-RNases within the same genus is known to be higher than that between *S*-RNases of different tribes^35,37^; this applied only for Unigene10929.1 and Unigene7320.1 in this study (Supplementary Table S1). The amino acid similarity of the four obtained *F. vesca* proteins with Unigene10929.1 and Unigene7320.1 was very low (<30%). Unigene10929.1 and Unigene7320.1 were confirmed as the RNase T2s linked to the SI phenotype (see the section “Intraspecific hybridization between different S genotype lines of *F. viridis*”); by extension, there were no eligible *S-RNases* in *F. vesca*. In addition, unlike the RNase T2 family genes that were assessed to be non-*S*-RNases, the position on the chromosome, the number of amino acids, and the amino acid sequence pattern indicated that Unigene10929.1 and Unigene7320.1 are candidate *S-RNase*s^6,16,39,41^.

#### Evolutionary analysis of RNase T2 family members

**Fig. 1:**
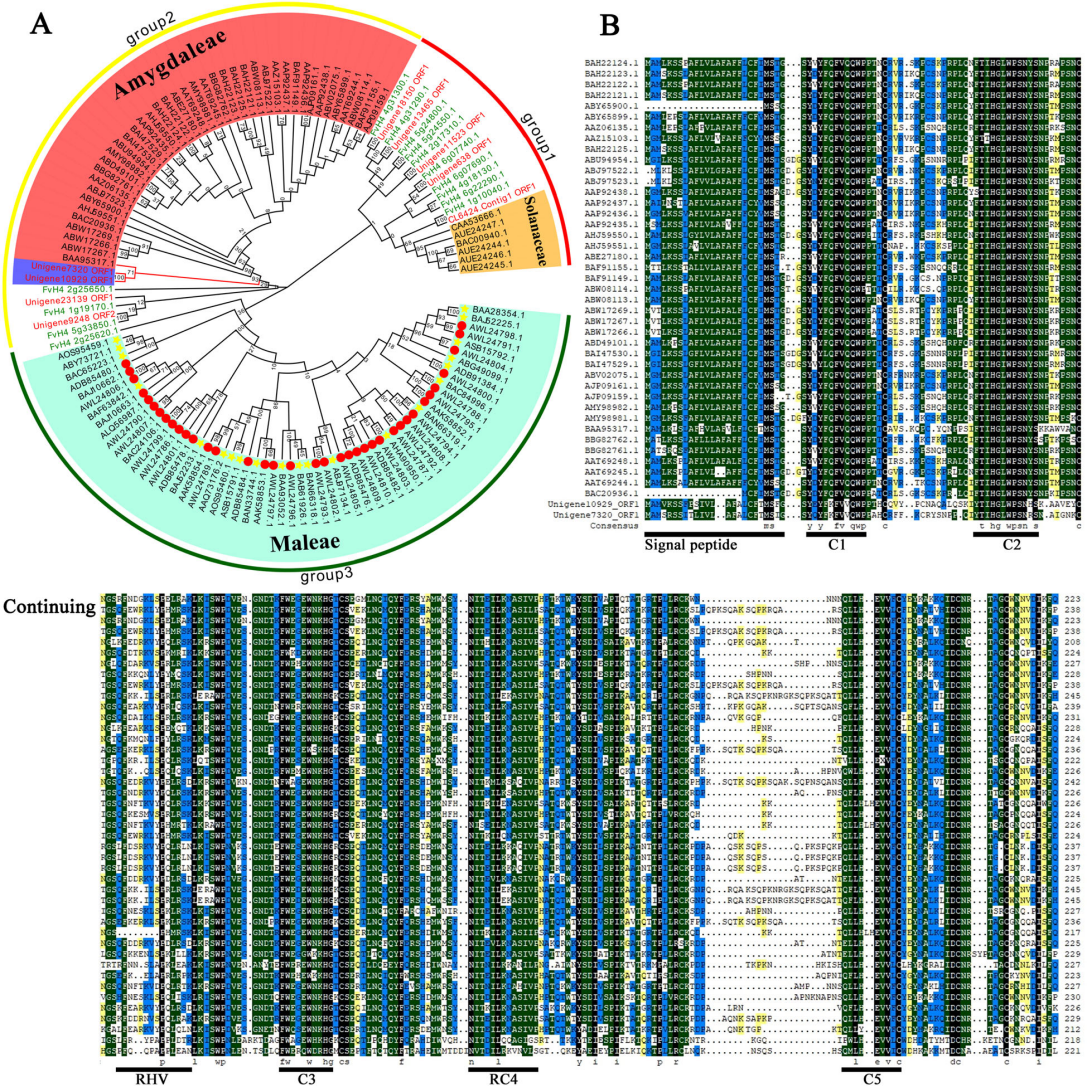
The phylogenetic tree of the RNase T2 gene family and the conserved gene sequence of candidate *S-RNase*s in the genus *Fragaria*. **A** The *S-RNase* genes with red, orange, and light blue backgrounds are from the tribe Amygdaleae, the family Solanaceae, and the tribe Maleae, respectively. The red circles represent *Malus*, and the yellow five-pointed stars represent *Pyrus*. The genes in red and green fonts are from *F. viridis* and *F. vesca*, respectively. Groups 1, 2, and 3 represent the three groups clustered together, which are marked with red, yellow, and green circular bands, respectively. Bootstrapping was performed with 1000 replicates. **B** *S*_*a*_ and *S*_*b*_-RNase are closely related to the genus *Prunus*; therefore, 41 *Prunus S-RNase*s (Supplementary Table S2) were used to compare and analyze the conserved *S-RNase* structure in *F. viridis*. Based on the *S*-RNase characteristics of Rosaceae^35,37,38^, the conserved *S-RNase* structure in *F. viridis* was divided further into C1–C3, RC4, and C5 as conserved regions and RHV as a hypervariable region. These regions are marked with thick lines

A total of 105 known *S-RNase*s (Supplementary Table S2), including 6, 37, 21, and 41 from Solanaceae, *Malus*, *Pyrus*, and *Prunus*, respectively, and 23 RNase T2 family members from *F. vesca* and *F. viridis* (Table 1), which can encode long amino acid sequences (>100 amino acids), were selected for evolutionary analyses (Fig. 1). In contrast to *S*-RNase-like genes and *S*-RNase lineage genes^18^, *S*-RNase genes are evolutionarily orthologous^18,35,37^. Therefore, the evolutionary analysis indicated that the *S-RNase* candidates Unigene10929.1 and Unigene7320.1 experienced evolutionary pathways relatively similar to those of the *Prunus S-RNase*.

#### Conservative sequence analysis of *S*_*a*_ and *S*_*b*_-RNase in *F. viridis*

According to the transcript information, full-length specific primers were designed for the genes; CDS, FS_a_CDS, and RS_a_CDS were used to obtain Unigene10929.1, and FS_b_CDS and RS_b_CDS were used to obtain Unigene7320.1 (Supplementary Table S3). The PCR products were obtained using style cDNA as the template and were further sequenced. The sequences of both were identical to those obtained from the transcriptome. The nucleotide similarity of both candidates was 71.43% (Supplementary Fig. S5). Using *Prunus* as a reference, the results of the comparison of the deduced amino acids showed that Unigene10929.1 and Unigene7320.1 had five characteristic conserved *S*-RNase regions (C1, C2, C3, RC4, and C5) and a hypervariable region (RHV) (Fig. 1) and had similar amino acid differences in the regions between C1 and C2 and between RC4 and C5, in addition to similar intron locations and numbers^16,44^ (Fig. 1; Supplementary Fig. S6).

In summary, Unigene10929.1 and Unigene7320.1 conformed to the *S-RNase* and allelic similarity characteristics and were named *S*_*a*_-RNase and *S*_*b*_-RNase, respectively, for experimental verification. The *S*_*a*_-RNase and *S*_*b*_-RNase sequences were deposited in GenBank (accession numbers MW223017 and MW223018, respectively).

### DNA sequence analysis of *S*_*a*_ and *S*_*b*_-RNase

**Fig. 2:**
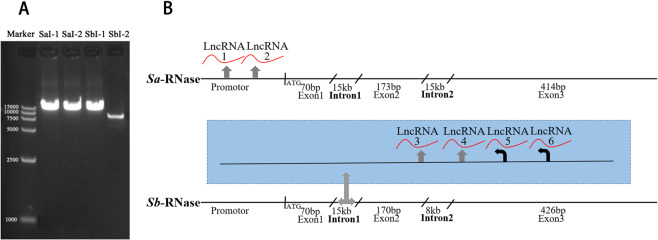
The structural pattern and potential neighboring lncRNAs of *S-RNase*. **A** S_a_I-1 and S_a_I-2 represent the first and second introns of *S*_*a*_-RNase, respectively; S_b_I-1 and S_b_I-2 represent the first and second introns of *S*_*b*_-RNase, respectively. The purified PCR product was used for DNA agarose gel electrophoresis. A 15-kb marker is provided on the left as a reference. **B** The area between the back slashes on the gene represents introns. The first intron of *S*_*b*_-RNase is magnified by a grid-filled arrow and highlighted with a blue background. The thick black line with arrow indicates the position and transcription direction of lncRNA. The analysis of the transcription direction of lncRNAs according to the characteristic structure of intron boundaries is also shown. The transcription direction could not be determined because there was no intron structure in lncRNA1-4, and the thick gray lines with arrows represent only the transcription positions

Based on the upstream and downstream primers designed against the adjacent exon region sequences, four intron PCR products of *S*_*a*_ and *S*_*b*_-RNase genes were obtained. The partial sequencing results at both ends of the fragments were consistent with the known sequences, so the resulting PCR products were the intended target segments. The first introns of *S*_*a*_ and *S*_*b*_-RNase were ~15 kb long, and the second introns of *S*_*a*_ and *S*_*b*_-RNase were ~15 kb and 8 kb long, respectively (Fig. 2A); these are rather long introns, which are rare in plants. Sanger sequencing of the PCR product library combined with high-throughput sequencing yielded the reference sequences of the first intron of *S*_*a*_-RNase, the first and second introns of *S*_*b*_-RNase, and part of the second intron of *S*_*a*_-RNase. In addition, we obtained the *S*_*a*_-RNase promotor sequence from splicing the sequences of the unmapped reads^45^ (Supplementary Dataset S1). The DNA reference sequences of *S*_*a*_- and *S*_*b*_-RNase were used as query sequences for comparison with the sequences of *F. viridis* style transcripts (Supplementary Dataset S2) using BLAST. The intron and promotor sequences were matched exactly by six sequences, among which two (lncRNA1 and lncRNA2) corresponded to the promotor region of *S*_*a*_-RNase, and four (lncRNA3, lncRNA4, lncRNA5, and lncRNA6) corresponded to the first intron of *S*_*b*_-RNase (Fig. 2B). The six sequences had short ORFs that did not correspond to a deduced protein sequence in the style protein library; by extension, they lacked protein-coding ability, which is consistent with the characteristics of lncRNAs. In addition, some of lncRNAs have promotor structures, or located in the promotor structure region of S-RNase (Supplementary Table S4, Supplementary Table S5). Using the DNA sequences matched with lncRNA1 and 2 to query raw data and mapping the reads to the DNA regions showed that the two lncRNAs were from different transcripts (Supplementary Fig. S7). However, the four other lncRNAs (LncRNA3, lncRNA4, lncRNA5, and lncRNA6) could not be confirmed as four independent transcripts because their corresponding DNA sequences partially overlapped. Taking the different introns of lncRNA5 and lncRNA6 as clues, these four lncRNAs may have come from the alternative splicing of the same transcript (Supplementary Fig. S7). The sequences of lncRNA1, lncRNA2, lncRNA3, lncRNA4, lncRNA5, and lncRNA6 can be found in Supplementary Dataset S2 (sequence IDs, Unigene18323_All, Unigene16020_All, Unigene26368_All, Unigene9260_All, Unigene25450_All, and Unigene12807_All, respectively). The DNA reference sequences of *S*_*a*_- and *S*_*b*_-RNase are provided as Supplementary Sequence S1 and Supplementary Sequence S2.

### Expression analysis of *S*-RNase in *Fragaria* tissues

**Fig. 3:**
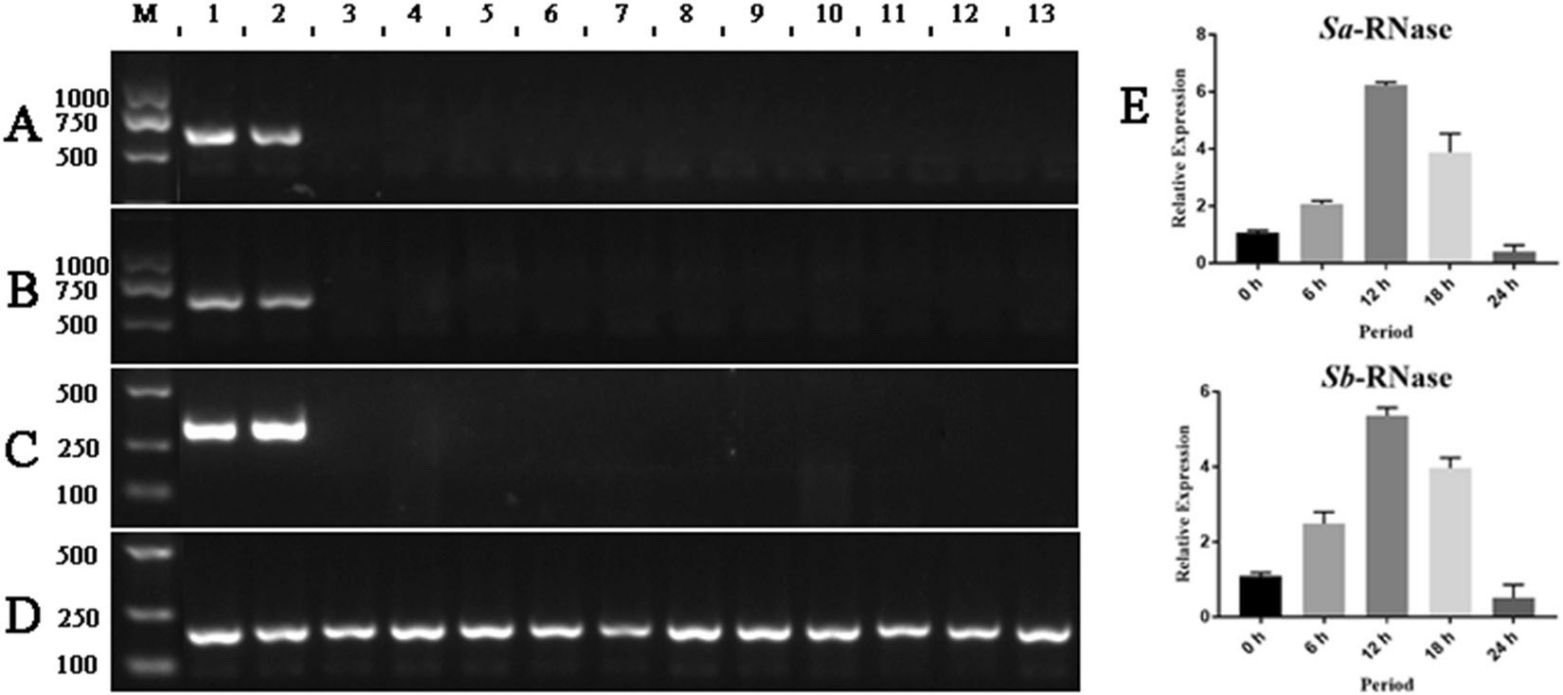
Analysis of *S*-RNase expression by RT and qRT-PCR. Lanes 1, 2, 3, 4, 5, 6, 7, 8, and 9 in **A**–**D** represent the flower balls, styles, ovaries, receptacles, pedicels, calyxes, petals, leaves, and anthers of *F. viridis* 42, respectively. Lanes 10, 11, 12, and 13 represent the flower balls of *F. vesca* 41, *F. mandshurica* 43, *F. nilgerrensis* 45, and *F.× ananassa* “Benihoppe”, respectively. **A**, **B** represent the expression of *S*_*a*_ and *S*_*b*_-RNase based on specific primers. **C** Shows the expression results obtained using degenerate primers for *S*_*a*_ and *S*_*b*_-RNase. **D** Represents the detection results of all cDNA samples with primers for a reference gene, *EF-1α*. **E** Illustrates the expression trends of *S-RNase* at different periods in styles after pollination. The abscissa represents the time after pollination, and the ordinate represents the level of expression

We analyzed the expression of *S-RNase* in styles, ovaries, receptacles, pedicels, calyxes, petals, leaves, and anthers. *S*_*a*_*-* and *S*_*b*_-RNase are specifically expressed in the styles and conform to the expression principles of style determinants; this also explains why the flower balls containing styles can be used for the screening and expression analyses of *S-RNase*. To test the expression of *S-RNase* in the different germplasms, the incompatible *F. viridis* 42 was selected as the control, while *F. vesca* 41, *F. mandshurica* 43, *F. nilgerrensis* 45, and *F*. ×*ananassa* “Benihoppe” were selected as the compatible species for analyses using specific and degenerate primers (FS_a_S_b_ and RS_a_S_b_) of *S-RNase* (*S*_*a*_ and *S*_*b*_-RNase), and the cDNA of the flower balls was used as a template. The results showed that *S*_*a*_ and *S*_*b*_-RNase were expressed only in *F. viridis* 42, and their expression was not detected in all compatible strawberry germplasms (Fig. 3A–D). The flower balls containing styles after pollination were used to test the spatiotemporal expression of the two genes at 6, 12, 18, and 24 h, and unpollinated flower balls were used as controls (Fig. 3E). The results showed that *S*_*a*_*-* and *S*_*b*_-RNase expression first increased and then decreased after pollination, which was consistent with the degree of pollen tube inhibition^45^, and the highest levels of expression were achieved 12 h after self-pollination.

### *S* locus mapping in *Fragaria*

**Fig. 4:**
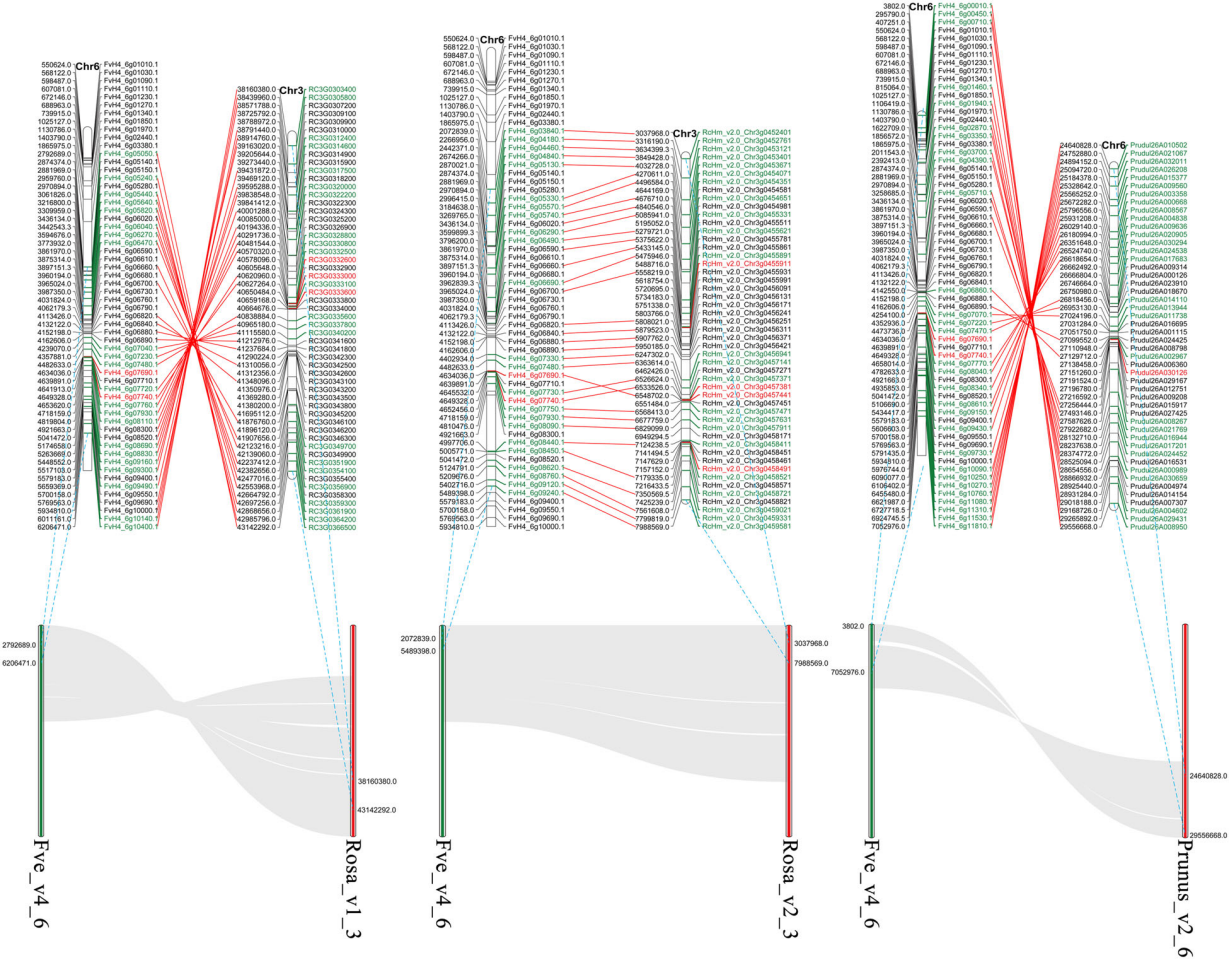
Analysis of the collinear region between the S loci of *Rosa* (rose) and *Prunus* (almond) and *F. vesca*. Fve_v4_6 represents chromosome 6 of *F. vesca*, and its enlarged region represents the area that is highly collinear with the *S* loci of the two rose genomes (Rosa_v1_3, from 38160380 to 43142292, and Rosa_v2_3, from 3037968 to 7988569) and the almond genome (Prunus_v2_6, from 24640828 to 29556668). The genomes of *F. vesca* and *Prunus* (almond) are represented by Fve_v4 and Prunus_v2, and the versions analyzed were the *F. vesca* genome v4.0.a1 and the *P. dulcis* “Texas” genome v2.0, respectively. Rosa_v1 and Rosa_v2 are rose genomes, and the versions analyzed were *Rosa chinensis* genome v1.0 and *Rosa chinensis* “Old Blush” homozygous genome v2.0, respectively. The numbers after the first and second “_” represent the genome version and the serial number of the chromosome, respectively. The red text on the right side of each chromosome represents the RNase T2 family genes, the black text represents the F-box family genes, the green text represents other types of genes, and the genes connected by red lines are those that were randomly selected for collinearity analysis in the enlarged area

The deciphering of plant genomes and the construction of databases has made data mining related to agronomic traits increasingly convenient^46,47^. Consistent with the results of previous studies^48,49^, we noted the high genome-wide collinearity of *Fragaria* with *Rosa* and *Prunus* (Supplementary Fig. S8), which provided favorable conditions for *S* locus location in *Fragaria*. The *S* loci were reported to be located on chromosomes 3 and 6 in the genomes of *Rosa* and *Prunus*, respectively^16,49^. We further analyzed the collinearity of *Rosa* (rose) chromosome 3 and *Prunus* (almond) genome chromosome 6 with *Fragaria* (*F. vesca*) and observed that both rose chromosome 3 and almond genome chromosome 6 had large collinear blocks with *F. vesca* chromosomes 1 and 6. However, further analysis revealed that the *S* loci of rose and almond had a common collinear region with *F. vesca* chromosome 6. In contrast, it had no collinear region with *F. vesca* chromosome 1 (Supplementary Fig. S9). The results indicated that there was only one *S* locus on chromosome 6 in *Fragaria* and that there was no *S* locus linked to SI on chromosome 1. We intercepted 2.5 Mb of *S-RNas*e flanking the rose and almond genomes as the corresponding *S* loci regions and analyzed the collinearity relationship between them and the *F. vesca* genome. The results showed that the *F. vesca* genome blocks that were collinear with the *S* loci regions of the Rosa_v1 rose genome, the Rosa_v2 rose genome, and the almond genome were ~3.4 (Chr: 2792689–6206471), 3.4 (Chr: 2072839–5489398), and 7.0 (Chr: 3802–7052976) Mb, respectively (Fig. 4). The overlapping area was 2.7 Mb (Chr: 2792689–5489398), which was located within 7.01–13.80% of the chromosome length, and this area was regarded as the *S* locus area in *F. vesca*. In addition, through whole-genome analysis of the gene families in *F. vesca*, we found two RNase T2 family genes (FvH4_6g07740, FvH4_6g07690) in the predicted *S* locus region. Notably, there were F-box gene clusters near the two RNase T2 family genes (Fig. 4, Supplementary Fig. S2), which was consistent with the linkage characteristics of the *S* locus genes (style and pollen determinants). However, these two RNase T2 family genes were not *S*-RNase genes (see the sections “S-RNase prediction in *F. viridis* and *F. vesca*” and “Evolutionary analysis of RNase T2 family members”).

### Identification of the *S-RNase* genotype of *F. viridis* 42

**Fig. 5:**
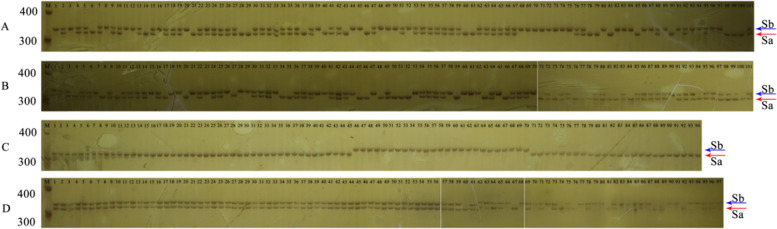
Identification of the *S-RNase* genotype by polyacrylamide gel electrophoresis. Each band indicated by a red arrow with the S_a_ tag is from *S*_*a*_-RNase, and each band indicated by the a arrow with the S_b_ tag is from *S*_*b*_-RNase. M represents the reference band for 300 bp and 400 bp markers. A1–101, B1–101, and D57–68 are the selfing progeny lines of “S1-02-S2-76-S3-09” (S_a_S_b_), and C1–45 and C71–94 are the selfing progeny lines of “S1-02-S2-30-S3-09” (S_a_S_a_). B46-70 are the selfing progeny lines of “S1-02-S2-76-S3-11” (S_b_S_b_). D1–56 are the hybrid progenies between “S1-02-S2-30-S3-09” (S_a_S_a_) and “S1-02-S2-76-S3-11” (S_b_S_b_). D69–97 are the selfing progeny lines of *F. viridis* 42 (0–3 generations). The serial number and order (from left to right and top to bottom) of the selfing lines are the same as those in Table 2

Polyacrylamide gel electrophoresis (PAGE) was used to identify nucleotide differences between the two genes, while genomic DNA was used as a template. Similar to that in *Pyrus* plants^15^, the SI of *F. viridis* is not complete, and a small number of progenies are derived from seeds contained in malformed fruits through self-pollination. First, a total of 29 lines, including wild *F. viridis* 42 (zero generation), selfed first generations, selfed second generations, and selfed third generations, were screened for the *S*_*a*_- and *S*_*b*_-RNase genotypes using degenerate primers (Table 2 and Fig. 5). The detection results were consistent with those obtained using CDS full-length specific primers and style cDNA as the template (Supplementary Fig. S10). There were six lines containing *S*_*a*_-RNase exclusively, 13 lines containing *S*_*b*_-RNase exclusively, and 10 lines containing both *S*_*a*_-RNase and *S*_*b*_-RNase. There were only three genotypes (S_a_S_a_, S_a_S_b_, and S_b_S_b_) without the *S*_*a*_- and *S*_*b*_-RNase null gene locus lines; therefore, we preliminarily considered *S*_*a*_- and *S*_*b*_-RNase as alleles. To further confirm that *S*_*a*_- and *S*_*b*_-RNase were alleles, we selected “S1-02-S2-76-S3-09” (S_a_S_b_) for self-crossing and obtained 214 progeny lines. The genotype was identified by FS_a_S_b_ and RS_a_S_b_. The genotype ratio was S_a_S_a_:S_a_S_b_:S_b_S_b_ = 48:106:60 ≈ 1:2:1. The progeny genotypes of “S1-02-S2-30-S3-09” (S_a_S_a_) following selfing were all S_a_S_a_, and the progeny genotypes of “S1-02-S2-76-S3-11” (S_b_S_b_) following selfing were all S_b_S_b_. The progenies of “S1-02-S2-30-S3-09” (S_a_S_a_) and “S1-02-S2-76-S3-11” (S_b_S_b_) crosses were all S_a_S_b_. *S*_*a*_- and *S*_*b*_-RNase conformed to the Mendel’s law of segregation, which confirmed that *S*_*a*_- and *S*_*b*_-RNase were alleles.

**Table 2:** S genotypes of *F*. *viridis* 42 and selfed lines

Line	S_0_	S_1_-01	S_1_-02	S_1_-03	S_1_-04	S_1_-05	S_1_-02-S_2_-02	S_1_-02-S_2_-11	S_1_-02-S_2_-28	S_1_-02-S_2_-30	S_1_-02-S_2_-35	S_1_-02-S_2_-37	S_1_-02-S_2_-49	S_1_-02-S_2_-53	S_1_-02-S_2_-57
Genotype	S_a_S_b_	S_b_S_b_	S_a_S_b_	S_b_S_b_	S_a_S_b_	S_a_S_a_	S_a_S_b_	S_a_S_a_	S_b_S_b_	S_a_S_b_	S_b_S_b_	S_b_S_b_	S_a_S_a_	S_a_S_b_	S_b_S_b_
Line	S_1_-02-S_2_-61	S_1_-02-S_2_-63	S_1_-02-S_2_-76	S1-03-S_2_-01	S1-05-S_2_-02	S_1_-02-S_2_-11-S_3_-06	S_1_-02-S_2_-30-S_3_-03	S_1_-02-S_2_-30-S_3_-05	S_1_-02-S_2_-30-S_3_-09	S_1_-02-S_2_-37-S_3_-01	S_1_-02-S_2_-76-S_3_-01	S_1_-02-S_2_-76-S_3_-02	S_1_-02-S_2_-76-S_3_-09	S_1_-02-S_2_-76-S_3_-11	
Genotype	S_b_S_b_	S_a_S_b_	S_a_S_b_	S_b_S_b_	S_a_S_a_	S_a_S_a_	S_b_S_b_	S_b_S_b_	S_a_S_a_	S_b_S_b_	S_b_S_b_	S_a_S_b_	S_a_S_b_	S_b_S_b_	

S_0_ represents *F. viridis* 42, a wild species, the 0th generation in the current study. S_1_, S_2_, and S_3_ represent the first-, second-, and third-generation selfed lines, respectively, and the specific number of each line is adjacent to the generation indicator. The S_a_S_b_ genotype contains both *S*_*a*_*-* and *S*_*b*_*-*RNase and is a heterozygous S genotype; S_a_S_a_ and S_b_S_b_ indicate genotypes containing *S*_*a*_*-* and *S*_*b*_*-*RNase only, respectively, and are homozygous S genotypes.

### Intraspecific hybridization between different S genotype lines of *F*. *viridis*

**Fig. 6:**
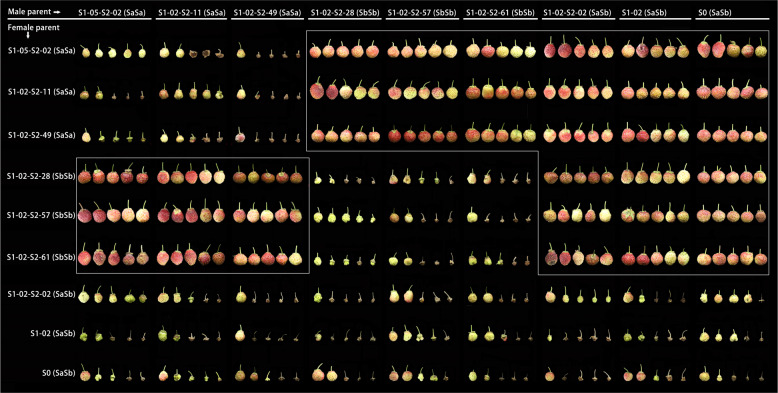
Fruit development following hybridization of different genotype lines from *F. viridis* 42. The S genotype lines were selected as shown in Table 2. The *S-RNase* genotype is shown in brackets

**Fig. 7:**
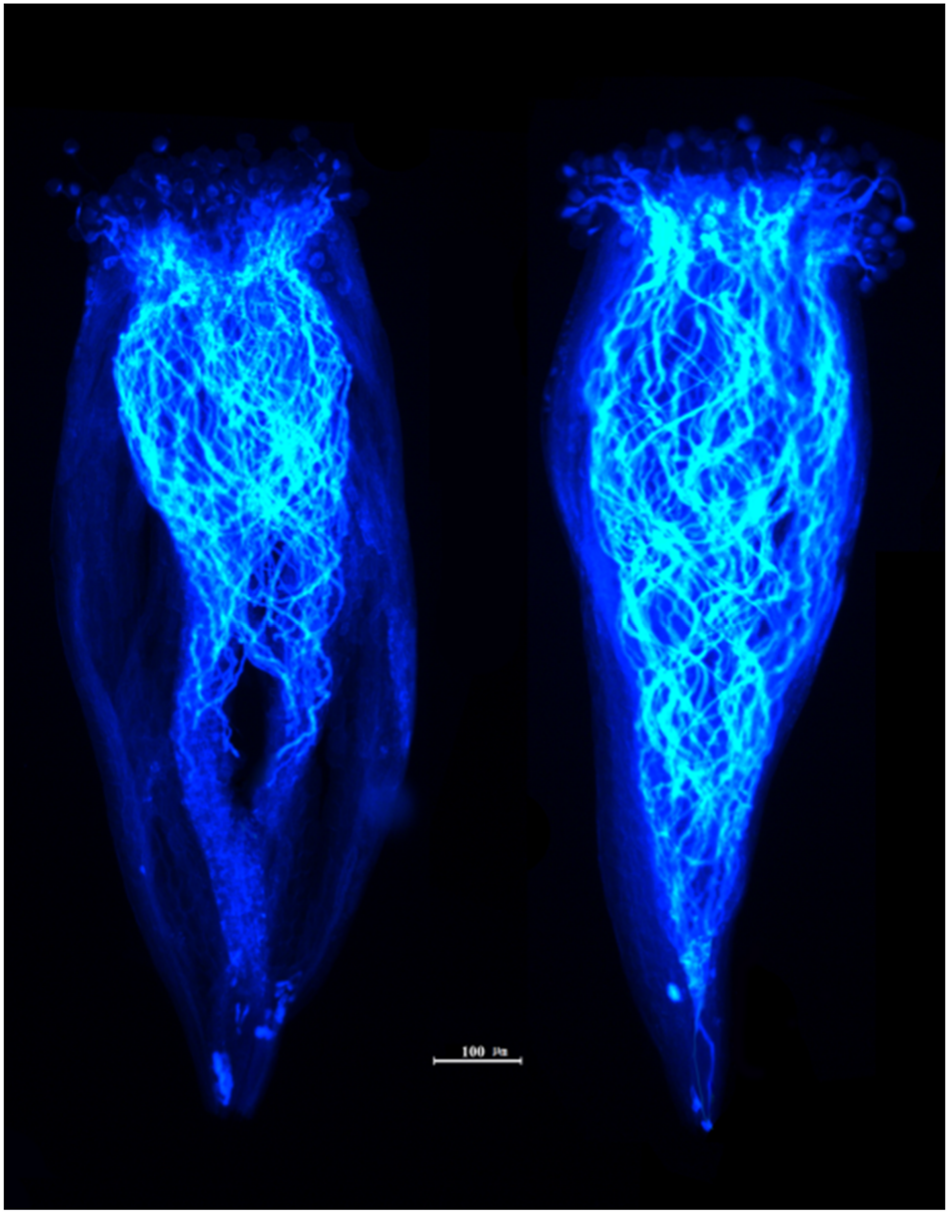
Growth status of compatible and incompatible pollen tubes in the styles of *F. viridis* lines 48 h after pollination. Scale bar = 100 μm. The left side shows pollen tube growth in the incompatible state, that is, the hybridizations that are not highlighted in “Fig. 6”. The right side shows pollen tube growth in the compatible state; that is, the hybridizations that are shown in boxes in “Fig. 6”

To verify the functions of *S*_*a*_- and *S*_*b*_-RNase in *F. viridis* SI, three genotypes, S_a_S_a_, S_b_S_b_, and S_a_S_b_, were selected to conduct selfing and intraspecific hybridization (Fig. 6, Supplementary Table S6). Each line was self-crossed, different lines of the same genotype were crossed with each other, and S_a_S_b_ genotype lines were used as the female parent in crosses with the S_a_S_a_ or S_b_S_b_ genotype lines. The hybridizations yielded abnormally developed receptacles with no fruit or malformed fruit, had low fruit-set and seed-set rates in single fruits, and were of the incompatible type. The S_a_S_a_ and S_b_S_b_ genotype lines were used as the female parent in crosses with the S_a_S_b_ genotype line. Intercrossing between S_a_S_a_ and S_b_S_b_ genotype lines yielded normally developed receptacles, a high seed-set rate, a fruit-set rate of 100%, no abnormalities, and a compatible type. In the incompatible hybridizations, most of the pollen tubes stopped at 2/3 of the length of the style 48 h after pollination (Fig. 7). In the compatible hybridizations, most pollen tubes extended to the base of the style, and some pollen tubes passed smoothly through the base of the style (Fig. 7). In addition, the color and development status of compatible and incompatible styles, receptacles, and achenes were different within 10 d of pollination (Supplementary Fig. S11). In summary, it has been confirmed that *S*_*a*_- and *S*_*b*_-RNase are determinants of style SI in *F. viridis*.

### Interspecific hybridization between *F. viridis*, *F. vesca*, *F. nilgerrensis*, and *F. mandshurica*

**Fig. 8:**
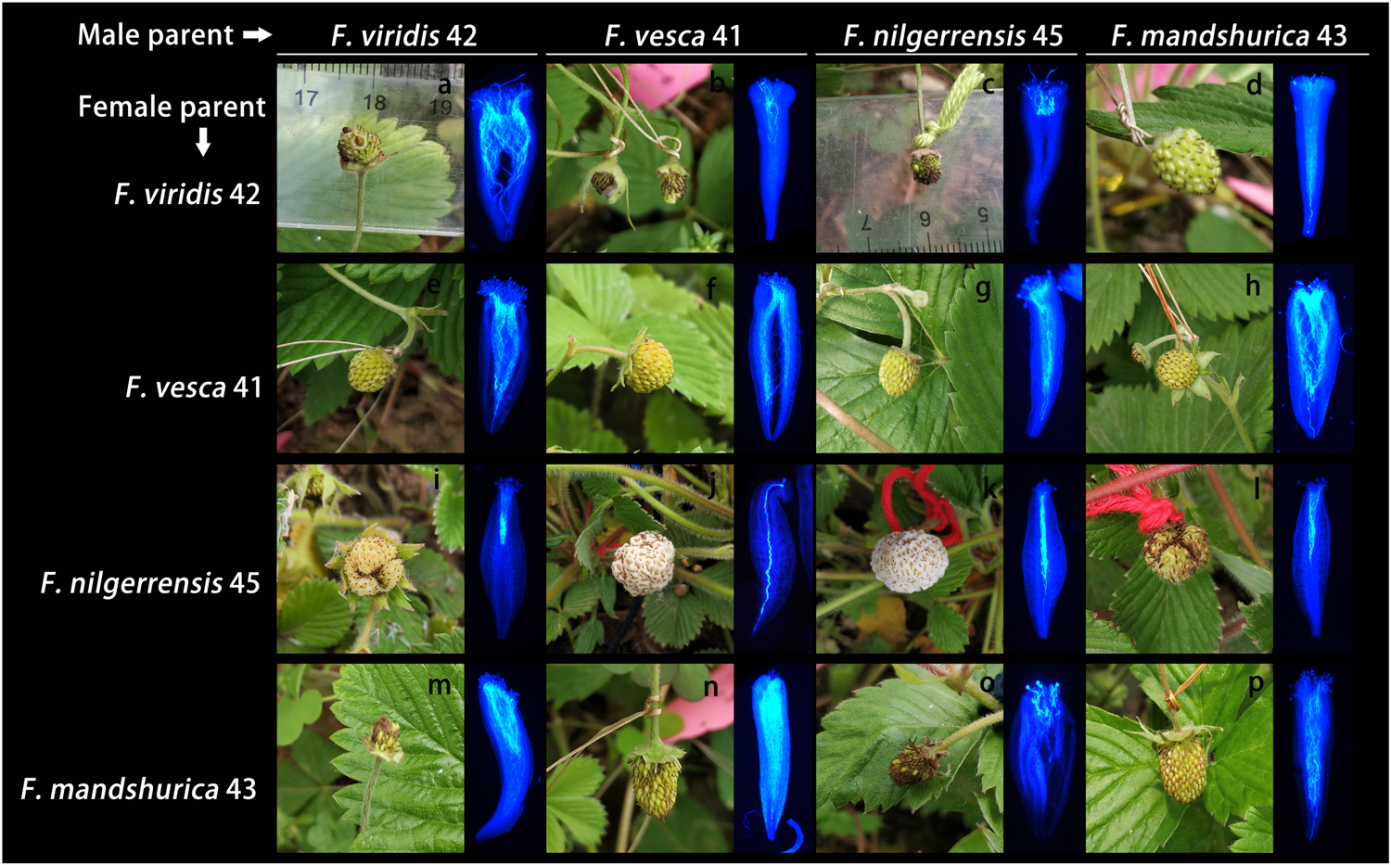
Fruit development and pollen tube extension in the style after interspecific cross-pollination. *F. viridis* 42 is a self-incompatible species, and *F. vesca* 41, *F. mandshurica* 43, and *F. nilgerrensis* 45 are self-compatible species. All are wild germplasm resources. Pictures were obtained 15–25 days after pollination, and the pollen tube was selected for observation 48 h after pollination

*F. vesca* 41, *F. mandshurica* 43, and *F. nilgerrensis* 45 can produce numerous seeds after self-pollination, with the receptacle developing normally, a fruit-set rate of 100%, and no malformed fruit; they, therefore, exhibit compatibility (Fig. 8, Supplementary Table S7). However, controversy persists about the (in)compatibility of *F. mandshurica*^29,30,50^, which was observed to be a self-compatible species according to our experimental data. When *F. viridis* 42, as the male parent, was crossed with *F. vesca* 41, *F. mandshurica* 43, and *F. nilgerrensis* 45, the single-fruit seed-set rates of *F. vesca* 41 and *F. nilgerrensis* 45 were high, the receptacles grew and expanded, and the crosses exhibited compatibility; these results are consistent with previous reports^1,5^. However, *F. nilgerrensis* 45 exhibited a less-developed receptacle, and the seed-set rate was significantly lower than that obtained from selfing. *F. mandshurica* 43 hardly set seeds, and the receptacle was not developed, which indicated incompatibility. *F. viridis* 42 was crossed as the female parent with *F. vesca* 41 and *F. nilgerrensis* 45. The results showed no receptacle development and almost no achene, which are considered signs of incompatibility. These results are consistent with the findings of the previous studies^1^. When *F. viridis* 42 was crossed as the female parent with *F. mandshurica* 43, receptacles developed normally, many achenes were produced, the fruit-set rate was 100%, and there was no malformed fruit. These results indicated compatible interspecific hybridization. When *F. vesca* 41 was crossed as the female parent with *F. nilgerrensis* 45 and *F. mandshurica* 43 or when *F. vesca* 41 was crossed as the male parent with *F. mandshurica* 43, the developed receptacles were normal, and the seed-set rate was high. These results indicate that these are compatible interspecific hybridizations. However, when *F. vesca* 41, as the male parent, was crossed with *F. nilgerrensis* 45, flower receptacle development and seed-set rate were not superior to those observed under *F. nilgerrensis* 45 self-pollination. When *F. mandshurica* 43 was crossed as the male parent with *F. nilgerrensis* 45, the receptacles enlarged and had a certain seed-set rate, which was also considered a sign of compatible hybridization. However, the values from this hybridization were considerably lower than those obtained from *F. nilgerrensis* 45 selfing. In the reverse crossing, there were almost no seeds, and the receptacle did not develop. These results indicate an incompatible hybridization.

The growth status of the pollen tubes in the styles was investigated at 48 h after self- and cross-pollination, and the results are shown in Fig. 8. In contrast to the incompatible case in *F. viridis* 42, the pollen tubes of *F. vesca* 41, *F. mandshurica* 43, and *F. nilgerrensis* 45 smoothly extended through the base of the style after self-pollination. Compatible interspecific hybridization (*F. vesca* 41, as the female parent, crossed with *F. viridis* 42, *F. mandshurica* 43, and *F. nilgerrensis* 45; *F. viridis* 42, as the female parent, crossed with *F. mandshurica* 43; *F. nilgerrensis* 45, and *F. mandshurica* 43, as the female parent, crossed with *F. vesca* 41) was found to be characterized by pollen tube growth patterns similar to those observed after the self-pollination of compatible germplasms. However, when *F. nilgerrensis* 45, as the female parent, was crossed with *F. viridis* 42 and *F. mandshurica* 43, the growth of most pollen tubes was restrained at 1/3–1/2 of the length of the style, and only a small number of uninhibited pollen tubes extended through the bases of the partial styles. In the incompatible interspecific hybridizations—when *F. mandshurica* 43 (female parent) was crossed with *F. viridis* 42 and *F. viridis* 42 (female parent) was crossed with *F. vesca* 41—the growth of most pollen tubes was restricted at 1/3–1/2 of the length of the style. In addition, when *F. viridis* 42 and *F. mandshurica* 43 (female parents) were crossed with *F. nilgerrensis* 45, the growth of most germinated pollen tubes was considerably inhibited, reaching only 1/5 of the length of the style. We also observed that pollen from *F. nilgerrensis* 45 exhibited lower germination rates than that from *F. vesca* 41 and *F. viridis* 42 on the stigma of *F. mandshurica* 43 and that the pollen tubes did not pass easily through the stigma.

## Discussion

### *S*-RNase-based GSI in the genus *Fragaria*

Relatively extensive research has been carried out on SI in the subfamily Amygdaloideae (apricot, Japanese apricot, plum, etc.) and the tribe Maleae (apple and pear)^17^. Strawberry plants belong to another subfamily, Rosoideae, in the family Rosaceae, and the mechanism of the regulation of incompatibility in this subfamily remains unclear. Based on the results of *S* genotype identification and cross-pollination experiments, *S*_*a*_- and *S*_*b*_-RNase are determinants of SI in the styles of *F. viridis*, and *S*_*a*_- and *S*_*b*_-RNase are alleles of the *S* locus. Furthermore, the genus *Fragaria* has an *S*-RNase-based SI type, which demonstrates the unproven GSI mechanism in Rosoideae and further advances our understanding of the SI system in Rosaceae. These observations are also in agreement with a recently published paper identifying the S-locus in the genus *Rosa*^51^. Most pollen tubes are generally suppressed at 1/3–1/2 of the style from the stigma when SI occurs^10,15,52,53^. Consistent with the results of previous studies^45^, the SI of *Fragaria* plants responds relatively slowly to pollen after pollination, implying some differences in the intermediate mechanism or the SI modification factors.

### Analysis of the SC mechanism in the genus *Fragaria* and insights into the *S-RNase* expression regulation mechanism

Notably, there are some self-compatible germplasms in the Rosaceae family with SI systems^1,15,17,54^. Such compatibility phenomena are relatively widespread in plants of the genus *Fragaria*, not only in cultivated species but also in many wild species, such as *F. vesca*, *F. nilgerrensis*, and *F. mandshurica*^1,6,29^. Consistent with previous reports, genome-wide RNase T2 family analysis has confirmed that no *S-RNase* has been observed in self-compatible species^6,18^. Further clues about the compatibility of the germplasm in the genus *Fragaria* were found through collinearity analyses. Two RNase T2 family genes were present at the identified *S* locus; however, the two genes did not conform to the characteristics of *S-RNase*. The conversion of a self-incompatible germplasm into a self-compatible germplasm often occurs in Rosaceae, typically due to variations in the genome sequence that result in the loss of gene fragments or gene functions of *S* determinants in the style or pollen^20,21,23,55–58^. Our results suggest that *S-RNase* was lost at the *S* locus of self-compatible germplasms during evolution.

*S*-RNase is expressed specifically in the styles and is maintained at higher levels after self-pollination, which is very important for the development of the SI phenotype^24,25^. However, few reports have explored the regulation of *S-RNase* expression, and only the promotor of *S-RNase* has been identified^59,60^. The self-incompatible *S* locus is located at the centromere (Solanaceae) or subtelomeric position on the chromosome. The region is often hypermethylated^61^, highly repetitive in its sequence, and heterochromatised^62^; these properties may make the DNA conservative and difficult to recombine and, in turn, restrict gene expression^61,63,64^. Fernández i Marti et al.^27^ showed that the methylation of the *S* locus in almonds is related to the loss of *S-RNase* function. In addition, the roles of introns in expression regulation have also been increasingly reported^26,29,65^. *S-RNase* in the genus *Fragaria* has a large intron and is currently the longest *S* gene; these characteristics are also rare in plant genes. However, whether such large introns are also involved in gene expression regulation requires further investigation. Some noncoding sequences can be expressed and play important roles in gene regulation^66,67^. Here, we detected the existence of related adjacent long noncoding RNAs near *S-RNase*, including the promotor and intron regions, which also provide further new clues for *S-RNase* expression regulation. All of these findings indicate the complexity of *S-RNase* expression regulation, which may involve multiple processes. However, much research work is still needed to clarify its mechanism.

### UI in *Fragaria* spp

**Fig. 9:**
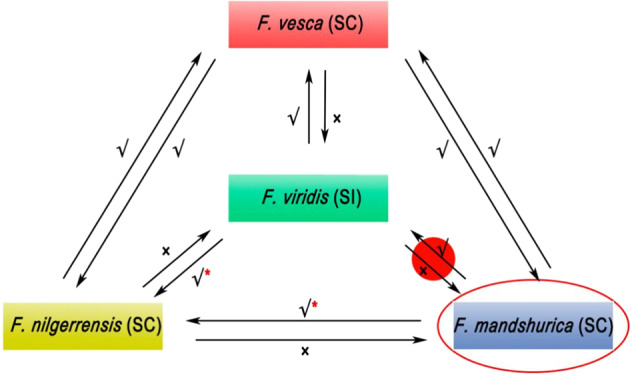
Hybridization of four wild strawberry diploid germplasms. The arrows in the diagram point to the female parents. √ denotes compatibility, × denotes incompatibility, and √* denotes hybridizations that were considered compatible in this study. In fact, the √* hybridizations are different from the other completely compatible interspecific hybridizations (that is, the receptacle develops normally, the number of achenes is high, and most germinated pollen tubes can reach the bases of styles), although a certain compatibility was observed based on receptacle development and achene level. In the √* interspecific hybridizations, most pollen tubes were inhibited at 1/3–1/2 the length of the style, and only some of the styles facilitated pollen tubes without restriction

Generally, UI between SC and SI species arises when an SI species, as a pollen donor is crossed, with an SC species and is compatible, however the reciprocal cross occurs, and it is manifested as an incompatibility^7^. Among the three interspecific hybridizations between *F. viridis* and *F. vesca*, *F. mandshurica*, and *F. nilgerrensis*, two hybridizations conformed to the rule above and were consistent with previous research results, suggesting that the UI is related to the *S*-RNase-based SI^1,6,68^. However, when *F. viridis* was crossed with *F. mandshurica*, the result was contrary to the SI × SC rule and was not influenced by the S genotype (Fig. 8, Supplementary Table S7). Pollen rejection under interspecific hybridization systems is complex and involves multiple pathways. In addition to the UI that conformed to the SI × SC rule, there are other UI barriers that are not related to SI^69,70^. An SI-independent mechanism exists not only between compatible germplasms (such as *F. nilgerrensis* and *F. mandshurica*) but also between SI and SC germplasms (such as *F. viridis* and *F. mandshurica*) (Fig. 9). The discovery of an exception to the SI × SC rule that is in force in *Fragaria* and the results of the investigation of the UI between compatible species provide a basis for the analysis of *Fragaria* UI mechanisms.

In other genera, including *Solanum* and *Pyrus*, cross-compatible pollen tubes can reach the base of the style, and some even pass through the style bottom. However, interspecific incompatible pollen tubes often stop growing within 1/3 of the length of the style^7,10,15,52^. In *Solanum*, interspecific incompatible pollen tubes are arrested earlier than self-incompatible pollen tubes^7^; a similar phenotype was also observed in *Fragaria*. There were also some differences in the inhibition time between different interspecific hybridizations in *Fragaria* plants. Some interincompatible pollen tubes (1/3–1/2) in *Fragaria* are suppressed later than those in Solanaceae^7^ (within 1/3 of the length of the style); however, some interspecific hybridizations are stopped earlier. Consistent with previous results, *F. vesca* accepts pollen from other strawberry species relatively easily^1,5,31^. In the present study, *F. nilgerrensis* had a lower capacity to accept pollen from germplasms other than *F. vesca*, and the intensity of pollen suppression was greater in the pistils of other germplasms. The functional structural units involved in incompatibility include the pistil-side barrier and pollen-side resistance. Additionally, incompatibility occurs when pollen resistance is lacking or is weaker than the pistil-side barrier^7,8,71^. The interspecific hybridization barrier is also a relatively primitive state that prevents gene exchanges among related germplasms^72^. We speculate that when the pistil-side barrier in *F. vesca* weakened, the style of *F. nilgerrensis* maintained a certain capacity to overcome pollen resistance, and the pollen developed relatively weak resistance during evolution. These results explain why *F. vesca* is a reasonable candidate intermediate germplasm for gene exchange and why *F. nilgerrensis* 45, in a compatible interspecific hybridization, hinders most of the pollen tubes of *F. viridis* and *F. mandshurica*. They also explain why the pollen tubes of *F. nilgerrensis* are strongly restricted in the styles of *F. viridis* and *F. mandshurica* in incompatible interspecific hybridizations. Moreover, the phylogenetic evolution of *Fragaria* shows that *F. nilgerrens* has a distant relationship with *F. viridis* and *F. mandshurica*^73^. Thus, the influence of phylogenetic distance on the compatibility of interspecific hybrids cannot be ruled out.

### The control loci of S-RNase-mediated GSI

Bosković et al.^6^ hypothesized that two *RNase* loci (S and T) can explain the regular occurrence of SI and UI in the genus *Fragaria*. However, this hypothesis—that SI is controlled by *S*-RNase—is constrained by the fact that not all style-expressed RNases are *S*-RNases^18,74^. Sequence information for these two loci has not been obtained. In addition, peptide sequences that have been previously used to demonstrate that the S and T loci encode RNase proteins are controversial^6,18^. In contrast to previous findings, only two allelic *S-RNase*s could be identified using the deduced style protein database of *F. viridis* based on information regarding the characteristics of *S*-RNase, and the two genes were identified as style SI determinants using the hybridization experiment; these findings indicate that only one *S* locus plays a role in SI in the genus *Fragaria*. Based on genome-wide collinearity analysis, the *S* locus regions of the almond and rose genomes had only one collinear region with the *F. vesca* genome, on chromosome 6 (Chr6: 2792689–5489398). Pgl1 exists in this region (Supplementary Fig. S9), which is consistent with the previously reported T locus^6^. The *S* locus predicted using collinearity analysis is also located at the far end of the chromosome, which is consistent with the subtelomeric region where the *S* locus of the species is observed in families other than Solanaceae^15,16,61^. Bosković et al.^6^ mentioned an *S* locus linked to AC8 on chromosome 1; collinearity analysis also shows that chromosome 1 of the genus *Fragaria* has regions that are collinear with the chromosomes where the *S* locus is found in almond and rose. However, the two pairs of collinear regions do not overlap in the *Fragaria* genome and are not located in the *S* locus region in the genomes of almond and rose. The collinearity between the non-S locus chromosomal region of the chromosome where the *S* loci of the *Prunus* and *Rosa* genomes are located and chromosome 1 of *Fragaria* might be the result of chromosomal exchange between chromosomes 1 and 6 of *Fragaria*^49,75^. The AC8 genetic marker is located in the region that is collinear between *Fragaria* and *Prunus* (Supplementary Fig. S9) and may therefore reflect a false *S* locus.

## Materials and methods

### Materials and sample selection

The incompatible wild *F. viridis* 42 was used to establish a style expression database and to explore style SI determinants in the genus *Fragaria*, and its selfed progenies were used for S genotype identification and functional verification by cross-pollination. To explore the reasons for the compatibility of certain strawberry germplasms, we selected *F. vesca* 41 (SC), *F. nilgerrensis* 45 (SC), *F. mandshurica* 43 (SC), and *F.× ananassa* “Benihoppe” (SC) for an analysis of the expression of *S*-RNase using RT-PCR. This was accompanied by cross-pollination experiments among four wild germplasms to further analyze the role played by *S*-RNase in the interspecific hybridization of the genus *Fragaria*. All resources used in the experiments were stored at the Baima Teaching and Research Base of Nanjing Agricultural University in Baima Town, Nanjing City, Jiangsu Province, China. Strawberry resources grown in the field (from March 2019 to June 2019) were used for selfing, cross-pollination, and sample collection. The temperature during this period was suitable for strawberry growth and enabled the plant materials to bloom normally. In addition, the flowering periods of the four *Fragaria* species partially overlapped. Various flowering stages differentially influence pollination results; therefore, we specifically selected flowers at the large bud stage (C3–C5) (Supplementary Fig. S12) for the pollination experiments.

### Total RNA extraction, cDNA preparation, and Illumina sequencing

A total of six biological samples—three *F. viridis* flower balls (Supplementary Fig. S12) at 0 and 24 h after self-pollination—were harvested. Each sample included 10 flower balls, and all samples were stored in liquid nitrogen for RNA extraction. Total RNA was extracted using TRIzol reagent (TaKaRa, Dalian, China). RNA quality was evaluated using the Agilent 2100 RNA 6000 Kit. This RNA was then reverse transcribed into cDNA using SuperScript III Reverse Transcriptase (Invitrogen, CA, USA). Then, cDNA libraries were sequenced on an Illumina HiSeq^TM^ 4000 platform at the Beijing Genomics Institute (BGI, Shenzhen, China).

### Analysis of RNase T2 and F-box gene family members

According to the hidden Markov model (HMM) of RNase T2 and the F-box domain, the hmmsearch program in Hmmer software (http://hmmer.org/download.html)^76^ was used to search the genomic protein database of *F. vesca* and *F. viridis* to obtain the RNase T2 and F-box gene family members. To verify the integrity of the protein domains and filter out proteins that did not contain RNase T2 domains, the selected proteins were checked using the National Centre for Biotechnology Information (NCBI) Conserved Domains Database (https://www.ncbi.nlm.nih.gov/cdd/?term). The molecular weight and isoelectric point of the obtained RNase T2 family members were analyzed by ExPaSy (https://web.expasy.org/protparam/)^77^, and the signal peptides were analyzed by SignalP (http://www.cbs.dtu.dk/services/SignalP/index.php)^78^. Meanwhile, alignment analysis of the amino acid sequence was performed using DNAMAN8.0 (Lynnon, QC, Canada).

The RNase T2 HMM can be obtained via two methods. One is by downloading the RNase T2 model file (PF00445, RNase T2 HMM2) directly from the Pfam database (http://pfam.xfam.org/), and the other is by constructing an RNase T2 HMM using a known *S*-RNase (Supplementary Table S2). The *S*-RNases of Solanaceae, *Malus*, *Pyrus*, and *Prunus* were downloaded by entering the respective keywords (family or genus name and *S*-RNase) in the search window of the NCBI database. According to the annotations, some genes with unclear functions or incomplete sequences were omitted from the analysis, and the more reliable *S-RNase* genes, which were recognized *S*-RNase family members (Supplementary Table S2), were retained. The F-box HMM model file (PF00646, F-box HMM) was obtained directly from the Pfam database.

The proteome data for *F. vesca* were downloaded from the Genome Database for Rosaceae (https://www.rosaceae.org/), and the *F. vesca* genome v 4.0.a1 was used. The *F. viridis* style proteome was predicted based on the style transcriptome (Supplementary Dataset S2). The ORF finder script^79^ was used to predict the open reading frames (ORFs) (Supplementary Dataset S3) of the spliced transcripts and to deduce the corresponding protein sequences (Supplementary Dataset S4). De novo assembly was performed using clean reads from raw data with Trinity software^80^ for the transcripts. Afterward, the transcripts of all samples were merged (Supplementary Dataset S2).

The *S-RNase* in Supplementary Table S2 and the RNase T2 family members with longer protein lengths (>100 amino acids) from *F. vesca* and *F. viridis* were subjected to multiple sequence alignment using the MUSCLE program in MEGA 7.0^81^. A phylogenetic tree was constructed using MEGA 7.0, while the neighbor-joining method was adopted for cluster analysis, with the number of bootstrap replicates set to 1000. EvolView v2 (https://evolgenius.info//evolview-v2/#login) was used to improve the display of the evolutionary tree as well as to add group labels and colors. According to the results of the evolutionary analysis, the species closely related to the candidate *S*-RNases of *F. viridis* were selected in order to analyze the conservative structure of the candidates.

### Cloning and sequence analysis of Sa-RNase and Sb-RNase

RNA was extracted from the flower balls containing the styles of *F. viridis* 42 (Supplementary Fig. S12) using an RNAprep Pure Plant Plus Kit (TIANGEN, Beijing, China) according to the manufacturer’s instructions. The kit is suitable for Rosaceae plants, which have tissues with high polysaccharide and polyphenolic compound contents. The extracted total RNA was digested with RNase-free DNase (TaKaRa, Dalian, China) to obtain high-purity RNA, and then the concentration was measured using a NanoDrop 2000 ultraviolet-visible spectrophotometer (Thermo Scientific, Waltham, MA, USA). The PrimeScript^TM^ RT Reagent Kit (TaKaRa, Dalian, China) was used to reverse transcribe the RNA into cDNA, according to the manufacturer’s instructions. After reverse transcription, the appropriate amount of ddH_2_O was added to dilute the mixture to 200 ng/µL based on the desired concentration. Specific primers were designed (Supplementary Table S3), including FS_a_CDS and RS_a_CDS for *S*_*a*_-RNase and FS_b_CDS and RS_b_CDS for *S*_*b*_-RNase, with cDNAs as templates for the CDS full-length sequences. PrimeSTAR^®^ GXL DNA Polymerase (TaKaRa, Dalian, China) was used in the reaction. The amplification procedure for the *S*_*a*_-RNase was as follows: 1 cycle (98 °C for 5 min), 36 cycles (98 °C for 10 s, 50 °C for 15 s, and 68 °C for 40 s), a final extension at 68 °C for 10 min, and storage at 10 °C. The annealing temperature of *S*_*b*_-RNase was 53 °C. The temperature conditions for the other reactions were similar to those described above. The PCR products obtained after the addition of a base (A) at both ends of the obtained flat-end sequences were analyzed on 1.5% agarose gels, and the putative fragments were purified using a DNA purification kit (TaKaRa, Dalian, China). The procedure for adding the “A” base was conducted at 72 °C for 30 min using rTaq (TaKaRa, Dalian, China). The purified fragment was cloned into the pMDTM19-T vector (TaKaRa, Dalian, China) and transformed into *E. coli* DH5α competent cells (Tsingke, Nanjing, China). Ten monoclones per gene were sequenced by a biotechnology company (Tsingke, Nanjing, China). The sequence obtained was compared with the sequence from the transcriptome using DNAMAN v8.0 (Lynnon, QC, Canada).

The target PCR product could not be obtained from the genome using cloning primers designed with the full-length CDS of *S-RNase*. This indicated that *S-RNase* may contain large introns. To obtain genetic information regarding *S*-RNase, the nucleotide sequences of *S-RNase* were first analyzed to identify the exon-intron structure of the gene. Then, specific primers against the intron were designed at the intron-exon boundary of the gene using the segmented cloning approach. The GXL enzyme was employed to amplify the target fragment using a rapid reaction program. The annealing temperature was 60 °C, and the extension time was 1 min and 50 s. Fresh young leaves of *F. viridis* were collected and stored at −80 °C in an ultralow-temperature refrigerator after quick freezing in liquid nitrogen. gDNA was extracted using the Super Plant Genomic DNA Kit (TIANGEN, Beijing, China), which is suitable for plants with high polysaccharide and polyphenolic contents. The following steps were used to design primers for *S-RNase* cloning: (1) the quality of the original reads (paired-end sequences) obtained from whole-genome resequencing data^45^ of Ls-S2-53 was evaluated and filtered to obtain clean reads; (2). SOAPdenovo 2^82^ was used to assemble clean reads that were not mapped to the reference genome, and a series of contigs and scaffolds were obtained as the database sequences; (3) *S*_*a*_ and *S*_*b*_-RNase transcripts representing query sequences were aligned with database sequences, and the boundaries of introns and exons were defined by analyzing the alignment results between *S-RNase* and its matched genome splicing sequence; and 4) after clarifying the number and positions of introns, upstream and downstream primers were designed against the first exon and the second exon or their matching sequences (contigs and scaffolds) and were used to clone the first intron; additional primers were designed against the second exon and the third exon or their matching sequences in order to clone the second intron. The primers used to clone the first and second introns of *S*_*a*_-RNase were FS_a_Intron1 and RS_a_Intron1 and FS_a_Intron2 and RS_a_Intron2, respectively. The primers used to clone the first intron of *S*_*b*_-RNase were FS_b_Intron1 and RS_b_Intron1, and those used to clone the second intron were FS_b_Intron2 and RS_b_Intron2. The primer sequences are listed in Supplementary Table S3.

### *F. viridis* S genotype identification

Using specific primers against the CDS (full-length) of *S*_*a*_- and *S*_*b*_-RNase and style cDNA after 12 h of self-pollination (template), the genotypes of the materials were determined (Table 2). The S alleles exhibited a certain degree of polymorphism, and the fragments of different lengths could generally be obtained using degenerate primers. To obtain more feasible genotypes, we also designed degenerate primers for the relatively conserved regions of the two sequences using DNA as a template to test the materials listed in Table 2 again. The degenerate primer was designed against the third exon of the *S-RNase*, and the product of *S*_*b*_-RNase was 9 bp larger than that of *S*_*a*_-RNase. Green Taq^TM^ Mix (Vazyme, Nanjing, China) was used in combination with degenerate primers FS_a_S_b_ and RS_a_S_b_ in the PCR. The amplification procedure was as follows: 95 °C for 5 min and 36 cycles (95 °C for 15 s, 60 °C for 15 s, and 72 °C for 40 s), a final extension step at 72 °C for 5 min, and storage at 10 °C. The PCR product was diluted 10 times, and 2 µL was examined after staining with bromophenol blue in a 10% polyacrylamide gel for electrophoresis. The running procedure was as follows: 90 v pre-electrophoresis for 15 min, 160 v electrophoresis for 1.5 h, pause for 30 min, and 160 v electrophoresis again for 1.5 h. The polyacrylamide gel was soaked in silver staining solution (1 g AgNO_3_ + 33 mL C_2_H_5_OH + 3 mL CH_3_COOH + 750 mL ddH_2_O) for 15 min and then soaked in a developer solution (10 g NaOH + 4.5 mL HCHO + 750 mL H_2_O) for more than 10 min after twice cleaning with ddH_2_O and subsequently photographed under white light. In addition to the selfing lines in Table 2, degenerate primers were also used to test the selfing progenies’ S genotypes, including S1-02-S2-30-S3-09, S1-02-S2-76-S3-09, and S1-02-S2-76-S3-11, and the S genotypes of the hybrid progenies between S1-02-S2-30-S3-09 and S1-02-S2-76-S3-11.

### *S*-RNase expression analysis

Using *F. viridis* 42 as the control, *S*-RNase expression in compatible strawberry species, such as *F. vesca* 41, *F. nilgerrensis* 45, *F. mandshurica* 43, and *F.× ananassa* “Benihoppe”, was tested. Total RNA was extracted from the flower balls, and cDNA was synthesized for *S*-RNase expression detection. The detection primers were specific primers used to clone the full CDS of *S*_*a*_ and *S*_*b*_-RNase and their degenerate primers. At the same time, the styles, ovaries, and receptacles of the flower balls, as well as the petals, calyxes, pedicels, leaves, and anthers, were collected for tissue-specific expression analysis of *S*_*a*_- and *S*_*b*_-RNase. Taking the unpollinated flower balls as the control, spatiotemporal expression analysis of *S*-RNase after pollination was performed at 6 h, 12 h, 18 h, and 24 h. The expression analysis of *S*-RNase in a different germplasm and different tissues was performed using RT-PCR, while spatiotemporal expression analysis was performed using qRT-PCR. The RT-PCR is detailed in the section “Cloning and sequence analysis of *S*_*a*_-RNase and *S*_*b*_-RNase”, and the qRT-PCR method is detailed here. First, the synthesized cDNA was diluted to 100 ng/µL using SYBR Premix Ex TaqTM (TaKaRa), and then an ABi 7500 fluorescent quantitative PCR system (Applied Biosystems, Bedford, MA, USA) was used to perform qRT-PCR. The internal reference primer was the elongation factor-α gene EF1-α. Each reaction consisted of a 20 µL volume containing 10.0 µL SYBR Premix Ex TaqTM (TaKaRa), 1 µL cDNA, 0.5 µL of each primer (10 µM), and 8 µL ddH_2_O. The reaction conditions were as follows: 95 °C for 4 min and 40 cycles (95 °C for 20 s, 60 °C for 20 s, and 72 °C for 40 s). The upstream and downstream primers were designed on the basis of the second and third exons of *S*-RNase, respectively. The primer pairs used to detect *S*_*a*_-RNase were FS_a_k and RS_a_k, and RS_b_k and FS_b_k were used to detect *S*_*b*_-RNase (Supplementary Table S3). Each reaction had three technical replicates, and the 2^−ΔΔCt^ method was used to evaluate the relative expression of genes.

### Pollination test and compatibility analysis of different hybridizations within and between species

The anthers were removed from the large flower buds collected from 7 to 9 o’clock in the morning and were wrapped in sulfuric acid paper and placed in silica gel bottles. Afterward, they were stored in a 4 °C refrigerator. The fully dried pollen was used in the pollination experiments or stored in a −70 °C refrigerator. Generally, on successive sunny days at warmer temperatures, strawberry plant buds (at stages C3–C5) were selected for artificial emasculation and isolated using pollination bags in the afternoon. On the following morning, starting at 9 am, artificial pollination was performed, and the flowers were isolated again. Generally, pollen with good germination capability, as determined using pollen activity tests, is used in pollination experiments. The pollen germination medium was slightly modified based on a previous method^45^, and 0.01% GA_3_ was added. Eleven flowers were selected for each set of pollination experiments, and one of the eleven flowers was collected 48 h after pollination to observe the growth status of pollen tubes in the style. The remaining flowers were generally removed from the pollination bags after pollination for ~10 d, and the pollen tube growth status in the style was observed with a previously described dyeing method^45^.

Receptacle development was observed, and the fruit-set and seed-set rates of single fruits were determined after 25 d via intraspecific pollination experiments. In addition, the (in)compatibility of different pollination hybridizations was analyzed by examining the overall growth status of the pollen tubes in the different styles. Three *F. viridis* lines were selected for use in the intraspecific pollination experiments for each S genotype, and one line from the different S genotypes was selected for use in the interspecific pollination experiments. In the interspecific hybridizations, all germplasms were self-compatible, excluding that of *F. viridis*. The flowering times of the different germplasms were slightly different, and the rate of development of the receptacle also varied across the germplasms. Typically, photographs of the fruit were taken 15–25 d after pollination, and the fruit-set rate following the interspecific hybridizations was determined; however, the seed-set rate of single fruits was determined after fruit maturity. In addition, for the intraspecific hybridization of *F. viridis*, mutual pollination was performed with blooming flowers of different S-genotype lines using the smearing method without the influence of external pollinators in the greenhouse, and the development and color changes of the receptacles and achenes were observed within 10 d of pollination.

### Collinearity analysis and *S* locus location in the genomes of the genera *Fragaria, Rosa*, and *Prunus*

Genome-wide collinearity analysis was performed between the *F. vesca* and rose and almond genomes as well as between the two rose genomes using BLAST, while MCScanX^83^ and Ciros (http://circos.ca/) were used to illustrate collinearity. The genomes and the associated versions used were as follows: *F. vesca* genome v4.0.a1^84^, *Rosa chinensis* genome v1.0 (Rosa_v1)^49^, *Rosa chinensis* “Old Blush” homozygous genome v2.0 (Rosa_v2)^85^, and *P. dulcis* “Texas” genome v2.0. The chromosomes harboring the *S* locus in almond and rose were used to further analyze collinearity with the whole genome of *F. vesca* to obtain the collinearity blocks distributed in *F. vesca*. Furthermore, based on whether the collinearity blocks in *F. vesca* exhibited collinearity with the S locus of almond and rose, the numbers and positions of the S loci in the genome of *Fragaria* were further determined. Based on the location of *S-RNase* in the reported almond genome, Rosa_v1 rose genome^49^, and the evaluated Rosa_v2 rose genome (Supplementary Fig. S13), the *S* loci areas were obtained by extending the 3’ and 5’ termini of *S*-RNase by ~2.5 Mbp. The *F. vesca* genome regions that exhibited collinearity with the *S* locus of the almond genome, Rosa_v1 rose genome, and Rosa_v2 rose genome were selected to perform a more accurate location analysis. The reason for compatibility in strawberry was further determined through a functional analysis of RNase T2 family members and the distribution of F-box family members at this locus.

## Supplementary information

Supplementary Figures S1-S13, Tables S1-S7 and Sequence S1-S2

Dataset S1

Dataset S2

Dataset S3

Dataset S4
