# Supplementary material for: Molecular characteristics of S-RNase alleles as the determinant of self-incompatibility in the style of Fragaria viridis
Source: Hortic Res. 2021 Aug 1;8:185. doi: 10.1038/s41438-021-00623-x (PMC8325692; doi:10.1038/s41438-021-00623-x)
Supplement: Supplementary file 1 — Supplementary Figures S1-S13, Tables S1-S7 and Sequence S1-S2 [file 41438_2021_623_MOESM1_ESM.docx]

**Supplementary data**

**Supplementary Fig. S1. The size and approximate location of the domains contained in RNase T2 family genes of *F. vesca* and *F. viridis*.**

All genes selected from the *F. vesca* and *F. viridis* databases are used to analyze the integrity of the RNase T2 domain. The left side is the gene name, the right side is the size of the domain, the length of the green band indicates the size of the structure, and the two ends indicate the start and end of the domain, respectively. The top ruler is represented by the number of amino acids, and the bar closest to the ruler represents the RNase T2 reference model. The complete RNase T2 domain is 182 amino acids in size.

**Supplementary Fig. S2. Genome-wide chromosomal mapping of RNase T2 and F-box family genes in *F. vesca*.**

The number on the left represents the specific position of the gene on the chromosome, the black font on the right represents the F-box family gene, the red font represents the RNase T2 family gene, and the bottom notes is the chromosome number. The gene location information is obtained from the *F. vesca* database GFF file, the location of genes on the chromosomes is graphically using Mapchart. There are 2 RNase T2 family genes distributed on chromosome 1, and 3 RNase T2 family genes on each chromosome 2, 4, 5 and 6, and 61, 70, 87, 51, 47, 114, 41 F-box genes on chromosome 1, 2, 3, 4, 5, 6 and 7 (selected by F-box Markov model only), respectively. S-RNase and SFB/SFBB/SLF genes are often located within a range on the S locus, but the distance between both genes has greatly difference depending on the S genotype. The physical distance of the S haplotype of Solanaceae is significantly larger than that of Rosaceae, and the physical range of the S haplotype of Solanaceae is about 4 Mb^1^. The physical distances between different species of Rosaceae are not the same, both *Malus* and *Pyrus* are located on chromosome 17, and the size is about 1Mb and 649Kb respectively^2^. In almonds (*P. dulcis*), the range is within 70 Kb^3^ and the SFB alleles within 30 Kb^4^, and in sweet cherries (*P. avium*), from 380 bp to 40 Kb^5,6^. Therefore, we used the larger distance range of Rosaceae as a reference to analyze the existence of F-box gene flanking the RNase T2 gene. There is no F-box gene within the 500 Kb flanks of FvH4_2g25650.1, FvH4_2g25620.1, FvH4_4g18130.1, FvH4_5g24550.1, FvH4_5g33850.1 and FvH4_5g24800.1, and no F-box gene within 380 Kb flanks of FvH4_2g17310.1 and FvH4_6g22290.1. FvH4_1g19170.1, FvH4_1g10040.1, FvH4_4g31300.1, FvH4_4g31290.1, FvH4_6g07740.1 and FvH4_6g07690.1 have F-box genes in the close physical distances.

**Supplementary Fig. S3. Chromosome location analysis of RNase T2 family genes in *F. viridis*.**

Blastn tool (https://www.rosaceae.org/blast/nucleotide/nucleotide) was used to perform gene mapping analysis of RNase T2 family members obtained from *F. viridis* taking the *F. vesca* genome_v4.0.a1 as the reference genome. Among the RNase T2 family genes of *F. viridis*, 11 genes have been assigned to the reference genome, and two genes have not been mapped to the genome, namely Unigene10929 and Unigene7320. ‘Target’ indicates the chromosome located by those genes, its upper left indicates the chromosome serial number and genome versions, the upper right indicates the ID of query sequence and the number in brackets indicates the specific location of the gene on the chromosome. The gray bar marked as ‘No definition line’ is the query sequence, and the ‘HSP1,2,3...’ below it indicates the fragment of the gene matching the genome. Different colors represent different values of ‘Bit Scores’, the higher the ‘Bit Scores’ value, the greater the similarity.

**Supplementary Fig. S4. The gene structure analysis of RNase T2 family in *F. vesca* and *F. viridis*.**

The structure of all RNase T2 family genes in *F. vesca* and *F. viridis* is analyzed, and the abscissa indicates the sequence and structures size of different gene. The yellow squares represent exons, the green squares represent UTR, and the thick lines represent introns. In the *F. viridis*, only introns and exons are analyzed. The minimum and maximum of ORF lengths of RNase T2 family genes in *F. vesca* were 330 and 2751 bp, respectively, and contained 1-7 introns. The ORF lengths of the smallest and largest RNase T2 genes in *F. viridis* are 153 and 837 bp, respectively, and contain 1-6 introns.

**Supplementary Fig. S5. The nucleotide sequence alignment of *S_a_* and *S_b_*-RNase gene.**

The CDS sequence of S_a_ and S_b_-RNase transcripts (Unigene10929.1 and Unigene7320.1) were selected for comparison and analysis. ‘.’ in the picture indicates the gap, and the space indicates the nucleotide difference between S_a_ and S_b_-RNase, and ‘|’ means indicates the bases that the S_a_ and S_b_-RNase nucleotides are the same. The similarity of the two nucleotide sequences is 71.43%, the gap rate is 3.13%, and the conservative regions are shorter. The lengths of the nucleotide sequences of the S_a_ and S_b_-RNase genes are similar, only have a 9 bp difference, the CDS size is 657 bp and 666 bp, respectively. The black rectangle is the location of the degenerate primer.

**Supplementary Fig. S6. The location and number of introns in *S_a_* and *S_b_*-RNase.** The alignment results between two candidate genes (Unigene10929.1 and Unigene7320.1) and the spliced sequences are shown in this. Unigene10929.1 was divided into three segments by the 3 long fragments (scffold9638, scffold9635 and scffold9602) in Supplementary Dataset S4, contains two introns judge by GT-AG structural feature of intron’ both ends. Unigene7320.1 is divided into three parts by C625626, C633468 and scffold7146, also contain two introns judged by the same method. The two introns of Unigene10929.1 are located at 70-71 and 243-244 of the CDS sequence, the two introns of Unigene7320.1 are located at 70-71 and 240-241 of the CDS sequence, respectively. According to the largest predicted ORF (Supplementary Fig. S5), the full-length transcript sequences were divided into the CDS (Coding sequence) and UTR (Untranslated Regions). According to the GT---AG at both ends of the intron at the node, the CDSs were divided into intron and exon structure regions. The gray background shows the UTR sequence, the yellow background shows the CDS sequence, the green background shows part of the intron sequence, and the bases marked in red font are the intron GT---AG structure.

**Supplementary Fig. S7. The structure analysis of potential neighbouring lncRNAs1-6 of *S-RNase*.**

A indicate mapping of the reads to the DNA sequence matched by lncRNA1-2. The reads have 100% identity with the DNA sequence were used. There is no connected reads between LncRNA1 and LncRNA2, are from independent transcripts, respectively. In B, the CT-AC (GA-TG) structures is the boundary of introns.

**Supplementary Fig. S8. Collinearity analysis between *F. vesca* genome and rose genome, *Prunus* (almond) genome, and between two rose genomes.** A-1, B-1, C-1, D-1 represent the collinearity between the *F. vesca* genome and the rose genome 1 (Rosa_v1), the rose genome 2 (Rosa_v2), the *Prunus* (almond) genome, and between Rosa_v1 and Rosa_v2. Chr1(P)-8(P), Chr1(R1)-7(R1), Chr1(R2)-7(R2), Chr1-7 represent the genome chromosomes of almond, rose (Rosa_v1), rose (Rosa_v2), *F. vesca*, respectively, and collinearity was represented by lines of different thickness. A-2, B-2, C-2, and D-2 are collinearity represented by dot matrix view between different genomes, corresponding to A-1, B-1, C-1, and D-1 respectively.

**Supplementary Fig. S9. Collinearity analysis between the genome of the *F. vesca* and the chromosome located the S locus of almond and Rose.** The thick red line represents the pseudochromosome S locus of almond and rose located, and the thick green line represents the *F. vesca* genome. Here, remove the interference of some short collinearity regions, and reserve the larger areas with higher reliability to show collinearity. When the sequence of Pgl1 and AC8 (https://www.rosaceae.org/search/markers) from *P. dulcisis* blasted against the *F. vesca* genome, only one hit is obtained on chromosome 6 and 1, respectively.

**Supplementary Fig. S10. The S genotype identification of 29 selfing lines from *F. viridis* 42.** A, B and C represents the detection results of all cDNA samples with primers of *Sa*-RNase, *Sb*-RNase and *EF-1α* reference gene. The lanes 1-29 is the selfing progeny lines of *F. viridis* 42 (0-3 generations). The [serial](C:/Users/mi/AppData/Local/Yodao/DeskDict/frame/20181020195129/javascript:void(0);) [number](C:/Users/mi/AppData/Local/Yodao/DeskDict/frame/20181020195129/javascript:void(0);) and order (From left to right, top to bottom) of the selfing lines were shown as Table 2, and corresponding to the lanes 1-29, respectively.

**Supplementary Fig. S11. The differences of floral organs between compatible and incompatible states in *F. viridis*** (within 10 days after pollination).

**A. The differences in styles and the size of ovary in compatible and incompatible states.** The S genotypes of S0, S1-02-S2-49, and S1-02-S2-57 are S_a_S_b_, S_a_S_a_, and S_b_S_b_, respectively, see Table 1 for details in the main text. The pictures were taken at the same time on the 1st, 2nd, 3rd, 5th, 7th, 9th, and 11th day after pollination. A-1 means self-pollination, A-2 means S0 line without pollination after emasculation, A-3, A-4, A-5 means conducting **mutual pollination**^a^ between S0 and S1-02-S2-49, S0 and S1-02-S2-57, S1-02-S2-49 and S1-02-S2-57, respectively. On the third day after pollination, part of the stigmas appeared brown. On the fifth day after pollination, the style began to brown from the direction of the stigma, and completely browned on the seventh day. There was not obvious difference in the browning time between compatible and incompatible styles, but the unpollinated styles started to brown on the seventh day, and completely browned on the ninth day. It is obvious that the compatible ovary has been enlarged to a certain extent, and the incompatible ovary no change in size on the ninth day after pollination, and the difference is more obvious on the eleventh day. **B. The color of the ovary in the compatible and incompatible states.** From left to right indicate the ovary just before and after pollination, the compatible type ovary and the incompatible type ovary on the 11th day after pollination. The color marker strips from left to right is a gradual process, from yellow to yellow-green and to dark green, respectively. Just before and after pollination, the color of the ovary is similar to that of the style, is yellow. As time increases, the ovary gradually changes from yellow to green. The incompatible ovary becomes darker on the eleventh day after pollination, but the compatible ovary is yellow-green. This phenomenon can be explained by the fact that the ovary continues to grow after compatible pollination, but the incompatible ovary cannot continue to grow, leading to the accumulation of chlorophyll. **Mutual pollination^a^:** This means that two blooming flowers at the same time are smeared and pollinated with each other. Of course, during the smearing process, the pollen is mixed together, that is, the male parent is the pollen mixture of itself and the other line.

**Supplementary Fig. S12. The flowers of *F. viridis*. A-E. The different growth states.** A is the small bud stage, B is the medium bud stage, C is the big bud stage, D is the opening flower stage, and E is the blooming flower stage. Wang et al.^7^ measured the pollen vigor of different growth states and found that the pollen vigor is very different in different growth states of flowers. Generally, the pollen vitality of the flowers that have just opened and bloomed soon is the highest, which can truly reflect the pollen vitality in the normal state, but because the petals are opened, it may cause the pollution of external pollen. Generally, it shows that the pollen vitality of the flowers that are just opening and not long after blooming is the highest, which can truly reflect the pollen vitality in the normal state, but because the petals are opened, it may cause the pollution of external pollen. The petals of the flowers in the bud stage are not opened, and the pollen maintain high vitality, it is generally selected for isolation pollination experiments. **F. The split view of flower.** From the inside to the outside are the receptacle, ovary, style, filament, anther, petal, calyx, and pedicel. The ovary is born on the receptacle, and the style is born on the ovary, style, ovary and receptacle are born close together as globular tissue, which is defined as flower ball in this article. For the convenience of sampling, we take the flower ball containing the style as a whole to obtain the style transcriptome data and proteome data.

**Supplementary Fig. S13. The S locus and S-RNase analysis of rose genome.** The candidate S locus of rose is located on chromosome 3 in genome Rosa_v1 (*Rosa chinensis* genome v1.0), and there is a candidate S-RNase at this locus^8^. According to the collinearity between the two roses, we further found a locus on Rosa_v2 (*Rosa chinensis* ‘Old Blush’ homozygous genome v2.0), which is located on chromosome 3 that has a collinearity with S locus of the Rosa_v1. There is a candidate S-RNase (RcHm_v2.0_Chr3g0455911) in the locus, but the gene lacks a signal peptide which may be caused by incomplete transcripts during genome annotation. According to the longer transcript XM_024334248.1 in NCBI, the sequence structure of S_R_-RNase of genome Rosa_v2 was re-annotated. S_R_-RNase has high homology with the two S-RNases of *F. viridis* verified in this paper, and the relationship is close (A). The S_R_-RNase, S_a_-RNase and S_b_-RNase genes with the red background are all clustered together and have relatively similar relationship with the S-RNase gene of the the Amygdaleae marked by the green background. The blue area represents the S-RNase gene of the the Maleae, the purple-labeled S-RNase gene are from Solanaceae. Similar to S_a_-RNase and S_b_-RNase, it has five conserved structures of C1-C3, RC4, C5 and hypervariable regions (B), and conforms to the characteristics of S-RNase (C). The S locus information of the two-rose genome provides a theoretical basis for further utilization.

**Supplementary Table S1. Amino acid similarity analysis related to RNase T2 family members of *F. vesca* and *F. viridis***

**Note:** The seven genes of Unigene18150.1, Unigene13465.1, Unigene11523.1, CL6424.Contig1.1, CL6424.Contig1.1, Unigene10929.1 and Unigene7320.1 are the RNase T2 family members containing above 60% RNase T2 domain from *F. viridis*; The ten genes of FvH4_4g31300.1, FvH4_4g31300.1, FvH4_4g31290.1, FvH4_2g17310.1, FvH4_5g24800.1, FvH4_1g10040.1, FvH4_6g07690.1, FvH4_5g33850.1, FvH4_1g19170.1 and FvH4_4g18130.1 are genes above 60% RNase T2 domain from *F. vesca*; BAH22124.1 and BAA95317.1 are from the genus *Prunus*; AWL24810.1 and ADB85482.1 are from the genus *Malus*; BAN66319.1 and ADB91384.1 are from the genus *Pyrus*; BAC00940.1 and CAA53666.1 come from the Solanaceae. The V6 version of DNAMAN software is used for amino acid sequence similarity analysis. The similarity is expressed as a percentage and two decimal places are retained.

**Supplementary Table S2. The S-RNase for analysis of RNase T2 family members from *F. vesca* and *F. viridis***

**Supplementary Table S3. The primer list**

**Supplementary Table S4. The *cis*-acting elements in the promoter sequence of *S_a_*-RNase. Note:** According to online analysis of PlantCARE^9^, this promoter contains the core *cis*-acting elements of typical eukaryotic promoters, such as TATA box, CAAT-box that control the frequency of transcription initiation. Some elements are related to plant hormone response, such as jasmonic acid-induced CGTCA-motif, auxin-induced TGA-element, gibberellin response GARE-motif, salicylic acid-related element TCA-element. In addition, there are also some *cis*-acting elements that have not yet been characterized.

**Supplementary Table S5. The *cis*-acting elements in the promoter sequence of lncRNA5**

**Supplementary Table S6. The (in)compatibility analysis according to the seed and fruit setting rate after intraspecific pollination combinations of different S genotype lines of *F. viridis***

**Supplementary Table S7. The (in)compatibility analysis according to the seed and fruit setting rate after interspecific cross pollination.**

**Supplementary Sequence S1. The DNA reference sequences of *S_a_*-RNase.**

**Supplementary Sequence S2. The DNA reference sequences of *S_b_*-RNase.**


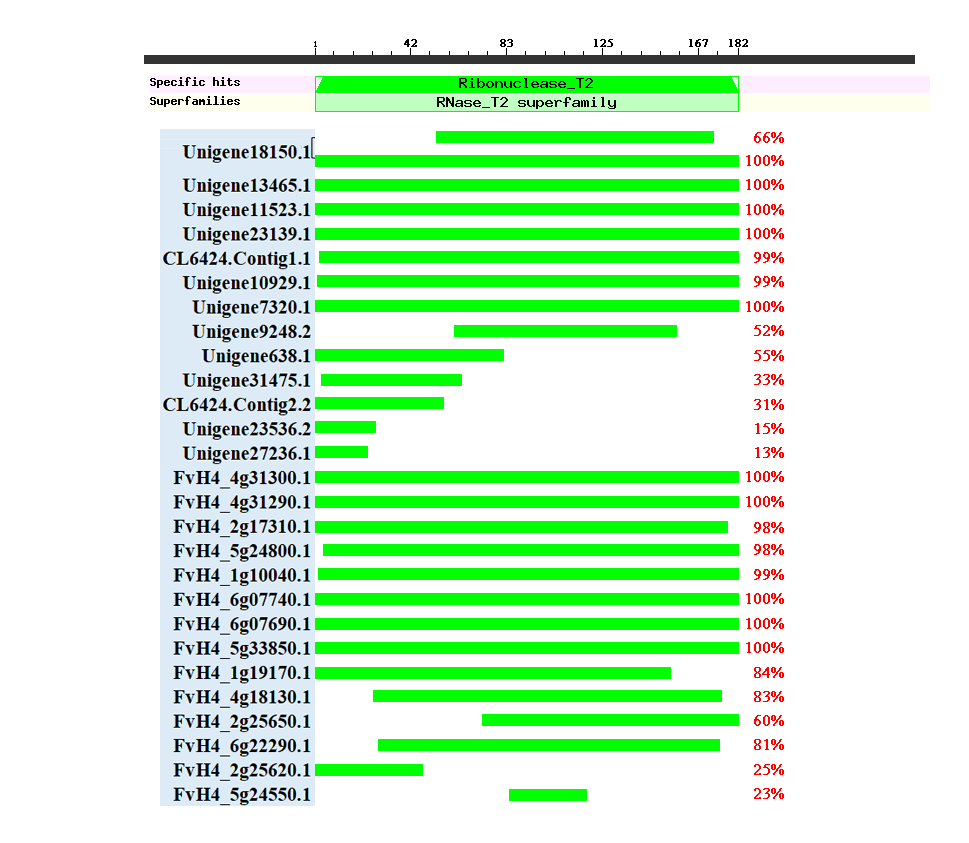


**Fig. S1. The size and approximate location of the domains contained in RNase T2 family genes of *F. vesca* and *F. viridis*.**

All genes selected from the *F. vesca* and *F. viridis* databases are used to analyze the integrity of the RNase T2 domain. The left side is the gene name, the right side is the size of the domain, the length of the green band indicates the size of the structure, and the two ends indicate the start and end of the domain, respectively. The top ruler is represented by the number of amino acids, and the bar closest to the ruler represents the RNase T2 reference model. The complete RNase T2 domain is 182 amino acids in size.

**Fig. S2. Genome-wide chromosomal mapping of RNase T2 and F-box family genes in *F. vesca*.**

The number on the left represents the specific position of the gene on the chromosome, the black font on the right represents the F-box family gene, the red font represents the RNase T2 family gene, and the bottom notes is the chromosome number. The gene location information is obtained from the *F. vesca* database GFF file, the location of genes on the chromosomes is graphically using Mapchart. There are 2 RNase T2 family genes distributed on chromosome 1, and 3 RNase T2 family genes on each chromosome 2, 4, 5 and 6, and 61, 70, 87, 51, 47, 114, 41 F-box genes on chromosome 1, 2, 3, 4, 5, 6 and 7 (selected by F-box Markov model only), respectively. S-RNase and SFB/SFBB/SLF genes are often located within a range on the S locus, but the distance between both genes has greatly difference depending on the S genotype. The physical distance of the S haplotype of Solanaceae is significantly larger than that of Rosaceae, and the physical range of the S haplotype of Solanaceae is about 4 Mb^1^. The physical distances between different species of Rosaceae are not the same, both *Malus* and *Pyrus* are located on chromosome 17, and the size is about 1Mb and 649Kb respectively^2^. In almonds (*P. dulcis*), the range is within 70 Kb^3^ and the SFB alleles within 30 Kb^4^, and in sweet cherries (*P. avium*), from 380 bp to 40 Kb^5,6^. Therefore, we used the larger distance range of Rosaceae as a reference to analyze the existence of F-box gene flanking the RNase T2 gene. There is no F-box gene within the 500 Kb flanks of FvH4_2g25650.1, FvH4_2g25620.1, FvH4_4g18130.1, FvH4_5g24550.1, FvH4_5g33850.1 and FvH4_5g24800.1, and no F-box gene within 380 Kb flanks of FvH4_2g17310.1 and FvH4_6g22290.1. FvH4_1g19170.1, FvH4_1g10040.1, FvH4_4g31300.1, FvH4_4g31290.1, FvH4_6g07740.1 and FvH4_6g07690.1 have F-box genes in the close physical distances.


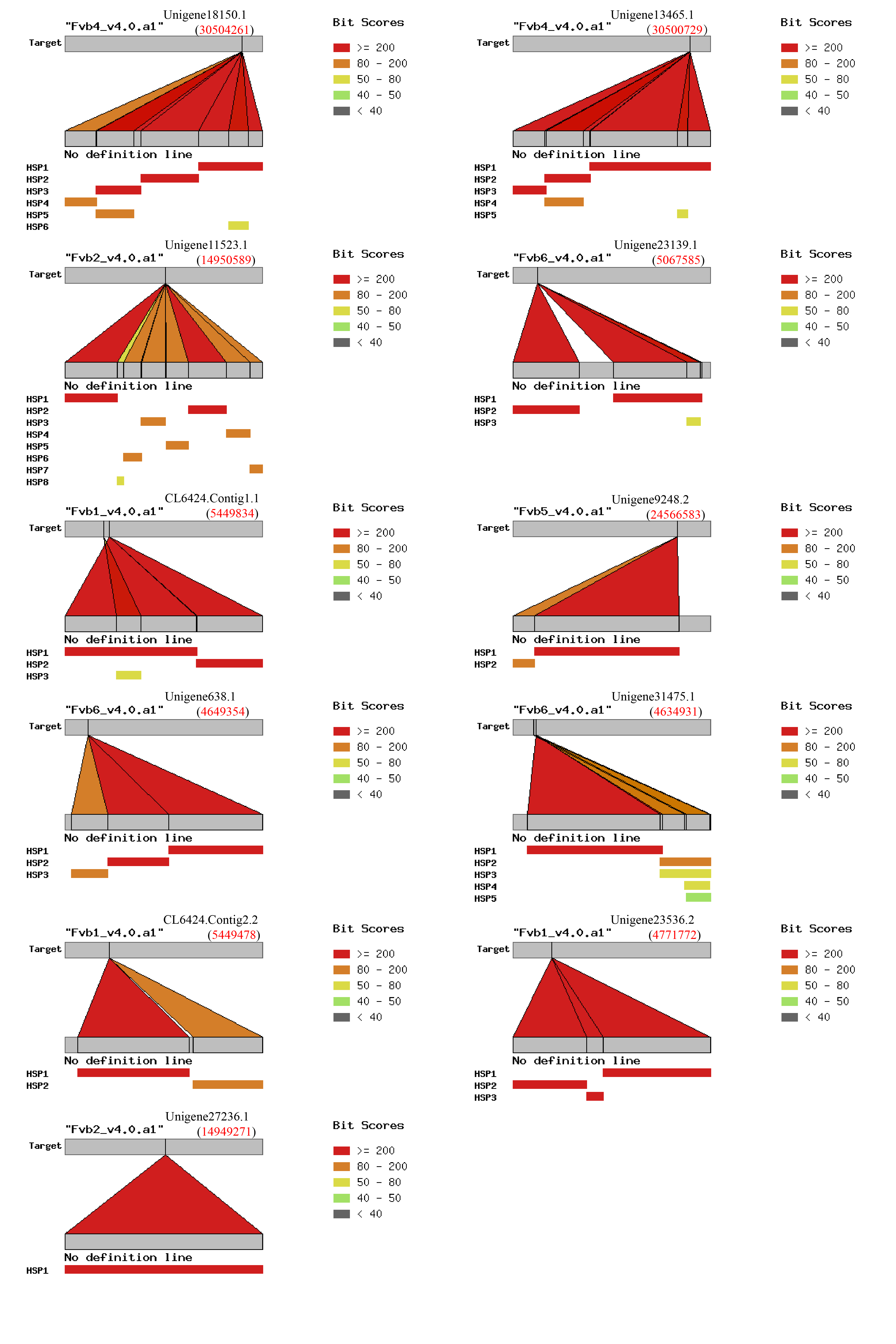


**Fig. S3. Chromosome location analysis of RNase T2 family genes in *F. viridis*.**

Blastn tool (https://www.rosaceae.org/blast/nucleotide/nucleotide) was used to perform gene mapping analysis of RNase T2 family members obtained from *F. viridis* taking the *F. vesca* genome_v4.0.a1 as the reference genome. Among the RNase T2 family genes of *F. viridis*, 11 genes have been assigned to the reference genome, and two genes have not been mapped to the genome, namely Unigene10929 and Unigene7320. ‘Target’ indicates the chromosome located by those genes, its upper left indicates the chromosome serial number and genome versions, the upper right indicates the ID of query sequence and the number in brackets indicates the specific location of the gene on the chromosome. The gray bar marked as ‘No definition line’ is the query sequence, and the ‘HSP1,2,3...’ below it indicates the fragment of the gene matching the genome. Different colors represent different values of ‘Bit Scores’, the higher the ‘Bit Scores’ value, the greater the similarity.


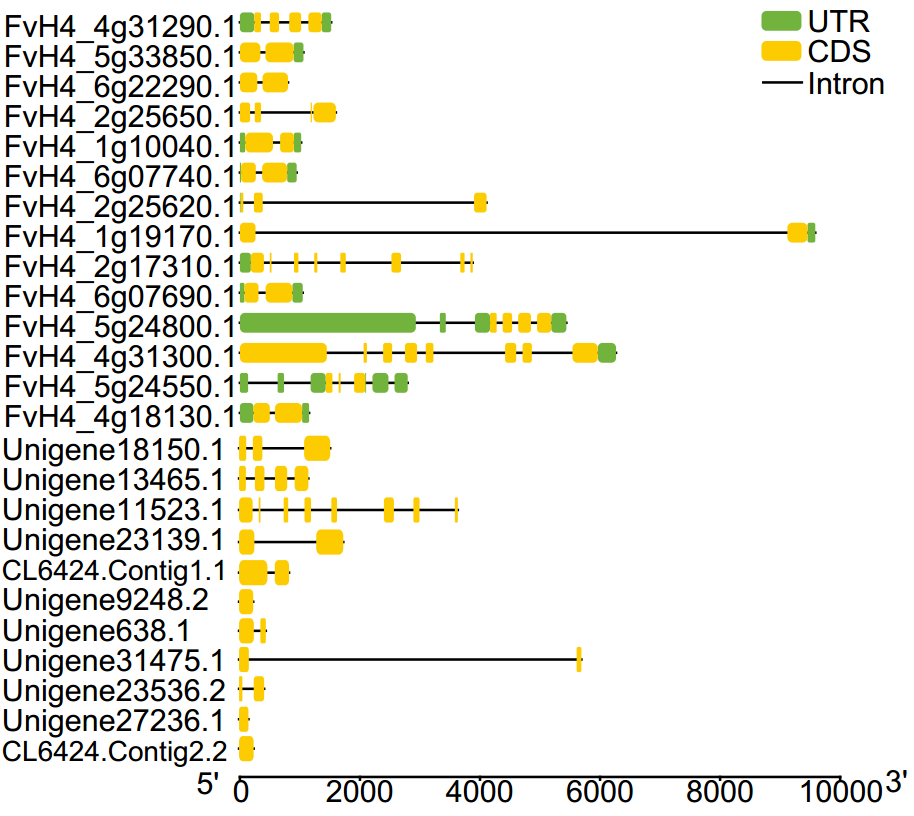


**Fig. S4. The gene structure analysis of RNase T2 family in *F. vesca* and *F. viridis*.**

The structure of all RNase T2 family genes in *F. vesca* and *F. viridis* is analyzed, and the abscissa indicates the sequence and structures size of different gene. The yellow squares represent exons, the green squares represent UTR, and the thick lines represent introns. In the *F. viridis*, only introns and exons are analyzed. The minimum and maximum of ORF lengths of RNase T2 family genes in *F. vesca* were 330 and 2751 bp, respectively, and contained 1-7 introns. The ORF lengths of the smallest and largest RNase T2 genes in *F. viridis* are 153 and 837 bp, respectively, and contain 1-6 introns.

**
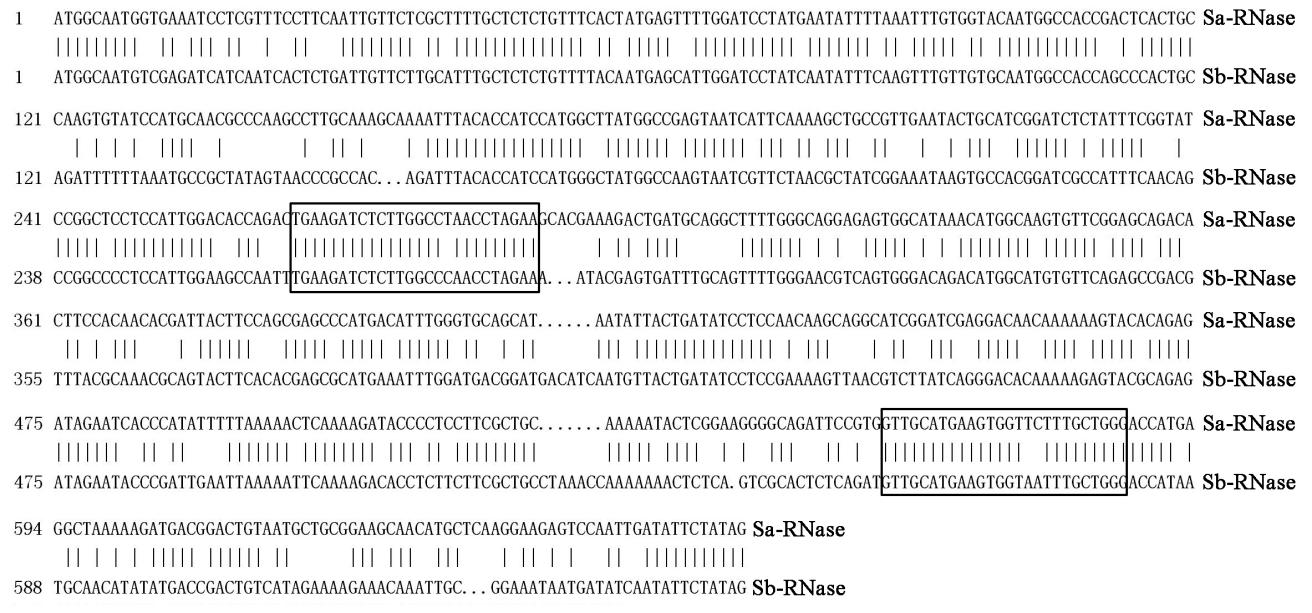
**

**Fig. S5. The nucleotide sequence alignment of *S_a_* and *S_b_*-RNase gene.**

The CDS sequence of S_a_ and S_b_-RNase transcripts (Unigene10929.1 and Unigene7320.1) were selected for comparison and analysis. ‘.’ in the picture indicates the gap, and the space indicates the nucleotide difference between S_a_ and S_b_-RNase, and ‘|’ means indicates the bases that the S_a_ and S_b_-RNase nucleotides are the same. The similarity of the two nucleotide sequences is 71.43%, the gap rate is 3.13%, and the conservative regions are shorter. The lengths of the nucleotide sequences of the S_a_ and S_b_-RNase genes are similar, only have a 9 bp difference, the CDS size is 657 bp and 666 bp, respectively. The black rectangle is the location of the degenerate primer.

**
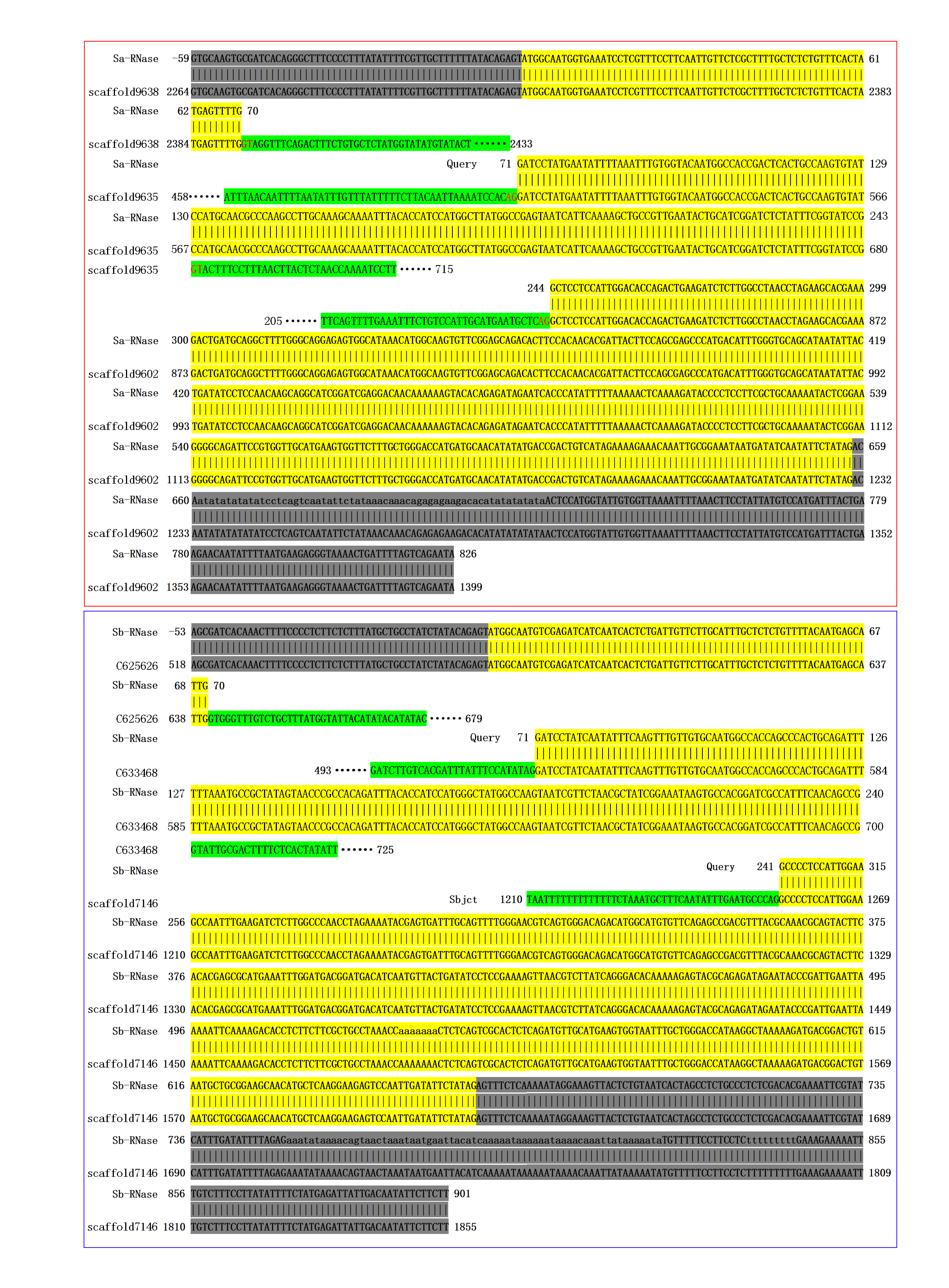
**

**Fig. S6. The location and number of introns in *S_a_* and *S_b_*-RNase.**

The alignment results between two candidate genes (Unigene10929.1 and Unigene7320.1) and the spliced sequences are shown in this. Unigene10929.1 was divided into three segments by the 3 long fragments (scffold9638, scffold9635 and scffold9602) in Supplementary Dataset S4, contains two introns judge by GT-AG structural feature of intron’ both ends. Unigene7320.1 is divided into three parts by C625626, C633468 and scffold7146, also contain two introns judged by the same method. The two introns of Unigene10929.1 are located at 70-71 and 243-244 of the CDS sequence, the two introns of Unigene7320.1 are located at 70-71 and 240-241 of the CDS sequence, respectively. According to the largest predicted ORF (Supplementary Fig. S5), the full-length transcript sequences were divided into the CDS (Coding sequence) and UTR (Untranslated Regions). According to the GT---AG at both ends of the intron at the node, the CDSs were divided into intron and exon structure regions. The gray background shows the UTR sequence, the yellow background shows the CDS sequence, the green background shows part of the intron sequence, and the bases marked in red font are the intron GT---AG structure.


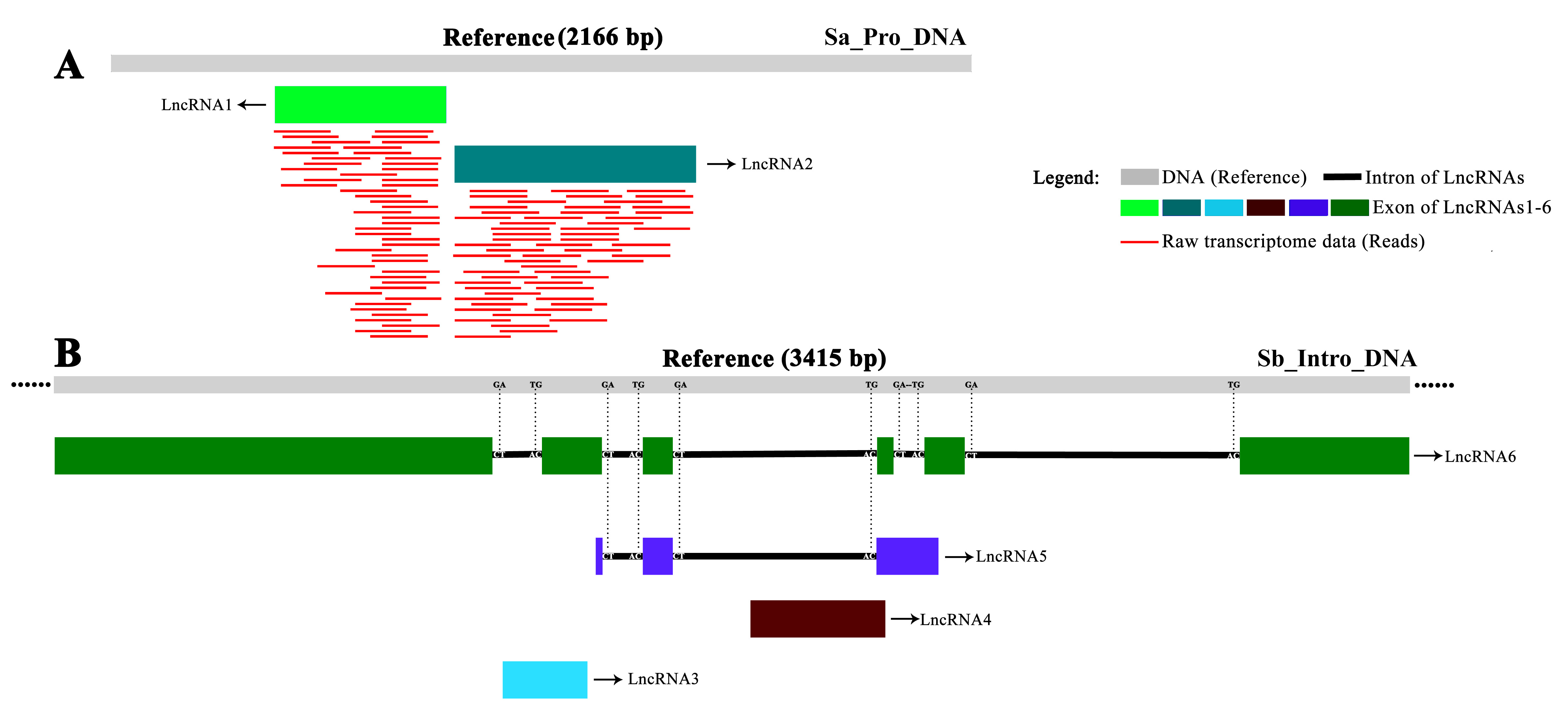


**Fig. S7. The structure analysis of potential neighbouring lncRNAs1-6 of *S-RNase*.**

A indicate mapping of the reads to the DNA sequence matched by lncRNA1-2. The reads have 100% identity with the DNA sequence were used. There is no connected reads between LncRNA1 and LncRNA2, are from independent transcripts, respectively. In B, the CT-AC (GA-TG) structures is the boundary of introns.





**Fig. S8. Collinearity analysis between *F. vesca* genome and rose genome, *Prunus* (amonld) genome, and between two rose genomes.**

A-1, B-1, C-1, D-1 represent the collinearity between the *F. vesca* genome and the rose genome 1 (Rosa_v1), the rose genome 2 (Rosa_v2), the *Prunus* (almond) genome, and between Rosa_v1 and Rosa_v2. Chr1(P)-8(P), Chr1(R1)-7(R1), Chr1(R2)-7(R2), Chr1-7 represent the genome chromosomes of almond, rose (Rosa_v1), rose (Rosa_v2), *F. vesca*, respectively, and collinearity was represented by lines of different thickness. A-2, B-2, C-2, and D-2 are collinearity represented by dot matrix view between different genomes, corresponding to A-1, B-1, C-1, and D-1 respectively.


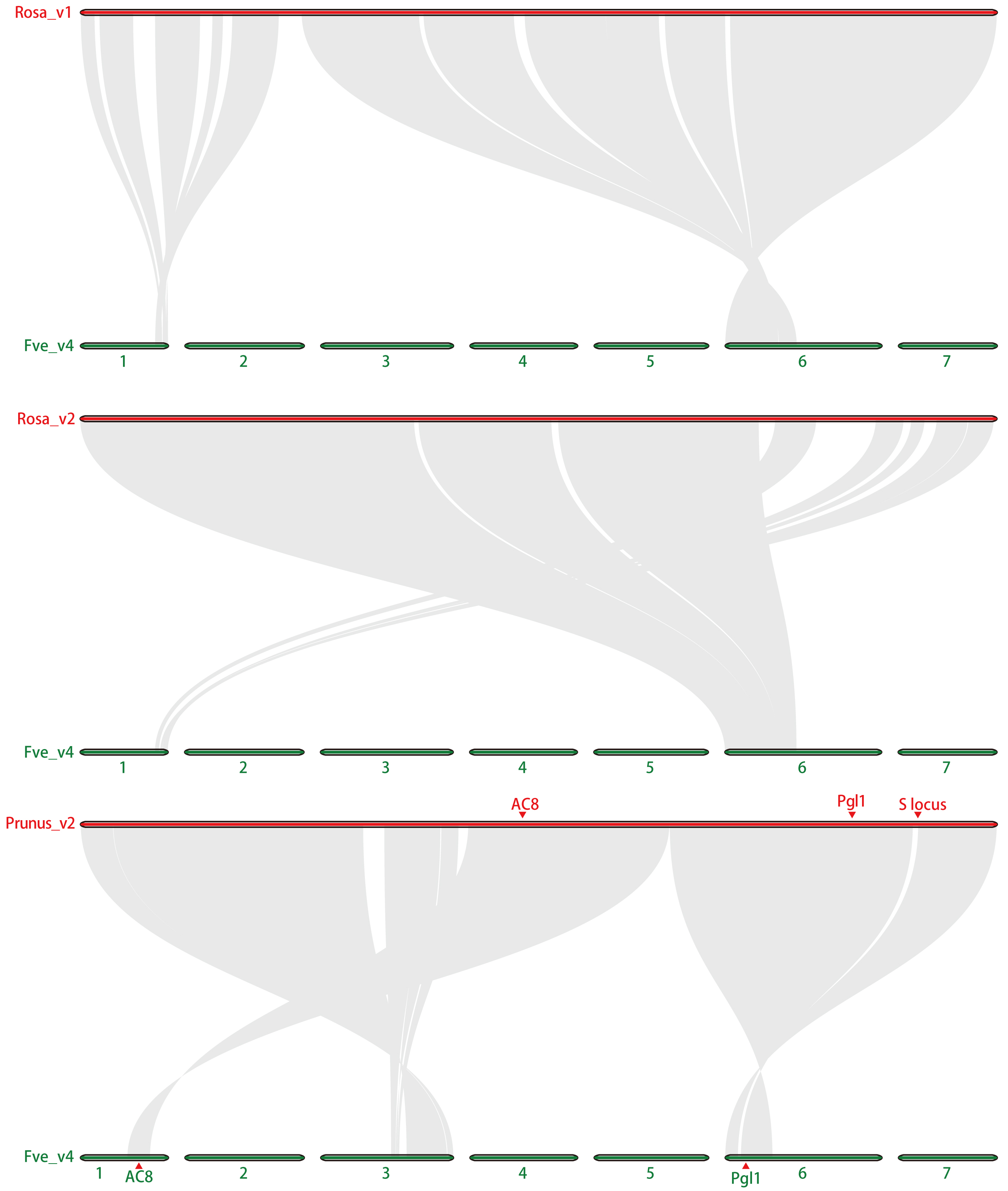


**Fig. S9. Collinearity analysis between the genome of the *F. vesca* and the chromosome located the S locus of almond and Rose.**

The thick red line represents the pseudochromosome S locus of almond and Rose located, and the thick green line represents the *F. vesca* genome. Here, remove the interference of some short collinearity regions, and reserve the larger areas with higher reliability to show collinearity. When the sequence of Pgl1 and AC8 (https://www.rosaceae.org/search/markers) from *P. dulcis is* blasted against the *F. vesca* genome, only one hit is obtained on chromosome 6 and 1, respectively.


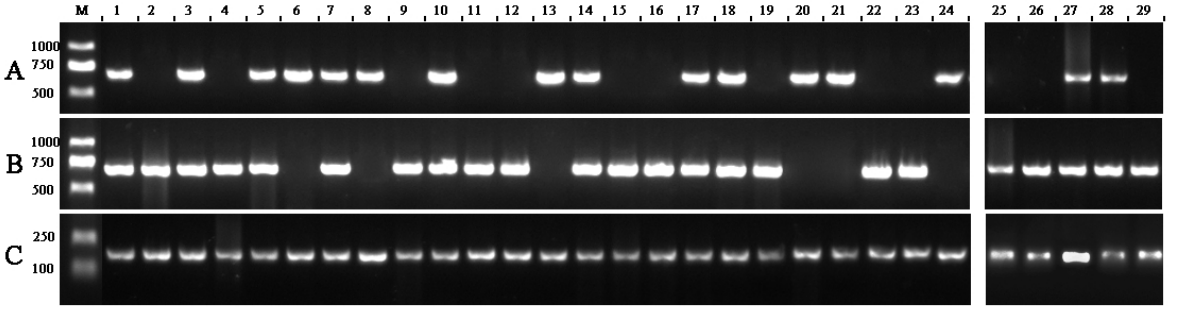


**Fig. S10. The S genotype identification of 29 selfing lines from *F. viridis* 42.**

A, B and C represents the detection results of all cDNA samples with primers of *Sa*-RNase, *Sb*-RNase and *EF-1α* reference gene. The lanes 1-29 is the selfing progeny lines of *F. viridis* 42 (0-3 generations). The [serial](C:/Users/mi/AppData/Local/Yodao/DeskDict/frame/20181020195129/javascript:void(0);) [number](C:/Users/mi/AppData/Local/Yodao/DeskDict/frame/20181020195129/javascript:void(0);) and order (From left to right, top to bottom) of the selfing lines were shown as Table 2, and corresponding to the lanes 1-29, respectively.





**Supplementary Fig. S11. The differences of floral organs between compatible and incompatible states in *F. viridis*** (within 10 days after pollination). **A. The differences in styles and the size of ovary in compatible and incompatible states.** The S genotypes of S0, S1-02-S2-49, and S1-02-S2-57 are S_a_S_b_, S_a_S_a_, and S_b_S_b_, respectively, see Table 1 for details in the main text. The pictures were taken at the same time on the 1st, 2nd, 3rd, 5th, 7th, 9th, and 11th day after pollination. A-1 means self-pollination, A-2 means S0 line without pollination after emasculation, A-3, A-4, A-5 means conducting **mutual pollination** between S0 and S1-02-S2-49, S0 and S1-02-S2-57, S1-02-S2-49 and S1-02-S2-57, respectively. On the third day after pollination, part of the stigmas appeared brown. On the fifth day after pollination, the style began to brown from the direction of the stigma, and completely browned on the seventh day. There was not obvious difference in the browning time between compatible and incompatible styles, but the unpollinated styles started to brown on the seventh day, and completely browned on the ninth day. It is obvious that the compatible ovary has been enlarged to a certain extent, and the incompatible ovary no change in size on the ninth day after pollination, and the difference is more obvious on the eleventh day. **B. The color of the ovary in the compatible and incompatible states.** From left to right indicate the ovary just before and after pollination, the compatible type ovary and the incompatible type ovary on the 11th day after pollination. The color marker strips from left to right is a gradual process, from yellow to yellow-green and to dark green, respectively. Just before and after pollination, the color of the ovary is similar to that of the style, is yellow. As time increases, the ovary gradually changes from yellow to green. The incompatible ovary becomes darker on the eleventh day after pollination, but the compatible ovary is yellow-green. This phenomenon can be explained by the fact that the ovary continues to grow after compatible pollination, but the incompatible ovary cannot continue to grow, leading to the accumulation of chlorophyll. **Mutual pollination:** This means that two blooming flowers at the same time are smeared and pollinated with each other. Of course, during the smearing process, the pollen is mixed together, that is, the male parent is the pollen mixture of itself and the other line.





**Fig. S12. The flowers of *F. viridis*.**

**A-E. The different growth states.** A is the small bud stage, B is the medium bud stage, C is the big bud stage, D is the opening flower stage, and E is the blooming flower stage. Wang et al.^7^ measured the pollen vigor of different growth states and found that the pollen vigor is very different in different growth states of flowers. Generally, the pollen vitality of the flowers that have just opened and bloomed soon is the highest, which can truly reflect the pollen vitality in the normal state, but because the petals are opened, it may cause the pollution of external pollen. Generally, it shows that the pollen vitality of the flowers that are just opening and not long after blooming is the highest, which can truly reflect the pollen vitality in the normal state, but because the petals are opened, it may cause the pollution of external pollen. The petals of the flowers in the bud stage are not opened, and the pollen maintain high vitality, it is generally selected for isolation pollination experiments. **F. The split view of flower.** From the inside to the outside are the receptacle, ovary, style, filament, anther, petal, calyx, and pedicel. The ovary is born on the receptacle, and the style is born on the ovary, style, ovary and receptacle are born close together as globular tissue, which is defined as flower ball in this article. For the convenience of sampling, we take the flower ball containing the style as a whole to obtain the style transcriptome data and proteome data.


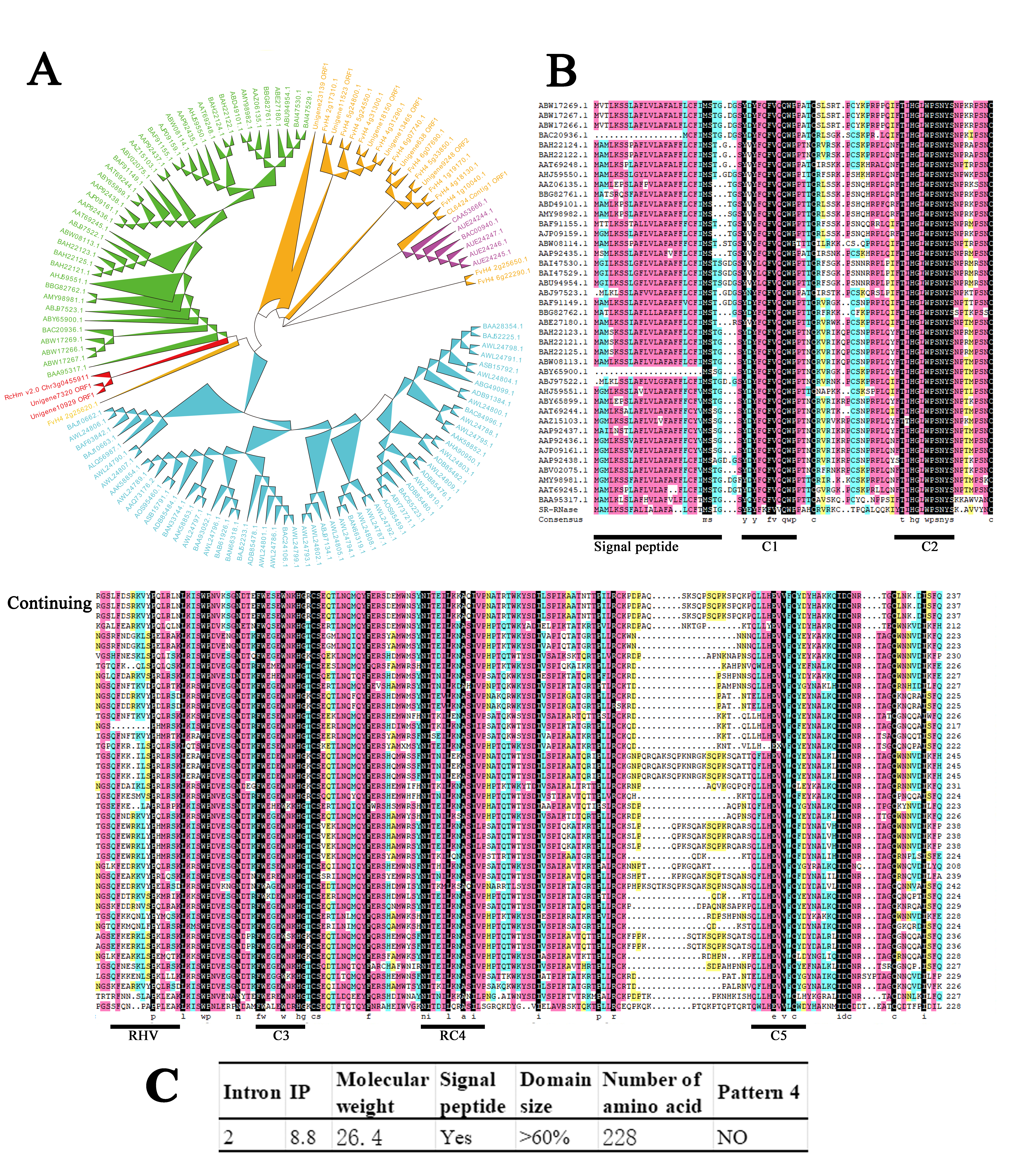


**Fig. S13. The S locus and S-RNase analysis of rose genome.**

The candidate S locus of rose is located on chromosome 3 in genome Rosa_v1 (*Rosa chinensis* genome v1.0), and there is a candidate S-RNase at this locus^8^. According to the collinearity between the two roses, we further found a locus on Rosa_v2 (*Rosa chinensis* ‘Old Blush’ homozygous genome v2.0), which is located on chromosome 3 that has a collinearity with S locus of the Rosa_v1. There is a candidate SR-RNase (RcHm_v2.0_Chr3g0455911) in the locus, but the gene lacks a signal peptide. According to the XM_024334248.1 in NCBI, the sequence structure of SR-RNase of genome Rosa_v2 was re-annotated. S_R_-RNase has high homology with the two S-RNases of *F. viridis* verified in this paper, and the relationship is close (A). The S_R_-RNase, S_a_-RNase and S_b_-RNase genes with the red background are all clustered together and have relatively similar relationship with the S-RNase gene of the the Amygdaleae marked by the green background. The blue area represents the S-RNase gene of the the Maleae, the purple-labeled S-RNase gene are from Solanaceae. Similar to S_a_-RNase and S_b_-RNase, it has five conserved structures of C1-C3, RC4, C5 and hypervariable regions (B), and conforms to the characteristics of S-RNase (C). The S locus information of the two-rose genome provides a theoretical basis for further utilization.

**Supplementary Table S1. Amino acid similarity analysis related to RNase T2 family members of *F. vesca* and *F. viridis***

|  | **Unigene18150.1** | **Unigene13465.1** | **Unigene11523.1** | **Unigene23139.1** | **CL6424.Contig1.1** | **Unigene10929.1** | **Unigene7320.1** | **BAH22124.1** | **BAA95317.1** | **AWL24810.1** | **ADB85482.1** | **BAN66319.1** | **ADB91384.1** | **BAC00940.1** | **CAA53666.1** |
| --- | --- | --- | --- | --- | --- | --- | --- | --- | --- | --- | --- | --- | --- | --- | --- |
| **Unigene18150.1** |  | **62.25** | **23.71** | **20.00** | **18.54** | **22.49** | **15.60** | **25.67** | **26.36** | **19.73** | **20.18** | **20.36** | **25.24** | **20.59** | **25.25** |
| **Unigene13465.1** |  |  | **29.39** | **25.37** | **17.86** | **18.66** | **20.62** | **25.95** | **24.66** | **17.86** | **19.20** | **17.98** | **20.26** | **16.49** | **24.54** |
| **Unigene11523.1** |  |  |  | **18.42** | **19.25** | **13.48** | **12.30** | **21.56** | **21.50** | **19.18** | **19.11** | **19.03** | **21.65** | **16.41** | **23.96** |
| **Unigene23139.1** |  |  |  |  | **15.00** | **26.64** | **30.88** | **32.29** | **33.18** | **20.91** | **20.45** | **18.14** | **21.78** | **17.29** | **30.09** |
| **CL6424.Contig1.1** |  |  |  |  |  | **21.35** | **17.92** | **14.29** | **23.95** | **17.09** | **15.76** | **20.00** | **6.92** | **22.60** | **20.10** |
| **Unigene10929.1** |  |  |  |  |  |  | **56.48** | **46.51** | **47.22** | **25.49** | **20.18** | **28.50** | **22.48** | **19.05** | **20.11** |
| **Unigene7320.1** |  |  |  |  |  |  |  | **42.59** | **44.04** | **20.23** | **20.93** | **20.18** | **25.11** | **14.49** | **21.28** |

|  | **FvH4_4g31300.1** | **FvH4_4g31290.1** | **FvH4_2g17310.1** | **FvH4_5g24800.1** | **FvH4_1g10040.1** | **FvH4_6g07740.1** | **FvH4_6g07690.1** | **FvH4_5g33850.1** | **FvH4_1g19170.1** | **FvH4_4g18130.1** | **Unigene10929.1** | **Unigene7320.1** | **BAH22124.1** | **BAA95317.1** | **AWL24810.1** | **ADB85482.1** | **BAN66319.1** | **ADB91384.1** | **BAC00940.1** | **CAA53666.1** |
| --- | --- | --- | --- | --- | --- | --- | --- | --- | --- | --- | --- | --- | --- | --- | --- | --- | --- | --- | --- | --- |
| **FvH4_4g31300.1** |  | **63.76** | **21.78** | **34.93** | **14.85** | **34.03** | **18.39** | **22.32** | **16.06** | **10.79** | **18.66** | **15.60** | **20.08** | **25.91** | **17.49** | **18.83** | **16.14** | **24.34** | **20.10** | **25.76** |
| **FvH4_4g31290.1** |  |  | **28.84** | **38.43** | **16.97** | **21.46** | **19.47** | **15.23** | **28.05** | **12.60** | **18.66** | **18.04** | **25.95** | **24.66** | **17.86** | **19.20** | **17.98** | **20.26** | **17.01** | **24.54** |
| **FvH4_2g17310.1** |  |  |  | **28.57** | **18.71** | **18.45** | **20.10** | **22.73** | **22.22** | **17.06** | **11.38** | **13.77** | **20.00** | **24.30** | **14.22** | **18.03** | **15.49** | **24.03** | **12.99** | **22.84** |
| **FvH4_5g24800.1** |  |  |  |  | **12.86** | **15.71** | **12,50** | **10.98** | **11.11** | **10.53** | **19.35** | **13.57** | **12.56** | **16.30** | **21.52** | **10.87** | **20.00** | **20.00** | **11.79** | **13.55** |
| **FvH4_1g10040.1** |  |  |  |  |  | **17.43** | **16.75** | **21.90** | **13.54** | **14.84** | **17.96** | **16.76** | **15.23** | **23.95** | **17.37** | **18.59** | **19.21** | **6.87** | **20.90** | **21.76** |
| **FvH4_6g07740.1** |  |  |  |  |  |  | **30.09** | **23.41** | **21.65** | **24.04** | **21.66** | **19.53** | **27.73** | **27.98** | **18.72** | **17.81** | **21.10** | **16.89** | **22.75** | **24.61** |
| **FvH4_6g07690.1** |  |  |  |  |  |  |  | **24.55** | **22.09** | **12.74** | **28.37** | **26.15** | **31.34** | **31.65** | **21.82** | **21.82** | **27.04** | **22.65** | **21.13** | **28.37** |
| **FvH4_5g33850.1** |  |  |  |  |  |  |  |  | **28.42** | **17.55** | **22.43** | **34.88** | **17.49** | **29.31** | **14.39** | **16.06** | **15.33** | **14.81** | **9.80** | **22.86** |
| **FvH4_1g19170.1** |  |  |  |  |  |  |  |  |  | **14.14** | **19.05** | **22.70** | **28.80** | **12.85** | **20.34** | **19.37** | **20.94** | **20.21** | **4.84** | **17.03** |
| **FvH4_4g18130.1** |  |  |  |  |  |  |  |  |  |  | **18.37** | **10.38** | **9.68** | **14.63** | **10.50** | **12.06** | **11.63** | **15.29** | **10.00** | **25.66** |

**Note:** The seven genes of Unigene18150.1, Unigene13465.1, Unigene11523.1, CL6424.Contig1.1, CL6424.Contig1.1, Unigene10929.1 and Unigene7320.1 are the RNase T2 family members containing above 60% RNase T2 domain from *F. viridis*; The ten genes of FvH4_4g31300.1, FvH4_4g31300.1, FvH4_4g31290.1, FvH4_2g17310.1, FvH4_5g24800.1, FvH4_1g10040.1, FvH4_6g07690.1, FvH4_5g33850.1, FvH4_1g19170.1 and FvH4_4g18130.1 are genes above 60% RNase T2 domain from *F. vesca*; BAH22124.1 and BAA95317.1 are from the genus *Prunus*; AWL24810.1 and ADB85482.1 are from the genus *Malus*; BAN66319.1 and ADB91384.1 are from the genus *Pyrus*; BAC00940.1 and CAA53666.1 come from the Solanaceae. The V6 version of DNAMAN software is used for amino acid sequence similarity analysis. The similarity is expressed as a percentage and two decimal places are retained.

**Supplementary Table S2. The S-RNase for analysis of RNase T2 family members from *F. vesca* and *F. viridis***

| **Gene ID** | **Gene name** | **Gene Source** |
| --- | --- | --- |
| BAH22124.1 | S-RNase | *Prunus yedoensis* |
| BAH22123.1 | S-RNase | *Prunus yedoensis* |
| BAH22122.1 | S-RNase | *Prunus yedoensis* |
| BAH22121.1 | S-RNase | *Prunus yedoensis* |
| ABY65900.1 | S-RNase | *Prunus pseudocerasus* |
| ABY65899.1 | S-RNase | *Prunus pseudocerasus* |
| AAZ06135.1 | S-RNase | *Prunus dulcis* |
| AAZ15103.1 | S-RNase | *Prunus dulcis* |
| BAH22125.1 | S-RNase | *Prunus subhirtella* var. *ascendens* |
| ABU94954.1 | S-RNase | *Prunus dulcis* |
| ABJ97522.1 | S-RNase | *Prunus webbii* |
| ABJ97523.1 | S-RNase | *Prunus webbii* |
| AAP92438.1 | S-RNase | *Prunus avium* |
| AAP92437.1 | S-RNase | *Prunus avium* |
| AAP92436.1 | S-RNase | *Prunus avium* |
| AAP92435.1 | S-RNase | *Prunus avium* |
| AHJ59550.1 | S locus S-RNase 52 | *Prunus armeniaca* |
| AHJ59551.1 | S-RNase 53 | *Prunus armeniaca* |
| ABE27180.1 | S-locus S-RNase c | *Prunus armeniaca* |
| BAF91155.1 | S-ribonuclease | *Prunus mume* |
| BAF91149.1 | S-ribonuclease | *Prunus mume* |
| ABW08114.1 | S26-RNase | *Prunus cerasus* |
| ABW08113.1 | S7-RNase | *Prunus avium* |
| ABW17269.1 | S36b3-RNase | *Prunus cerasus* |
| ABW17267.1 | S36b-RNase | *Prunus cerasus* |
| ABW17266.1 | S36a-RNase | *Prunus cerasus* |
| ABD49101.1 | S13-RNase | *Prunus avium* |
| BAI47530.1 | Sf-RNase | *Prunus dulcis* |
| BAI47529.1 | S30-RNase | *Prunus dulcis* |
| ABV02075.1 | S-locus S-RNase S16 | *Prunus spinosa* |
| AJP09161.1 | S14 S-RNase | *Prunus tenella* |
| AJP09159.1 | S12 S-RNase | *Prunus tenella* |
| AMY98982.1 | S17-RNase protein | *Prunus tenella* |
| AMY98981.1 | S16-RNase protein | *Prunus tenella* |
| BAA95317.1 | S_a_-RNase | *Prunus dulcis* |
| BBG82762.1 | S31-RNase | *Prunus armeniaca* |
| BBG82761.1 | S30-RNase | *Prunus armeniaca* |
| AAT69248.1 | S4-RNase protein | *Prunus armeniaca* |
| AAT69245.1 | S2-RNase protein | *Prunus armeniaca* |
| AAT69244.1 | S1-RNase protein | *Prunus armeniaca* |
| BAC20936.1 | Sc-RNase | *Prunus salicina* |
| AWL24810.1 | S-RNase | *Malus domestica* |
| AWL24809.1 | S-RNase | *Malus domestica* |
| AWL24808.1 | S-RNase | *Malus domestica* |
| AWL24807.1 | S-RNase | *Malus domestica* |
| AWL24806.1 | S-RNase | *Malus domestica* |
| AWL24805.1 | S-RNase | *Malus domestica* |
| AWL24804.1 | S-RNase | *Malus domestica* |
| AWL24803.1 | S-RNase | *Malus domestica* |
| AWL24802.1 | S-RNase | *Malus domestica* |
| AWL24801.1 | S-RNase | *Malus domestica* |
| AWL24800.1 | S-RNase | *Malus domestica* |
| AWL24799.1 | S-RNase | *Malus domestica* |
| AWL24798.1 | S-RNase | *Malus domestica* |
| AWL24797.1 | S-RNase | *Malus domestica* |
| AWL24796.1 | S-RNase | *Malus domestica* |
| AWL24795.1 | S-RNase | *Malus domestica* |
| AWL24794.1 | S-RNase | *Malus domestica* |
| AWL24793.1 | S-RNase | *Malus domestica* |
| AWL24792.1 | S-RNase | *Malus domestica* |
| AWL24791.1 | S-RNase | *Malus domestica* |
| AWL24790.1 | S-RNase | *Malus domestica* |
| AWL24789.1 | S-RNase | *Malus domestica* |
| AWL24788.1 | S-RNase | *Malus domestica* |
| AWL24787.1 | S-RNase | *Malus domestica* |
| AWL24786.1 | S-RNase | *Malus domestica* |
| ALQ56987.1 | self-incompatibility ribonuclease | *Malus domestica* |
| ADB85484.1 | self-incompatibility ribonuclease S5 | *Malus spectabilis* |
| ADB85482.1 | self-incompatibility ribonuclease S4 | *Malus spectabilis* |
| ADB85480.1 | self-incompatibility ribonuclease S3 | *Malus spectabilis* |
| ADB85478.1 | self-incompatibility ribonuclease S2 | *Malus spectabilis* |
| ADB85476.1 | self-incompatibility ribonuclease S1 | *Malus spectabilis* |
| AAK58854.1 | self-incompatibility S-RNase | *Malus domestica* |
| AAK58853.1 | self-incompatibility S-RNase | *Malus domestica* |
| AAK58852.1 | self-incompatibility S-RNase | *Malus domestica* |
| BAJ10663.1 | S34-RNase | *Malus domestica* |
| BAC24106.1 | Sg’-RNase | *Malus transitoria* |
| BAJ10662.1 | S33-RNase | *Malus sieversii* |
| BAN66319.1 | S-RNase | *Pyrus communis* |
| BAN66318.1 | S-RNase | *Pyrus communis* |
| ADB91384.1 | S-locus S-RNase S46 | *Pyrus pyrifolia* |
| AHA90950.1 | self-incompatibility ribonuclease | *Pyrus communis* |
| AAQ73176.2 | S21-RNase | *Pyrus* x *bretschneideri* |
| BAC65223.1 | S8-RNase | *Pyrus pyrifolia* |
| BAA93052.1 | S3-RNase | *Pyrus pyrifolia* |
| BAC84996.1 | S9-RNase | *Pyrus pyrifolia* |
| BAB61926.1 | S5-RNase | *Pyrus pyrifolia* |
| ABY73721.1 | S28-RNase | *Pyrus* x *bretschneideri* |
| ABJ97134.1 | S39-RNase | *Pyrus* x *bretschneideri* |
| ASB15792.1 | PpS37-RNase | *Pyrus pyrifolia* |
| ASB15791.1 | S44-RNase | *Pyrus pyrifolia* |
| ABG49099.1 | S13-RNase | *Pyrus pyrifolia* |
| BAA28354.1 | S4-RNase | *Pyrus pyrifolia* |
| AOS95460.1 | S22-RNase | *Pyrus sinkiangensis* |
| AOS95459.1 | S28-RNase | *Pyrus sinkiangensis* |
| BAN33744.1 | S25-RNase | *Pyrus communis* |
| BAJ52233.1 | S ribonuclease | *Pyrus pyrifolia* |
| BAJ52225.1 | S ribonuclease | *Pyrus pyrifolia* |
| BAF63842.1 | Sk-RNase | *Pyrus pyrifolia* |
| BAC00940.1 | S-RNase | *Solanum neorickii* |
| AUE24247.1 | self-incompatibility ribonuclease | *Solanum neorickii* |
| AUE24246.1 | self-incompatibility ribonuclease | *Solanum neorickii* |
| AUE24245.1 | self-incompatibility ribonuclease | *Solanum neorickii* |
| AUE24244.1 | self-incompatibility ribonuclease | *Solanum neorickii* |
| CAA53666.1 | S-RNase S3 | *Solanum peruvianum* |

**Supplementary Table S3. The primer list**

| **Name** | **Sequence** |
| --- | --- |
| FS_a_CDS | ATGGCAATGGTGAAATCCTC |
| RS_a_CDS | CTATAGAATATTGATATCATTATTT |
| FS_b_CDS | ATGGCAATGTCGAGATCATC |
| RS_b_CDS | CTATAGAATATCAATTGGACTC |
| FS_a_S_b_ | TGAAGATCTCTTGGCCYAACCTAGAA |
| RS_a_S_b_ | CCCAGCAAAKWACCACTTCATGCAAC |
| FS_a_Intron1 | TGGCAATGGTGAAATCCTCGTTTCCTTC |
| RS_a_Intron1 | GCATGGATACACTTGGCAGTGAGTCGGT |
| FS_a_Intron2 | ATTCAAAAGCTGCCGTTGAATACTGCAT |
| RS_a_Intron2 | TCATGGTCCCAGCAAAGAACCACTTCAT |
| FS_b_Intron1 | CAGAGTATGGCAATGTCGAGATCATCAA |
| RS_b_Intron1 | CTAGCACTATACCACCTGTTGCCCACTT |
| FS_b_Intron2 | AGCCTCAAACTGTCCTCTAACAGCAAAC |
| RS_b_Intron2 | TTATGGTCCCAGCAAATTACCACTTCAT |
| FS_a_k | ACTGCCAAGTGTATCCAT |
| RS_a_k | TTCGTGCTTCTAGGTTAGG |
| FS_b_k | GCCAAGTAATCGTTCTAAC |
| RS_b_k | ATGTCATCCGTCATCCAA |
| FEF1-α | CATGCGCCAGACTGTTGCTGT |
| REF1-α | GACCGACTCAGAATACTAGTAGC |

**Supplementary Table S4. The *cis*-acting elements in the promoter sequence of *Sa*-RNase**

| *Cis* elements | Number of *cis* elements | Function |
| --- | --- | --- |
| CAAT-box | 39 | common *cis*-acting element in promoter and enhancer regions |
| GC-motif | 1 | enhancer-like element involved in anoxic specific inducibility |
| G-box | 1 | *cis*-acting regulatory element involved in light responsiveness |
| CGTCA-motif | 2 | *cis*-acting regulatory element involved in the MeJA-responsiveness |
| GT1-motif | 2 | light responsive element |
| Unnamed__1 | 2 |  |
| AT~TATA-box | 1 |  |
| TATA | 1 |  |
| MYB recognition site | 1 |  |
| TCT-motif | 1 | part of a light responsive element |
| AACA_motif | 1 | involved in endosperm-specific negative expression |
| AP-1 | 1 |  |
| I-box | 1 | part of a light responsive element |
| Unnamed__4 | 13 |  |
| box S | 1 |  |
| Myc | 1 |  |
| Myb-binding site | 1 |  |
| TGACG-motif | 2 | *cis*-acting regulatory element involved in the MeJA-responsiveness |
| O2-site | 2 | *cis*-acting regulatory element involved in zein metabolism regulation |
| TATA-box | 27 | core promoter element around -30 of transcription start |
| TCCC-motif | 1 | part of a light responsive element |
| ABRE | 1 | *cis*-acting element involved in the abscisic acid responsiveness |
| CARE | 1 |  |
| TCA | 1 |  |
| as-1 | 2 |  |
| MYB | 2 |  |
| STRE | 4 |  |
| W box | 1 |  |
| CCAAT-box | 1 | MYBHv1 binding site |
| AE-box | 1 | part of a module for light response |
| Box 4 | 2 | part of a conserved DNA module involved in light responsiveness |
| AAGAA-motif | 2 |  |
| ERE | 1 |  |
| CAT-box | 1 | cis-acting regulatory element related to meristem expression |
| A-box | 1 | sequence conserved in alpha-amylase promoters |
| LTR | 2 | cis-acting element involved in low-temperature responsiveness |
| MYC | 9 |  |
| RY-element | 1 | cis-acting regulatory element involved in seed-specific regulation |
| chs-CMA2a | 1 | part of a light responsive element |

**Note:** According to online analysis of PLANTCARE^9^, this promoter contains the core *cis*-acting elements of typical eukaryotic promoters, such as TATA-box, CAAT-box that controls the frequency of transcription initiation. Some elements is related to plant hormone response. In addition, there are also some *cis*-acting elements that have not yet been characterized. The 2000 bp before the 5’ end of the transcript sequence was selected to analyze the promoter element.

**Supplementary Table S5. The *cis*-acting elements in the promoter sequence of lncRNA5**

| *Cis* elements | Number of *cis* elements | Function |
| --- | --- | --- |
| Unnamed__1 | 1 |  |
| CAT-box | 1 | *cis*-acting regulatory element related to meristem expression |
| CAAT-box | 42 |  |
| Box 4 | 2 | part of a conserved DNA module involved in light responsiveness |
| Sp1 | 1 | light responsive element |
| MYC | 2 |  |
| ARE | 2 | *cis*-acting regulatory element essential for the anaerobic induction |
| TGACG-motif | 1 | *cis*-acting regulatory element involved in the MeJA-responsiveness |
| G-Box | 1 | *cis*-acting regulatory element involved in light responsiveness |
| WRE3 | 1 |  |
| GATA-motif | 3 | part of a light responsive element |
| Myb | 1 |  |
| Unnamed__2 | 1 |  |
| Myc | 1 |  |
| CGTCA-motif | 1 | *cis*-acting regulatory element involved in the MeJA-responsiveness |
| MYB-like sequence | 2 |  |
| W box | 2 |  |
| AP-1 | 1 |  |
| as-1 | 1 |  |
| TGA-element | 2 | auxin-responsive element |
| ABRE | 2 | *cis*-acting element involved in the abscisic acid responsiveness |
| ERE | 2 |  |
| TATA-box | 41 | core promoter element around -30 of transcription start |
| GCN4_motif | 1 | *cis*-regulatory element involved in endosperm expression |
| AAGAA-motif | 1 |  |
| Myb-binding site | 1 |  |
| MYB | 4 |  |
| STRE | 3 |  |
| Unnamed__4 | 11 |  |
| TCA-element | 1 |  |
| MRE | 3 |  |
| AT~TATA-box | 11 |  |
| MBS | 2 |  |

**Supplementary Table S6. The (in)compatibility analysis according to the seed and fruit setting rate after intraspecific pollination combinations of different S genotype lines of *F. viridis***

| **Female parent** | **Male parent** | | | | | | | | |
| --- | --- | --- | --- | --- | --- | --- | --- | --- | --- |
|  | **S1-05-S2-02**  **(S_a_S_a_)** | **S1-02-S2-11**  **(S_a_S_a_)** | **S1-02-S2-49**  **(S_a_S_a_)** | **S1-02-S2-28**  **(S_b_S_b_)** | **S1-02-S2-57**  **(S_b_S_b_)** | **S1-02-S2-61**  **(S_b_S_b_)** | **S1-02-S2-02**  **(S_a_S_b_)** | **S1-02**  **(S_a_S_b_)** | **S0**  **(S_a_S_b_)** |
| S1-05-S2-02 (S_a_S_a_) | 100% (×) 13 | 20% (×) 7 | 10% (×) 8 | 100% (√) 79 | 100% (√) 70 | 100% (√) 87 | 100% (√) 79 | 100% (√) 87 | 100% (√) 88 |
| S1-02-S2-11 (S_a_S_a_) | 20% (×) 5 | 60% (×) 9 | 10% (×) 6 | 100% (√) 67 | 100% (√) 82 | 100% (√) 68 | 100% (√) 82 | 100% (√) 72 | 100% (√) 90 |
| S1-02-S2-49 (S_a_S_a_) | 10% (×) 6 | 20% (×) 10 | 10% (×) 10 | 100% (√) 69 | 100% (√) 69 | 100% (√) 78 | 100% (√) 76 | 100% (√) 82 | 100% (√) 77 |
| S1-02-S2-28 (S_b_S_b_) | 100% (√) 82 | 100% (√) 71 | 100% (√) 66 | 10% (×) 4 | 20% (×) 14 | 20% (×) 4 | 100% (√) 66 | 100% (√) 76 | 100% (√) 64 |
| S1-02-S2-57 (S_b_S_b_) | 100% (√) 80 | 100% (√) 73 | 100% (√) 85 | 30% (×) 7 | 20% (×) 22 | 10% (×) 3 | 100% (√) 69 | 100% (√) 74 | 100% (√) 77 |
| S1-02-S2-61 (S_b_S_b_) | 100% (√) 84 | 100% (√) 74 | 100% (√) 69 | 10% (×) 8 | 20% (×) 6 | 20% (×) 4 | 100% (√) 70 | 100% (√) 81 | 100% (√) 80 |
| S1-02-S2-02 (S_a_S_b_) | 60% (×) 8 | 20% (×) 9 | 10% (×) 11 | 10% (×) 4 | 20% (×) 8 | 20% (×) 7 | 70% (×) 9 | 20% (×) 7 | 40% (×) 8 |
| S1-02 (S_a_S_b_) | 20% (×) 4 | 10% (×) 7 | 10% (×) 8 | 10% (×) 3 | 20% (×) 6 | 20% (×) 12 | 0% (×) 0 | 20% (×) 3 | 30% (×) 6 |
| S0 (S_a_S_b_) | 20% (×) 8 | 10% (×) 12 | 20% (×) 6 | 20% (×) 12 | 20% (×) 18 | 10% (×) 10 | 10% (×) 12 | 30% (×) 5 | 20% (×) 5 |

**Note:** The percentages in front of the brackets indicate the fruit-set rate; The √ and × in the brackets indicate compatibility and incompatibility, respectively; the numbers after the brackets indicate the single-fruit seed-set rate.

**Supplementary Table S7. The (in)compatibility analysis according to the seed and fruit setting rate after interspecific cross pollination.**

| **Female parent** | **Male parent** | | | | | |
| --- | --- | --- | --- | --- | --- | --- |
|  | **S1-02-S2-49 (S_a_S_a_)** | **S1-02-S2-28 (S_b_S_b_)** | **S0(S_a_S_b_)** | ***F. vesca* 41** | ***F. nilgerrensis* 45** | ***F. mandshurica* 43** |
| **S1-02-S2-49 (S_a_S_a_)** | ------- | ------- | ------- | 0% (×) 0 | 0% (×) 0 | 100% (√) 79 |
| **S1-02-S2-28 (S_b_S_b_)** | ------- | ------- | ------- | 0% (×) 0 | 0% (×) 0 | 100% (√) 60 |
| **S0 (S_a_S_b_)** | ------- | ------- | ------- | 0% (×) 0 | 0% (×) 0 | 100% (√) 62 |
| ***F. vesca* 41** | 100% (√) 101 | 100% (√) 115 | 100% (√) 109 | 100% (√) 118 | 100% (√) 104 | 100% (√) 117 |
| ***F. nilgerrensis* 45** | 60% (√) 64 | 70% (√) 58 | 70% (√) 54 | 80% (√) 62 | 100% (√) 180 | 70% (√) 76 |
| ***F. mandschurica* 43** | 10% (×) 6 | 0% (×) 0 | 10% (√) 5 | 100% (√) 79 | 10% (√) 6 | 100% (√) 82 |

**Note:** ‘-------’ means the same data as Table S6, and will not be repeated here. The percentages in front of the brackets indicate the fruit-set rate; The √ and × in the brackets indicate compatibility and incompatibility, respectively; the numbers after the brackets indicate the single-fruit seed-set rate.

**References：**

1. Kao, T. & Tsukamoto, T. The molecular and genetic basis of S-RNase-based self-incompatibility. The Plant Cell. **16**, S72-S83 (2004).

2. Claessen, H., Keulemans, W., Van de Poel, B. & De Storme, N. Finding a compatible partner: Self-incompatibility in European pear (*Pyrus communis*); molecular control, genetic determination, and impact on fertilization and fruit set. Frontiers in Plant Science. **10**, 407 (2019).

3. Ushijima, K. et al. Characterization of the S-locus region of almond (*Prunus dulcis*): analysis of a somaclonal mutant and a cosmid contig for an S haplotype. Genetics 158, 379-386 (2001).

4. Ushijima, K. et al. Structural and transcriptional analysis of the self-incompatibility locus of almond: identification of a pollen-expressed F-box gene with haplotype-specific polymorphism. The Plant Cell. **15**, 771-781 (2003).

5. Yamane, H., Ikeda, K., Ushijima, K., Sassa, H. & Tao, R. A pollen-expressed gene for a novel protein with an F-box motif that is very tightly linked to a gene for S-RNase in two species of cherry, *Prunus cerasus* and *P. avium*. Plant and Cell Physiology. **44**, 764-769 (2003).

6. Ikeda, K. et al. Linkage and physical distances between the S-haplotype S-RNase and SFB genes in sweet cherry. Sexual plant reproduction. **17**, 289-296 (2005).

7. Wang, Z., Zhao, M., Qian, Y., Wu, W. & Yuan, J. Study on pollen viability of strawberry. Journal of Northeast Agricultural University. **41**, 48-52 (2010).

8. Hibrand Saint-Oyant, L. et al. A high-quality genome sequence of *Rosa chinensis* to elucidate ornamental traits. Nature Plants. **4**, 473-484 (2018).

9. Lescot, M. et al. PlantCARE, a database of plant *cis*-acting regulatory elements and a portal to tools for *in silico* analysis of promoter sequences. Nucleic Acids Research. **30**, 325-327 (2002).

**Supplementary Sequence S1. The DNA reference sequences of *S_a_*-RNase.**

AACCAACTTTTCAATATATGCTTTTAGCGTAATTATAGCAAGTTATGCAACATTTCACATTATGATCATTTGTGGACTCAATATGATATCTATTTGGATTCAGAAGAGTCTCTTCAATAAAGACAACATAGTTTCATTCGTCATTCTAATGTGGGCTATTCGAGCTTTAAACTAGGTCTGTAATTCCAAATATGATGGTCACATTTGATTTAGCTTTAGTAAATAGCAATATTGATATTTCTGATCACATGGGATCGTATGCATGTAGTAGAATGTTCAGCAAGACATATATGGAGTTGAGGGCATCCATGAAGTTTAGCCATTGCGTTAGTGAACAAGATTATGTATGTAAGAAATATCAAATCATGGTAGATAAAAAATATTTCTTAAATGAAAATAAAAATGAATCAGGCTTGTCTTTTTTTTTATTTTAAAGAATGGATAACTCAATTGATCGAAAAGATCATAGTCATCATACAATGAGCTCTCTCGCTCAAATTAAGAGATCCGAAGATCGCCTATTACTATCGATACTTAAGTAGTGCACTGACTGTACATTTGACAATGACTCTAGACTACTACTCCTTAAACTAGACAATACAAAATTTAACCGCCTTTAAACCCTTTTATTGGGTTAAACTTGTACTGGCTAAACACTAAACCCTCCACGAACCTGCAGGCATTATTTACAACAACAACAACAGCAACAACAAAAAAACACACACACACACAGAAATAAAATGGGCGCAAAACAAAGCCTACACATAAAAGAAACCCCAGGAGACGCAAGCCACCAACCCAATATGAGAACGAAACACCATCGACGCCGCCGCCAACGAACCAAGGCCAGCACCCCACCCCCACCCCCCTTCCCCCTTCCCGCGCGCTATCCTTGCCGCATAATGGAATCACAACTCCCCTGCTTGGATTAATCAGAGAAAGGTCCAAGCCCTGCCAACGAGATTAAGCACAGTCATCCCCCATGCTTCCGCCATTCATCCCATCTCTGCGAATCAGCCCCTCCCTGAGTTAGAGCTCAGTTTCGGTGAAGATCTGAGCCACTTCATCACAACCACAAACCCTCCCTGCCCCATTGGCAAACACCATCGAGCTCAGGATCCCATCAGATCGAGTTAGCCGCTTCAATCTCTCATCACCTCTCCACCGATCTCTCTCCGATCAAAAACCCGTCGAAGAGCCGGCTCCATCTAGACCCGCAAAGTCCTGTCCGTCGACGGCAAATGGCACGCCGCCGGACAACGGGGGGAAGAAAAAATGTTTTCCCTCAGGCGCGTGGATGAGCGCGTTCTCTTTCTCCGGACAGAATCAGGCATGCATGTCTATTGATATACTATAATAAGATGCATCGATAATGTGAGATCATTGCCGATACCAATTCATGAGTATCATATGCTAAAATAACGAGGGCATTTAAATTTAGTAATTGAAACATGTGTTAGTATGTATAAAATTATAACAAAATAATATAAAGATGATGATCCCCAGAACATCCGAGTTTAAACCCCGTAACAAAATATATTTTATTATTCATTTTTCACAAATTAGTAACTTTCGGTCATCAATAGACATAATGTACCACATAATGATGAAAGAAATGGTAACGTTCACATTATCAGGATATTGACGGATTAATATAATTGATTTCACCTTTCTTGCCTTTGAGTGAGGCTCGGTAAATTGCCAATAAATGTTAAGATATGCGTCAAATATGTCATATTAGGATTCATATTCAAACATGTATGTCGGTGAGAACTAATAGATTATCATATTCGAAACAAAGGTTTCGAAGTATTCATAAATACGAATAATGAATCCAATAGAGAAGAACACGAGAAAAACATGTGTTTGATGCCTATAGTCATAGGTATAAGACTATTCGGTCAAAGAACTCCAGTTTTGATTATTGAAATAATGGTTCTTAATGTCGCATATTGAATAAAAAAAATTTAACAATCATATAACAAATATATGAATGATCATGTGAAAGAAATATGTAGTTTGTTATTAAGTGTTTGAGGACGTGTGAATAATTTTTTTTTTTTTCTCAAGTGAGACCTTTTTGTTTCTTTTCTTCTCTAAACTCGCTCTTCTCACATATGATTCGCACTCTCCCTCTCAAACGCCTATATAAACAGCACATGATCTGTGCAAGTGCGATCACAGGGCTTTCCCCTTTATATTTTCGTTGCTTTTTTATACAGAGTATGGCAATGGTGAAATCCTCGTTTCCTTCAATTGTTCTCGCTTTTGCTCTCTGTTTCACTATGAGTTTTGGTAGGTTTCAGACTTTCTGTGCTCTATGGTATATGTATACTTACTTTCAAATTAGTTTTCTCTACACTCAGTAAGTTAGAATTTTGAAACTTTTATGCGAATGCTTTCTGTAGGTTTTCATTTTCTTTTAATATCAAAAGAGTAATTATCTAATAATAGATTAGAAGGCTATGAAATTATCCACTTCATGATCCAATCCAGCGGGGTTCAATACAATAGAGATCTTCATTCTCTTGCAGAATGATTCTATTAATTTCATTAGCTATCTTGTTTCAATCCTTTTTTTTTAAGGACTGGTTTGGCGAAACGACTTGATTTATGATACCGGATCCCAGGCAGAACGGGAATATAGTCGATATACACCTCAGGATGACCGAAAACAGTAAGAGATACTGGTTTAATATGGAATCCCCCACCCCAACGGAATCAGAAAACATTTACTGTCGCGTTCCATTCGCCTTTGACGCGTCTCGATTTAGAGAGTAATACACATCCTCTAGGTTATCCATAATGAATGAAAAGAGGAACCCAAAATATTTATTTTTGTGATGGAGTTTCATTCCTCAAGTTGAACGCAAACCTTTAAGAGCACCACAATTTCTAAGAAGCCCTTTAATTAATATTTTACAGAAAAGAAAATAGTCCAAACAGTGTCTCACCTTTCACATAAGCTAAGTTTTAGTATCTCAATTTTATAAAACTTTAAAACAGTGTCTCACGTTTATACCCCAACATAAGTTTGGTATCTAGAACCGTTACCTCTATTAAGAATAACTGACGTCAGTCATATTTGATCATAAAATGACCAACTTACCCTTAACCCTTTTGATCACTTTTTCTAAAGAAAAAGCTATTAATTTAAAACTAATCTTTACAGACTGGTCTTTAGTTTGTGATAGACAACTCCTCTTCTCTCCTCTCTTCTCTACTTAGAGTCTTAGACACACACTGAGAGCCGGAGATCTACGATCTCTCCTCCTTCCATTTCCTCAGGTGAAACAAACATGGGGATGTCTTTCACCAAGCTTTCTGCCATCTCTTTACCTACACAGAGATGCGTATACAAACATGGTTGGTCGACTCGATGTCGTTGGTAAAACCACCATTCTCTACAAGCTCAAGCTCGGCGAGTTTGTATATCCATAGCAATAATAGATCACCCAAAGAAGCACTATCCATTCCGTCGTGCTATGCGCCCTCTCCTTTAAAACTCACTATCCATTCCTTCTTTTACCCCGACTAATTGAGTAGCATCCTATGGCACAATTTCCTTTTTAAAACCCAGACATGTGATTCATGGTCATTTCTTACAATGACAAAATTGAAACAAGGGTATCAACATTCAAGGTCACAGTAAAACTCCTAGTCTCCTACCTTGAAAAACATAGTTCATATAAAAATATTTACAACCTAGAACCCTGAGTTGGTAAATTTGGCATTCTTTAGTCAAAGCCCCAATTCCAGAATGCAAACAGAGGAAGAACACGAAATAATCAAAAGGTATATTCAAGTCAAAAATTAAAGTATAATAAACACAAGTACCTCAATATCTCTATTAGTAACACCATGATCCAAGTTAGTTACATAAAGATTGGTGCTTCCCTCAACCCCTGGGCTCCAAACCACAGTAGCTCAATACATCCAATATTACGTCTTCGACGTTGACAACGCCATGACCAAGTTGTAGCCCTTGAACCTGATCAACCGACCCTCACCCCCCCCCCCCCCGAGGCTGGGAAAGGAGTGCGGCACTCGAGACGGGAGGGTTGTGGCGGTTGTCGAAGCTAGGTCTGGGAATTGAATCTGGCGTTGGTGAATTCGGTGGATAGAATAGGAGAGAGATAAGGTAGGAAAAGAGAAAAGAAAAAAATGTTTAAATATTCGTCCAAAAAAATGTTAAAATACAAAAAGACCAAACTACCCTTTAGCTGGTAGTCATTTTAACGGAGGTAACGGTTCTAGACACCAAACTTGGGTCGAGGTTACAACATGAGATACTGTTATGAAGTTTTCTGAAGTTGGGATACCAAAGTTTAGCTTGGGATACTGGTTGGATCATTTTTCCCTTACATAATAGCTCCTTCTACCCATTGAGGCGAACTAACCTCACAATCTCCTTGCTTCGATTTCCAAATGAATAGAGGAACGAGTCTGTGGCTTTTAATGAAAGCATCTCTAACAACTTCTCTATAATTTCTCTAATATAAAAAAGCAGAAATCAAAGTTTAAGCAATTTCTCTTCTACAATTCCAACAAATTCCTTATTTTTGTTGTAGTATAAATTGTAAAATAGAGAAGATAGCAAAAGATGAAAGAAGTGTTGTCTCTTATTCATGGATATGAGTCATTTATATAAGGAATTACAAAAACACTTTTTAGTTGTGTAGAAGAAAACATAATCATATACTAACTTAGTAACCTATCCCTACATGTAGTCAGGATTTCTAATTCTGTCAGCATTAGATCAAATCGCACACAAAGTGATTATCCTAGATTAATTTTAGAAATTTCGTGATCATTCCCCAAACTTTTCTCTAAACTTATAGAGTTTGTTGCAAATATAAGGAATTTAATTTTCTCTCTCCTCAACTCCTCAAGATCTCTATTATAGGGAATCTATTGAAGTAAAATAACGAAATTTTTTCTCTAAAATAGAGAAATTCAAGATTTGAGGAAGTTGTTGTAGTTGTTCTGAGTTCAGTTGCCAATTCAAAGAGTAATTGAGTATTGAGTAATGCAATAACAGTTGTTGCAAAAGCGTTCATAAGTTGGTAATGTTTATTTCTATCCACAATTAAAACACCATTCTCAAGAGATTCTCTACTTGGAATATGTCCACATTCTGATTAGCACGATAACAGTAGACAAGCTATTTTAAATGTCACATATTACTTAGGAGATTGTTTCTTTTGTTCCAGAGGTGCCAACAAGGACATAAATTTGTTGGCCATCTTTATTTTCTCACCTTAGTGCCCACGACCCTATTTAAGTGTCATGAGTCTTTTTACTTAACTATGTCTAACCATAGTTAATAATATAAAATTAGGGTGGTGCTATTAACACACTCTAGTTTGCTATTCAGACACCCCTTCATGAAAAAAGTCGATCATAAAACAACTTTAAGTGACAATTTTGTAAACTCAATATAAGTTGTAGTGATAGAAAAATTAAAAATATATGGGGTGTGTGAATAGCAAAATAGGATATGTTAATAGCATCGTCCTAAAATTATAATGATAAATTCTATTTTCTTTAGATATTAACTTTAACTCAAATTTATTTTGTTATTATTTTTGTTAGGGTTTGAGGTTTAAGGTTTGGGGTTTAGGGTTTAGAGTTTAAAAGAAAAAATGTTTATTAAAAGATATAAAATTGAGTGTAGGGTTTAGGATTCAGAGTTTAACGTTTGCATTTAAGTTGTAGGGTTCAGAGTTTAGCGTTTGCATTTTGGTTATAGGGATTTAAAATAAATAAAATTTATTTTTTAATATAATAAATATAATTTATTTAAATACATAAAATTGTGACAAAAAAGAAGAAGAATAGTTCATTAACCATAGTTAGGCATAATTAAGCAAAAGGACACATGTTATTTAAATTATATGATAGAATTTGAGATGGGCAAATAAAGGTGATCACAAATTTAAGCAGACAACTCCATTTCATATGTTGACATGATGGCATGATGCTAAGTTGGTAACTGCTTCCTCCCACGGTCCCACCAACTAAAGGGAAATCATGATGATGTTTTCCAAAACTTTTCCGTGGAGAAAGCGTCCATAGTCATTTCTGTTCTGTTCCCGTGCTAGTATATACTCGACTACTTTATGCCCCTATACTCTTGAGTCACTTAATTGTACATATTAGTATTTTTTTTTTTCCTGAACGAATTCAGTGCATCATGATGCACTTGAACCAAATAGATTAAACAAACAGTGTCAAAAAAATCAAACCGCAGTAAAATCTCATAAATCCAAAACACTGAAAAAGGAAAGGCAATAGGACAATGTGAGAGACCAAGAGAGAACCCCATAACTTCATCATCTCTCCCAAACACTCCCCCATTTCTCTCTGTTCTCCCCTTCCCTCTCCATGACATCTCAGTCCCTTTCAATTCACACCCCTCCCCCCTCCTCCAATTCCTTCAAACCCAAATTCAATCTTCCATTGAATCCAACTCCCGACCGCGTCTTCGTTGCCGGGAACGGGTAATTAGAATGCTGACTGTTGCTAGTTGATTATGATTTCGGGAAATTTGAAATTTCTGAGCTCCAGGGGTTTTGGTTGTAGCTAGAGCCCAGACTCTTGATTGGGTAGAATGGTTTAGAAGGTTTCGAATTCCTTATTGGGTGAAAGTGCTGCAATGCAGTTTTGTTTCCTCATCAAAGAAAACTAATTGGGGGCCAGCCTTTCCAGCCATCCCTCCCGATTTCGTATTGGGTGAAAGTGTTGTCGTTGTGCCAACAAATTTTGTAGATGAATCCGTTTTCAGTTTCTTTAAATCATAGGATGTAGAATATCATATGTGCACCTTCTTCTTCTTCTTCTTAATCCTTCAGTCTTCTGGTACCTTATGTGTTTCTACAAGTTTTACCATGGTTATATTTTTGGGGTTTGATTATATTTGACGGTGTGTGTTGGAGTTTGTGTCATGTAGAATTTGGATTAAATCAATGGCTGAGATTACACTATCAATGTTGGTTCACTTATATCTCGATGTAGAGTGCGGCTATTGGGACCTCTTAATTTGCTCACCTGACCTCACTGTATTGTGATTTATTAAATGACATATATATCTTTGCACATAATGACTATTAAGGACAATAATTAAACAAAAAAAAAAATATTAGATTTGTTCACTTTAAACTTTCCAATTCAATAATAATGGATTGCATTTTATTACTTTTCTTATTGATTTTTTAATTTTACTACATACTTATTAAAACATTTATATATATAATAAGAACTAAAACTATGAGTAATATATTACGATCATGTAGCAGAAAAATTTATGTTATTATACATGTTGCATGCATATACGTACAAGGTAAAAAAATATTTTTGCGGAAAGTATGATTTATCAATGATTAAAAAAATTATATAAGTATTAAATACAAATTTAAATAAGAACATTCTTTTGATGATTGATAGAAATTAATTGTGAGACACAATACCTTGTTTTTTTTTCTTTTAAGATAAAACTACTCTTCATGATTCGAATGAATTTTTCTTTGAAATTGATGTCATTGATTTTTTTTTTCTTAGTATTTGATTAAAAAAATGAAGGTCTAGTGACATTTTATGCAAAAAAAAAAATGGAGGTTTGCGTGAAAATATATTAAAAAAGAAAAAAAATAGAGGTCGAGAATAGATCGATGTTTGCGTGCAAAAAGAAAAAAGAAAAAAAAAAGTCTAGTTAGTGAATAAGAGGTGTAATGAGTAATATATGGAAATATATGTGAATTACATAAATAAGGTTATTTTAGTAATTTAAAAAGAGGTCTAGTGAGCAAATGCTTGTATTTAAAAGTAAAATAGAGATGTAAAGAGAACAAGAGGTATATTGAGCAAATTAGGAGGTCCCAATAGCCGCACTCCTCGATGTATACAAAACTTCTTTTTTGTTGTCGAAAACTATCAACTTGATCGCCAGTCTTTTTGAAAGGTGCGTTCTGGAATGTACCCTGTTATTCTGTTAACAGTGTACCAACCTTGTCGGGTTGTTGCATTGTCTATTTTGCCCTTCACTTCCTCACTCGACGGCGTCATGCTTGCTAATGCTTCATTTTCTGTTGCTCTGGTTTCTTCTCTGTTCTCGGTTGCTGGTATTTGTTTTGTTCTCGGTTTTGTTTTGTTTTGTCTGATTCTGATGCTTATGCTAAACATAAAGCAACTTCATATGGCAGTCTTAACGTTATAAATTTATGACATCGGAGGAACGCTTCTGATGGCTATGGCTCCCTCAGTTTGTCTTAGTGGTCTCAGAGATGGTGTTCGTGGTTGGCGATAAGGTCAAGATCGGAATCCCCAATACTTTTTTTTTTCGATTAAAGTACAGATACCCAATACTTTTTTCTTCGATTAAAGTACACAAACTCAGTTTCCGAAGGTTGAGTTCATTCGGGTGTTCAATCAAAATTTTAAAATCAGTTAATCAAAACAAAAGGATATTAAGAAAATAGCATGGCAGAGCAATATGGTAACCAAAATCAGGACCTGGATACTCTCCTAACCTTGTTTAAGCTTCTCACCAGATGATGACACGTGTTTTTCACATATTTAAATGGCTTACCTTTCTTCCTCACATCCACTTTGTTCTTTCGTTTCTCTCTGCTCTATTTTTCCTTCGCCGTATCTCTTCTTCGTGCTCTCTCATCTGTTCTTTTCTTTATGTTCTCTCCTTATCACCACTATTTCTTCTCTCAAAATTAAAATTTTGCAATCACATTCATTAGGAAAAAGAATCCCATAAAGCTAGCTTCAACCTTTCAACTTTTTGGTGATGGATAGTGAAGGTTGTTGATAGTAGTTATGGCTTACAGTTAAAGTCTCCTGATAAACATTTGGTACTAGAGTTTCAGCCCCATTTTCCACCAAACAAATAAAATGGGTTCTTGAACCTTGAAACTAATATGCAACTCTAAACTTGGAGATAAGATCTTGAAAAATCACTTCTTTAAAAATAGGTTTTGCTTGGCAGAGAAAACAACCAAAACCCAGAAGCATAGGAATGAGAAACAACAAGATTTGAAGAAGAAAAAGAAGAAAGGGTCCTTAAGAGTAAGCCACTTATCACTATCAAGAGAGGGTTTGTGTAAAGTCTTAAGAGGAACTGTGTCTTGGTCCCAAATCTCAACATGCTTTTCAATAACAAAAATAGTAAAAATGAGTGAAAATAAGAAGAAATTCAATTTGTGTCATTAGATGTATCTTAATCCATGTGTCGTCATTTGGTGAGAAGCTTAAACAAGGTTAGGCAAAATATAAGTTAGGAGAGGATCCGGGTCCCCAAAATCATAGAGCATGAACACTTACCAACACTTGTACCAGGTCAACACTTGATCATATATATCCCTCACTATAATATTATTGATTTGAAGAACAGATCTAGTATTGAGCACTATAAGAACGAGTCTCTTGCACAGATTGTTGATTGTTGTACGTATTAGAGAGAAATATATCTCACATTAGAAAAGTGGTAAATAAAATATAATTTATAAGTGGATGAATCATACTTAATTGTATCAAAATCTTTTGTGATTAACATTTAACACCTTAAATGTGGTTAAGTTGAGACAGTATGGATACAATAATGATTTATGGGTCACGCTTATCTTTGTTTAACATCGATGATTTGATTTTTTTTTCTCTTTTTTGGAATCAACAACTATTGAGCGCAAGTACAAAATTGCTTCAATTAGATATGGGATATTGGATTATAGTTTATGTTGTGCAGTTTGTTTTGTAAATTGTTTTGGTGAAACGCGCCTGCGAAAGATAATACCCAGGGAAGCACATCACCTGTACCCATTGTGCCAACCCTGTTCCGCAAGTGAAAATTTGACACGTAATCAGTAACTTAACACCCTACTAACTCTGTTAACCATTGCAAAGTAATAAAATCTTCTCTTTTTTTTTTATTAATTTTTCTCCAATTGATTCGACCCAAAAGAGTTCCAGAAGAAACTACATCTTCTTCTTTGTGGTCATCTACCCATCAAATCCCCTGGTTTTCTTCTTATTAAATCTTCTTCTTCTTCTTAATTTCTTCTTAGATCTTCTTCTTCTCCATCAAATTCGATGATCTTCTTCTTCTTCTCTCATCTTAAACCTTCAAAACTCCCCAGTTTCAAATCCAGTATCATCTTCCCTATATGTTCGTCCATCAATCACATCACAATCCCAAGTCTAAAATCAGACATAAATATCACAATTTCTTATCAAAATGTTGATGGAGACTGGCCTTGAAGGTTAGGACGTGGCCGAATTGAAGCAGGGTTGGATGACCAATACCTTGTTTAGTAGAAATTTGGGAGCTCAACGAACTTGAGAATGGGCATGAATTTGCTTCCTTTTCGCAGTCAGAAAAGCTTTTTTTTGGAGATCTACTGGTGGGTTGGGACTTTTCCGGAAACGACTGAACGAACACGATGGCGAATATTTGGCTGAGATCAGATTCTGGTATAGGGAACTAGGGAAGCGTAATATTTATTTAAAAGTAAAATGAGAAAATAAAATTAATATGATTAACTTAACAGCGTTAATTCCTGTTAATTCAATGGACATGTGTCAAATTTTCACTCGTGGCACAGGGTTGGCACATGAAAAATGGGTACAGGAGATGTGCTTCCCCCAGGGCAATCATTCTGATTACACGGATGCAGTCATCTTCAACCTTTCCACCAAGAAATTCGCTTTTGAGCCGTACAAATTCCATCCCATGGGTCCGACGAACCACGGATATGTTCTTGATTCTGGAATTTTAGGCACTCTAGGCAACTCTCTGTGTTAGTGGTTTAAAAATTGCTGTCAAATCGTTCAAGGGGAATGAAGGTGAAGCTTGGTCTGATGGTGAGCTAGAACTAACGCCCACAAAACGGAAAGCAGTATGCGGCTCAAGCAACGATAAGGGCAAAGTACGCAGATGAAATATCAGTTTTTAAATGTGTGGTCTGTATTGGCCACTCACACCTCGACATGCAAAAAGTTGGGTACGTGAGATGCCGGTACTTTATAGACGAAATTGGATTCTTTAACCACCTAAGCTTTTTATTACATTATGGTACGTGTACAAACTATTTGTTTTACTTTTCTTCTCTAGTCTATCTCGCTTTCTTTCTTGGGATTCTTTCTTCCTCTCTTCGTTCTGTCGTCGACTCGCACAAACTAAAATTGGGTGTTGCTAAATATACTTACCCAAATACACCCCATACTTACATATTTTCCAACTTTACCCTTTATTCTATACATAAACCCGGCATTTGACCTACACAAACCCAGCAATCATTTTGAAATCATAGTAATCGAAAAAAAAAAAAAAAAAAACCGTCGTTGGAGTAAAGACTGGGTGAGAAGAAACTGAAATACTAAATCCTAAAAAAAATGAACCATTTTAACACCGTTGCTGTTGGAGTAAAGACTGGGTAAGAAGAAACTGAAATACTGAATCCTTTATCCAAATGTGGTTTCACTGGTGCCAACAAGATAATAATTTCTTGTTAATTCGTGTGTTTTGATTTCATCAATTGATCCGCTTTCAAATTGATCAGATCTACTCTTGTTAAAAGAACAATTGGATATATCTCAGTGTGATCACTTTATCATGCATAAAGCATTGGGCTATCCCTGTTAACGTTTTCTTCAGAACACCAGAAGAGAATAATCGCCCATAAACATGTAAAATGATATCTTATAACAAGAGTAATGTTATTTCTCCTTGATCTCAGTTCGTAATTGTGATAATGATATATAGGTTTAATCGAACACAGTTGGGTCTCGTTTGGTTCATGGATTGAAAAAGTTGAGAGGGGAAAGTTGTTCCTTTCCTTTATTTAACACACCTAAGAAAATGAAAGACTTTATTACAATATGAGAAAAATAGATGGGAAATTGGATCCTCCCACATACCATGAGATTCAATTTCGTTTATACTTTTCCAACATTAATTGCATCTTTTATATATTGTTTTAATGGATGTTATTACCAAATTATCCACAAAACTTTAGAATCTTAAATATTATATATGTTATTACTAAGGATATCATTGGTAAATTAGTGTTACTTGCTTTCTTATCCATGCATAAACCAAACATTAAAAAGAAAAGTACAATACATTTCCTATTTACTTTCCTGATGTTCCCAAACACCTCATAGGGAAACTAGTGGGAAATTTACTTTCCCATGTACATGAGAAAGATCAAGGAACCAATTTCCTTTCCATCAACCAAACGAGGTCTTGTAGTTTGCTGGATTCATGGAAATAGAGGGTAAAATTGGAAAATACATACAAAAAATGGTGTTGATGGGACAGCTGGGTGTATTTAGAAATTTAATGGCTACATTTAACAACACCCAGTAAAATTTCATTCAAACCACAATTCAAAGTTTCAAGCTGTCTCTCTCTTACCTATTTACTAGCAACAATACATACAGCACTTGACCAATTTTTAATCTGATTTTATGAAAATGGAAGAAAGATTCATGAAAGAAACCCATCAAATAATAGAACGAGCAGAGAGAATAAAGGGGAGGGAGGGAGAGATAGAAATTAATCAAATGATGTTTGGGTCTATGGTAAAATCAACAACTTTTTTTAGTTGGAGGGAGACATAGACCATATTAAAATTACCAAGTGTCTTAATCTCTAAGAATTTAAGGGTTAGTTTGGTATTGCTGTGACTTAAAAAAAACTTGTTACTGTTGTACTGTGAAAATAATTAGCTGTGAATTAAAACAGTTGTGTTTGGTAAATATTAGTTTTAAAAGTGTTGTTAAGATAAAATTTATTAATTTATAGTGTTTTCAGTGACAGCTGCTTTTAAAAATAACCTTCAGATTGCTTCTAAAAGCCACTGCTAGTGATATGTAATTTTCAAACAAAACTATTTTTATTTCTTTTACCAAACACTATAAAATTTAAAAGTTTGAATAAAAGCTGATTTTTTTTAAAAAACGAATCTGTATCAAACTAGGTCTAAAAAGAAATACAAGTAAAAAAAAAAAAAACCTAAATCCTATACGACCCTAGGGAAAAACAATTATTCTAATGAGTTTAGATGCTTAAGACTCATTAGTGTATGTTTCCACCCAACAAATAAGTGAACAACTTCAAACTAACAACCAAGGAAGAAAATTTTGGTGATTACGGAAATTTCGACCCTCCAAAATATGGAAATTTCGACTGAAATTTCGGGGAAATATCGATTGCAGTAAAAAAATTGAGAAAATTGACGGAAATTTGAAGAAAAACAAGGAAATTTTAAACCAAACTTTAAGAGATGTTTGTTTTCTATATTATGTACTATATTTGAAATGAAAACCTTGAATAAACATCGATCTATAGGGAGTTGTAGTAAGTTAAGGTGAAAAAGTTAGTGCTGAACCGTTAATAATTCTTAGAAGTTTACTTTAAACATTTTTTTTTATGGCTGGAAACCCAAAGGAGGCTAGGCCATCACGCTTTATATACATATGATATATTTCACATGGAATTACATGAGTACTTTAAAACCACGTAGAAGACCTATGATGTACAAAATTTTCATTATCTTTATCGTCTCTCTGTGTAGAGCCTTCAGTATATTGTGAAGAATAATCATTAAATAATACCTCACCTTTATAGATATATTAAAAATGAATTAAGAAGTGTAGGTAAAAAAATCTTTTTTATGGAAGTGTAGGTAAAAAAATTAGTTGGGGATTAGACATTTACCTAATTTTTTATTTTTCAGATTAAAAAAAACCAATATATATAATAAAGATACTATAATAAACATGTTAAATGGTTAGTAGGGTTGGTTAAGTTGTTAACCTTGTTCCAACCCGACCCAGGCTCGACTCTCACCTTCGCTGCAAAATTTAACATTTTGCCGTGGTCAAAGTCAAACATTTCAGCATGAAAGTAAGTTTTGCAGTGTTCAAAGTCAAACATTTCGGCAGGAAAGTAAGTTTTGCAGTGTTCAAAGTCAGGGGAAACAATCATTCGAATTTGGGCGGGAAAGTGGGAAACCATTTCAGCTTCGACTTTTCGGTGGGATTTCAAAGTTTCGGCGATATTTTTGAAATATCGCGATATATCGGAAAATATCGATTATTTCAGACGAAAAATTCTGGGAATGAAAATATATATCGCCGTGTGATAAAAACGAAATTTTCGGCGATATTTCGGCGAAAATTTCGATTTTTTCTTCCTTGGTTAAATGGTTAGTAGGGTTGGTTAAGTTGTTAACCTTGTTCCAACCCGACCCAGGCTCGACTCACCTTCGCTGCAAAATTTAACATTTTGCCATGGTCAAAGTCAAACATTTCAGCAGGAAAGTAAGTTTTGCAGTGTTCAAAGTCAGGGGAAACAATCATTCAAATTTGGGCGGGAAAGTGGGAAACCATTTCAGCTTCGACTTTTCGGTGGGATTTCAAAGTTTCGGCGATATTTTTGAAAGATCGCGATATATCGGAAAATATCGATTATTTCAGACAAAAAATTCTGGGAATGAAAATATATATCGCCGTGTGCTAAAAACGAAATTTTCGGCGATATTTCGGCGAAAATGTCGATTTTTTCTTCCTTGGTTAAATGGTTAGTAGGGGTGGTTAAGTTGTTAACCTTGTTCCAACCCGACCCAGTCTCGACTCTCACCTTCGCTGCAAAATTTAACATTTTTCCGTGGTCAAAGTCAAACATTTCAGCATGAAAGTAATTTTTGCAGTGTTCAAAGTCAAACATTTCGGCAGGAAGGTAAGTTTTGCAGTGTTCAAAGTCAGGGGAAACAATAATTCAAATTTGGGCGGGAAAGTGGGAAACCATTTCATCTTCGACTTTTCGGTGGGATTTCAAAGTTTCGGCGAGATTTTTGAAATATCGCGATATATCGGAAAATATCGAGTATTTCAGACGAAAAATTCTGGGAATGAAAATATATCTCGCCGTGTGATAAAAACGAAATTTTCGGCGATATTGCGGCGAAAATTTCGATTTTTTCTTCCTTGGTTAAATTGTTAGTAGGGTTGGTTAAGTTGTTAACCTTGTTCCACCCCGACCCAGGCTCGACTCTCACCTTCGCTGCAAAAGTTAACATTTTGCCGTGGTCAAAGTCAAACATTTCAGAATGAAAGTAAGTTTTGCAGTGTTCAAAGTCAAACATGTCGGCAGGAAAGTAAGTTTTGCAGTGTTCAAAGTCAAGGGAAACAATCATTCGAATTTGGGCGGGAAAGTGGGGAACCATTTCAGCTTCGACTTTTCGGTGGGATTTCAACGTTTCGGCGATATTTTTGAAATATCGATATATATATCGGAAAATATCGATTATTTCGGACGAAAAATTCTGGGAATGAAAATATATATCGCCGGGTGATAAAAACGAAATTTTCGGCGATATTTCGACGAAAATTTCGATTTTTTCTTCCTTGCTAACAACCATATAAGTTAGAGATCACTCCACAAATATAGGCTTAGCTCACATGAGAAAAGAGTGAGAAATTTAAGCCCAAAGCCCCATCTCTCTAGTCCACAGTCCACACAATTGCAACCTAGATAGCCAAGGCCCTAAGCCACTTCTCCTCCTTTAGCCCAGTCAACCAGACCCGAGATGACTGTCGTTGACTGTTGGATCAGTCAGGCAATCGTCTAGAACCAAGACCCATAATGCTGCCACCGTAAAGAAATCTCAATTCAGTTTTACCTCCTTAGACCCAAGCAAGAGCCATACCGCCGCAAAGCAACCTGACCCAATGCCGTAATCACCAACGTCAAAAGACCAAAACCCAACTACAGAAGCTCCTCCGCCTCTGACGTCGAAAACTCTGCCAAAATTCGCCGTGTTAAATCCTGTACAAAAGGAAATGTGGTCAGGGGCGGATCCTAGAATCCCAACCAGGTGGGACTTGGAATTTTGGTAGGAAAATTTTTTTGACAGTGCTAGAGCAATTTATAGATTGAAAAGAAAGATAAATTTAGACAAGAGGGATTTAGTGGTCCTTTAACACTCCAATGCTTGTTTAATTTTTAAAACTCTCTAGACGTTGCACAAAAAAGCAAAATAAGCATTTCAATAAGACTGACAACCTCACCGATGCTGCGTGAAAATGGTTTGGCAAGGAAGAAAAGAAGACAACAAACAAAAGAGACATATATCTTTTATGAGATCGAAGGTGAGAAATTTATCCCTTTCTTTAACAAAAACAAAGTTAATTATACGTTAGACACTTGGAACACGTCAGTGCTAGTTAGGACTTAATGAGACTCTCCCAAAAAATATATGATTAGTAATTGTACAAGATGAAGGAATGAATAAGAGCTCAACAATCTTGCAATCACACCATAAAATTAGTGATAACTATTAGTAATTTTTTTTACCCAAAATATTTGGGTGGGACTTGAGTCCCATGTTGCTTGCACCAAGATCCGCCCTGAAAGGGGTGTGCAATCCAAAAACTGTTAACCACTCAGCCGCCACAACAACGACTCGAGACCCAACTCCGGAGCGTCCTATACGCAACCGAAGGGCCACATGGCCGTCACCGCTCTTCTACGCGAAACGGTCTAGGTATTCCGAAAAAACGGAAGACTAAAAAATCATGAGTTTTTTTTGTTTGTTTGATACTTGTAACAAATTTGTTAGAGATAACCGCTATTGTTTATGCATAAAGGAATCATTTAACAATTTTAATATTTGTTTATTTTTCTTACAATTAAAATCCACAGGATCCTATGAATATTTTAAATTTGTGGTACAATGGCCACCGACTCACTGCCAAGTGTATCCATGCAACGCCCAAGCCTTGCAAAGCAAAATTTACACCATCCATGGCTTATGGCCGAGTAATCATTCAAAAGCTGCCGTTGAATACTGCATCGGATCTCTATTTCGGTATCCGGTACTTTCCTTTAACTTACTCTAACCAAAATCCTTGTGAATGATCACTCGATCCTTTTCTTTTTGCTCGAACAAAAGTTTTTTTTTTTTTTTTTTTTTTTTTTTTTTTTTTTTttttttttttttttttttttttttttttttttttttttttTTTTTTTTTTTTGCATTGAGGTCAAACTTTAACTGATAATATAGCACATGGTGCATAACACACGCTTTCTTCTTTCATTTTTCGAAAGTAACGCACTCTTAAATAAAAAAGCAACATTTAATTCAATAAAAATAAATTTCTTAGACAAATTACCTCGTCTTGAATATAAGCAAAATGAAGCTCGTCACAGACAACTAGTCCTCAGAGACATTTCTTTTGAAACTCGCCCAGAGCTCGACGGCAGAGTCAATTCAGAGATGGGGAATCAACTTTATCGTCGCCGTCAATTGAATTGTTTCTACTAGATATACAAGAAAGGAGTGAAAGAACAAAACAAGATCACTATAATTTATCGAGAAATGAGAAGTGATGATTGATAAAGTTAAGTTGTGAGAGATCAGAGAAGTATCTATCAGATGAAAGAGAGAGGTTTGGAGAAGAGAAAAAAAAAAGAAAAGAAAAGGAAGAAGAAAAGAATCAGACTTGAGGGTAGTTTGGGAAGTGAAAACAAATTTCATATAAAATATCATTAAAAAAACATAAGGGTGCGTGGTTTGTAAATAAGGGTGTGCAAATTTCTAGATCACTCTCTAGATGAGGAACTCTATCTCTAGATCTAAATGAGAAACTCTCTAGATCACTCATTAGTACTCTCCCATTTAATGGTGCTTCACAAGTTTTTCTATAACTCATGACTTTACGCCTTCATCGTAACTCCAAAACCATCCATAAACTTAGTGTAACTTAAGAGCTAATACCTTGTGTAACTTAAGAGTTACTAACTCCTGAAACCACCAACTCATGAATCTTTAGCATTTCTTTTCATAAGTTATTGTGAGTTATTGGTCTAGACATTTCAACACCAACAACTCCAATTATGATTGCAACTAAGAAAGTGACCAGAGGCTCTGGTTGTGAATCTGTGATTTGGGCAAGAGAGGGAGAGTCAAAGCTCATCACAGACAGCTGGTCCTCAGCGACATTTCTTCTGAAACTTGCCCAGAGCTCGGCGGCAGAGTCAATTCAGATATGGGGAATCAACTTTATTGTCGCAGTCAATTGAATTGTTTCTACTAGATATACAAGAAAGGAGTGAAAGACCGAAACAAGATCACTATAATTTATCAAGAGATGAGATTAGAGGTGGCAAACGGGCCGACCCAGCCCGCCTAATCGGGTTTCGGGCTGTGTTTGGGCCGACCCACGACAGACCACTTCTTAACGGGATCAGGCTTGGGTCGGCCCATTTACTCAAATGTAAGGCCCAGCCCGACCCACGGCACAAGCCCATAACGTGGTGGGCCGTGCTCGGGCCTGGCTGGCCCAAGCCCATGAACGGGCCCTGTCGGGCAAGTTTCGTGCCGAAAATTGAAAATAGTCGAGGTGAATGTCCTATACCACCGTTTCTACTCTCATAATCTCAACTTTTAATTATACATGATACGATTACAAACCAAAACTAATTCACATAATTTATCAATTATTTTTTTCTTTGTCAACGACTAAAGAAGTGATGCGTATTGTTCACAATTAATAAATTTTAGGCAAAAACTTCTTACGAGTCTAAGGGAATCTAAATTCATACTCAAATAAATAATTATTAGTATATAATTTTATTGTTCACACTCAAAACTAAGTTTATTATTTATAAATATGTTATCTTCATATTAGAAATGGAAAAAAAATTATATTTTATAGCGGGCTGTGCTGTGGGCCTCAAATTATTGTGCTCAGGCCGGGCCAGGCCCATGGACACATTTTTCTAGGCCCAACCCGACCCATTAGAATAATGTGCATGGGCCGGGCCGGGCCGCTTTTAAATGTGCCGTGCTCGTGCTTAGCAGCCCGTTTGCCACCTCTAGATGAGATGTGATGATTGATAGAGTTAATTTGTGAGAGATCATATAGGTATCAATCAGATGAAAGAGAGAGGCTTGGAGAAGAGAAGAAAAAGAAAAGAAAAAAAGAAGAAAAGAATCTGACTTGAGGGAAGTTTGGGAAGTGAAAACAAATGTCATATAAAATATCATTAAAAAATATAAGGGTGTGTGGTTTGTAAATAAGGGTGGGCAAATTTTTAGATCACTCATTAGTGCTCTCCCATTTAATGGTGCTTCATAAGTCTTTCTATAACTCATGACTTTATGTCTTCATTGGAACTCCAAAACCATCCATTAACCTTGTGTAACTTAAGAGTTTATACCTTGTGTAACTTAAGAGTTACTAACTCCTGAAACCACCAACTAATGAACCTTTAGCATTTCTTTTCATAAGTTATTATGAGTTATTGGTCTGGACATTTCAACACCAACAACTCCAATTATGATCGCGACCATGCAAGAAAGAATTTGTGATTTGGGCAGAAGAGGGAGAGTCGAAGCTCATCACAAACAGCTGGTCATCAAAGACATTTCTTCTGAAACTCGCCCAAAGCTCGGTGGCAGAGTCAATTCAGAGATGGGGAATCAACTTTATTGTCGCGATCATTAATTGAATTGTTTCTACTGGATATACAAGAAAGGAGTGAAAGAACGAAACAAGATCACTATAATTTATCGAGAGATGAGATGTGATGATTGATAGAGTTAAGTTGTGAGAGATCAGAGAGGTATCGATCAGATGAAAGAGAGAGGCTTGGAGAAGAGAAGAAAAAGAAAAGAAAAAAAGAAGAAAAGAATATGACTTGAGGGAAGTTTGGGAAGTGAAAACAAATGTCATATAAAGAGTGCGGCTATTGAGACCTCCAAATTTGTTTAATATACCTCTCATTCTCTCTACACCTCCCTTTTACTTTTCAATATAAACATTTTCTCAGTAGATCTCATTTCAAATTCCTAACATAACCTTAGTTATTTATTGACATATATTTCGATCAATTAATACTTACTTTACCTCTTATTCACTCAATAGACCTCTCATTCTTTTTTTTCTTTTNNNNNNNNNNNNNNNNNNNNNNNNNNNNNNNNNNNNNNNNNNNNNNNNNNNNNNNNNNNNNNNNNNNNNNNNNNNNNNNNNNNNNNNNNNNNNNNNNNNNNNNNNNNNNNNNNNNNNNNNNNNNNNNNNNNNNNNNNNNNNNNNNNNNNNNNNNNNNNNNNNNNNNNNNNNNNNNNNNNNNNNNNNNNNNNNNNNNNNNNNGAAACTTTTATGAATTCAAGAAATCTTAGGCTCATGGATTCAAGTCTAACGTTGGTGATTTCAACGTGATAGTTCCGCTATATCAATTGGTAATAAGAGTAGGATTTTTCCCGGTGGTTATGGACTCGTAGTCATAGGTGATCAAGTTCCTGTTACCCGAGCAGAGTTGAATGCTCAGAATAAGCTTCTGACTGAGATGGCTGAACAAATGATAGAAATTTGAGAGTTGTTATGTGGTTTCAACAGTAACAACATTCGGAAAAGAGGTGGAGGAAGAGAATCAAATAGGCCGAACAAGGTTGTTAATCATGATTTCATAACCATAGTGAATATTCCTTATTTTTCTGGTCACATTCTTGTGGAGAATTTTCTAGATTGGTTGGTTACGGTTGATAAATTCATTGATATCATGAAGGTTCTAGTTCACAAGCAAGTCAAGAAATTGCTGATGTTGACATGGTTAATAGTGTTAAGAAAACGTTGCAGCCACAAATAAATCAATCAAAACCAATTATCTGTAATATTCTTAAAAGCCAAGAAGAATCTTTCGAAGATGCATATAACAAACTCATCTTTGGCGTTAGGTTCATGGTTGTGCTGTCCGAGTTTGCAAAAAATCAGGCTTACAAACCACATATTCAATTGTTTAACTTATTGAGTTTCTCTTCTACAAATTATTGTTTTTCATTCAAGATGAACTCGAGGTCGAGTTCTTTCGTAGTAGAGGTAGTACGGGGCTAAATTTAGATAGAAAAAAAAGGAAGCGGCGAATAAACAAAAAAAATAAGATCAGTAATTGTCGTGTCAAGAGGTTGCCACCCGTTGGAATATTTAAGAAGATGTTGTGAAATCTGATTTTCACAAGCGAATTTGATTGATAGTAACTTTTTGTCAAAATGTCCGTTTGGAATGCATGATACCTCTTTGCTTGTAATGGGAACAATGAGATCTACAGGACTCAGTTTAGTGATCCTTATCATATTGAAGCCAGAAGATATCGTCTGATTCGTGATTTAGTATTTTTAGGCTAGTTTTATTTCTCTTTTAGGATTATTTTATTTAGGGTTATTTGTTTCCTTTTCAATCTGGATTCTTAGGTTTCATTGTTAGAGTATGATTGTGAATTATATGGATGCCTATATAAGCTTCATCATGCTTTGTTTTAGGATGAAACTTTTGAGTTTTCTCAAATTCTTTTAGTAAGGATTCATATTCTCTCATGGATTCGAAAAACCCTAAGCTTATGGATTCAAGTCTGTTGTTGATGGATTCTAACGTGATACTTCCACTGCATCATACAATGGCATTGTGGTAAAGAGAGAATACCAATATGATGTTTAGAATGAGATGCAAACCTATTGAGATGTCAGATTAAAGGTGTGATACAAAGAATGAAAATGTAAGGGGATAAAGGGTCTCCTTGACAAAGACCCCATAAAGGCCAGAAAGGATTGTGGGGGTTTACCATTGATTAAAATAAGCTTAATCCGATGTAGAGATGCAGTTTAAAATAAGCTTAATCCGGTGTTGATGAAGCCAAAATCTAGATAGACAAAGTACATTGGATGTAATTCCAACCCATGAAATCATAAGCTTTTTCGAGATCTAACTTAAGAGCCATAAAACCAGTGTGATTTTTAGATTTGTTGAAGTTGGAAAACGTTTCATGAGCGATAAGAATATTGTCAAAGATAGATTGTTTTGGTACAGAGACACCTTGGTTATTAGAAGTACATTTTTGAAGAAGCAGCCGTAACCGATTCATCATGATCTTAGAGATAATTTTGTAAGTGACATTGCAAAGACTAATAGGACGGTGGTGATTTACAACCAAGAGATCCAAGTTCTTGGGGATCAAAACAATATTGGTATGATTGGGAAGAGCCAATGAAGTATTATGTTGGAAAAGTCATGTACACCAGGAGAAATTTTATTAAGACATTCCTGATTATTAGATATACATCTCATACTTAATACATCACTAATTAACTTTATTTTTCTAAATTTTACACACAACACATATCCGAACTTCTTACAATACCCTCATTTAAAATACACAATTTATTCACTAAATACAATATTTTTCTAGATCTGCTACTCAATTATTAAAAAAAAAAAATCATATGAGTGAGATTTTTCTCAAAAGGTTTCAGCTTTCCTCATAGTAAAATCTAAATTTCCAACTTCAAATTGTACTATTTAATCAAACAAAAAACCCTAAAATCTAAATTTTGTAGTACATCAAACTTAATCAAAAACAAAGAAGTCATATCTCGCCTAGGGGTTGAGAAATACTACTATAATCTATTACGTTGTTCTTAGTTTTTGCTTGAAACTATGATTGTTCATGATAAATGTCGCTAGATATTTTTGATTAGGGCTTAGTATCTTCATACACGTTGTTCTCGAAGAGGATAAGAAAACTCTTAAACTAGATATTTTTTTTCAACTAGATTTCTGTACTTTTGATATGAATGTCTCTTTTGGTATTTTCACATACTCATAAAGTCTATACAATTTGTATAAAAAAGTTATACAATCTGTAAAAAAAAAAGCTGTGTATCTAGCGTTTGGGGATGTGTTAATAAAATTTCTTCAGGAATAATTACAGTCTTAATATCATTTCAGTGAGTATGATAAAAAAAAAAAAAAAACAAAAAGAGTTGGGAGTCCATCTAACCTGGAGCTTTTAAGGGATCGATGTTGGACACTGCAGAAAAATTTCTTCTTCACATATATCAGAATTAGTAATGATAGGTGAGATGGGTTCAAGAAAGGTGTTTATGTGGTTAGAAGCAGGGGTAAAACTAGGAGTATAACGTTGTTAGCGTGAGTTAAATGGTTTACAATATATATCAAACATTGATGACAAGAAAATAAGTTTCATATTTTCTTAATCTGGCTCCGCCACCAGCTAGGCTCAATGAGAGTATTACTTTAATCATCTTGAAGAGTAGTGATCTGCTTTCGCTTCCTTCGAATAAGAGCTTTAGTCTGGAAGTAGGATGTATATCCTAAACTCATTCCTTATTTTCAATTGCATTTTTAAGAGGTCGTGAGCATATTGTTTTGTGTGGGAGAGCAATACTCAAGTGAGTCAAATCTTCTATTTATTTCAGTTTTGAAATTTCTGTCCATTGCATGAATGCTCAGGCTCCTCCATTGGACACCAGACTGAAGATCTCTTGGCCTAACCTAGAAGCACGAAAGACTGATGCAGGCTTTTGGGCAGGAGAGTGGCATAAACATGGCAAGTGTTCGGAGCAGACACTTCCACAACACGATTACTTCCAGCGAGCCCATGACATTTGGGTGCAGCATAATATTACTGATATCCTCCAACAAGCAGGCATCGGATCGAGGACAACAAAAAAGTACACAGAGATAGAATCACCCATATTTTTAAAAACTCAAAAGATACCCCTCCTTCGCTGCAAAAATACTCGGAAGGGGCAGATTCCGTGGTTGCATGAAGTGGTTCTTTGCTGGGACCATGATGCAACATATATGACCGACTGTCATAGAAAAGAAACAAATTGCGGAAATAATGATATCAATATTCTATAGACAATATATATATATCCTCAGTCAATATTCTATAAACAAACAGAGAGAAGACACATATATATATAACTCCATGGTATTGTGGTTAAAATTTTAAACTTCCTATTATGTCCATGATTTACTGAAGAACAATATTTTAATGAAGAGGGTAAAACTGATTTTAGTCAGAATACTATCCATTGATATACAATTAGACTGGTAGTAAACAATTTTTTTTTTTTTTTTTTTTTTTAGGGCAATGGAGTCAAGCTCCGATCCGTTTATTGAAATATGACGGCATTCTGCCAAATTAAGGAACGAGTACATCATAATACAAATGTGAGGCTCAAAAATATGCAAAGGCAAAAAACATATACTACCCACAATTATCGAGTGCAATCGTGATTTTCACTAGCAAAAATAACCTAGAGAAAATAAAACAGAAATAGAAATTTTTTTCACAAAATACCTTTTTTATCAAAATGTCATACCGGAATACTATGTCGTTAGACCAAATGGTACTAGTAGACACTTAATTCTTTTTCTTTCAATTTTTATATTTGAAGATGTATACAAAACTTTTACCCCTAATAATTCCGACCTATTTCTTCCCTATCAGTTGACAGTTCTAGGGTCTAAAACCTAAAGGTATCCATACAATGATATCTTCACAGCACTTTACTTTAGAATTTTTTTTTTTGTTGTTGAGAAATTCGGAATTACTCCATTAAGCTTTAAGCCAATCATGACCGATAATGGTTAATATATACATGAGCAATACAAAGATTACCCAAGCACAAAGCAAAGTACTTAATCAAAGCTATATCTACGAAATGAAAAACAACTGCAAAACTACGTCAAAAAAGGTGGACATAAAACTAGAAATCATAAATCGACCTTACCCCCAACATAACCAATCTCTCAACGACTAGAGGCCTAAGACCTATTGGTGTACCTTTCATCGTTTAACTGGAGAGAGAAGTTACGCTTAGGGTTTGGTAGGGATCTTGTTTGAAGCCCACAACAAGCTGATTCAGACCCAAAACAGATTGAAAAGAGAAAGAGAGAGGTGAGACCCAAAGGCTTAGCCCACTATACTACAAGTAGCAGCCCAACAGACCAAGGCCCAAAGGCCCAACCTGCTCCTCCTCTCTCCTATCCAATTCGAACCTGACGGCGAGCTTGCTGGAGACTCCTCCAACCATCGTCTCAATCCGGTGGTTCTTCAGCGCTGGGAACCATAAGCATCGCTACCTTTGAAAATCCACCGCTAAAAGCCTGGAGCACTTCTTCGCCTTGAAGCTAGGATTTGTCAGTTCACGCTCTCTCTCTCTCTCTTTCTCTCTCTCTCTTCGAGCAGCAAACGCCGACGCTAAGACCTGCCTATCAATGCCTATACAGCCACTTTGTTGCCACCTCCTTGCTCAATAACACTTGCCCAGACTCGATTTGCTTTCTCAAATTCGTCGTCGAAAACATGTTGTGTTTTTGGCTTGTTGATATGATTTGGAAATTAAGGATTCAACAGGAAAATTTTAGTCCAGAAAGGAAAGAGGATTTTCTATAAGTTTTAGGAAAGTAATCTTTCAAGATCTATTAGGATTTTGTCTGCTAGGTTTAGGAGTCCTAAAATCTTATTATATCAGCAGCCCTTATAATAGGGAGCTGCAGGGGGATTCTCTAAGCATGAGACAGAAAACCTAGAGAGTCAAGAGTGATAGAGAGATAAGAGATCTAGCAGGGAGTTGGGGATTAGCATAGGGATTTCAATAAGTACTTGTAATCAAGTTGTTATTCTCCATAGTGCTAGTGATTCTCTCAAGAGAGATTACAAAGTGGACGTAGGCTAATTATAACTGAACCACTATAATTCCGATGTGTTTGTTTTTGTGCTCATTTGTTTTATCTTTGCCTATATCGTAAAGTCTGAGCAAAGATCTTAGGGACTTAACCACTATAAGTGGTACCAGAGCCTGGTCTTAGAGTACTTTGCGATGATGAAAAGTAAAGGGATGGTCAAAGCAGGAGATGTGAAAGTGTCCATCAAACCCTTTGATGGCAAGGATGATTTCACGCTTTGGCAGAGGAAGATGAAGAATGTCCTGATTCAGCAAGAGACCAACGAGGCTATTGGAACAAAGCCTACAAACATGCCTGACGCAGAATGGACGAAGAAGGACAAGAAAGCAAAGAGTTGTATTGAGTTGCATCTTGCCGATAATGTCTTACTTCATATTGGAGAGACAATGACTGCAAAGGAAGCGTGGGAAAAGCTTGAAAGCGTTTACAGGGGTAAAAACATCAGCAACAAGCTACTCTTGAAGGAACAGCTCTTTGGCCTCAGGATGGAGGATGATCTTAATGATCATATATGTAAGGTCCAGAATTGCATAGAAAATTTGGAGAAGGTCAGAGCGAAGATGGATGACGAAGACACGGCTGTTATGCTACTGCATTCGCTGCCACCTTCATTCAAGCACTTCAAAACCACCATGATATTCAAGGAGTCAATCACACTCTCTAAAGTGTGTGAGAACCTGGAATCTTACATCAGATTGGAGAGAGAAGAAGATAGTTCTCAAGCACGGGGACTCTATGTCAGAGGCAAGGAGAGAGGAAGGTCAAGGAATAGAGGTGGTGGATTTCAAGGAAGGGGCAGGTCTAAATCCAAGGGAAAAGGCAAAGAAAAGAAAGATGGTTGCTTCATATGTGGTTCACCCGACCACTGGAAAAGGAATTGCAAACAATGGAAGGAGAAGAAGGCGCATATGTCAGGAGAAAGCTCCTAGTCAGCCAACATTGTTATTGGAAACAATGATGAGGATGGAGAACTCCTGGCAATCTCAACCAGTTTCAGCGCGCCTAGACATTGGACCTTGGATACGGCCTGCACGTTTCATATGTGTGCACATAGAGATTGGTTCGACACATATAAGGAGAGGAACACCGGATCAGTTTTGATGGGGAATGATTCACCAAGCAGAATTATGGGAATCAGAACAGTAAAGATCAAAATGCATGATGGCATTGTCAAAATGCTGGGAAACGTTAGACATATCCCAGGTTTGAGTAGAAATCTGATATCATTAAGTATTATGGATAGGGCTGGGTTTTGCCATAAAAGCCAGAATGGAGTGCTCAAGGTAGGAAAGGGACAAATGGTCTACATGAAGGGTGTAATACAACCAGATAACATGTAAAAACTCACAAGTTCTACATTAGAAGGTGGAGCAGCAGTTTGTACGAAAGAAGACAAGGCTGAGCTTTGGCATAGAAGGCTAGGGCACATGAGTTAAAGAGGGTTGCAAGAATTACACAAGAAAGATCAGTTAGATGGTGTAATGAGCAGTGCTTTAGAATTTTGTAAGTATTATTTCACATTTGGGAAGCAGACCAGAGTGTCATTCAATGTGAGCAGCAACGAGAACAAATCCAAAGGAGTACTGGACTATATTCACACAAATGTCTGGGGGGCCAACAACAACAATATCAAAGGGTGGAGCTAGATACTTCGTGTCCTTGATAGATGATTTCTCAAGGAAGGTCTGGATTTTCTTTATGAAGACTAAAGATGAGGTTTTCACCACCAAGTTGAAGGAGTGGAAAGCTGAAGTAGAAAATCAGAGGGGCAGAAAAATCAAATGTTTGAGGAGCGACAATGGAGGTGAATATAGAGATAAGAAGTTCTTACAGTTGTGCAAAGATGAAGGCATCCCAAAGCACTTTACTGTTAAGAAGACTCCCAACAAGATGATGTTGCAGAAAGGATGAATAGACTCTCATGGAAAAAGAAAGGAGCATGAGATTTCATGCAAGGTTGCCTGAAGAGTTTTAGGCAGAAACGGCTAATCATGCATGCTATTTGAATAACCGATCACCTTCTATGGCTATTAATTTCAAGCGTGCAGAGGAGGTATGATTTGGAAAACCAGTTGATTATTCCAACTTAAGGGTGTTTGGTTGCAGTGCTTATGCACATATTCCCAAAGACGAAAGAACTAAGCTGGAACATAAGTCCCTTGAATGCTTGTTCATCGGCTTCGAGAAAGGAGTAAAATGCATGCTATTTGATTAACCGCTCACCTTTTAGGGCTATTAATTTTAACTGTGTAGAAGAGGTATGGTCTGGAAAACCAGTTGATTATTCCAACTTAAGAGTGTTGGTTGCAGTGCTTATGCACATATTCCCAAAGACGAAAGAACCAAGTTGGAACCTAAGTCCCTTGAATGCTTGTTCATCGGCTTCGAGAAAGGAGTAAAAGGTTATAAGTTGTGGGATATAGTCAACTAGAAAAAGGTGATTACTAGAGACGTTGTCGTTGATGAAAAGACCATGCCATTAAATGAAGAAGCTAATAGCAATAAGGGGCAGATGGCTATTGATGAAGGAGAAGCAATTAGTATTTCATTAACTAAACCATCAGTCGCAGATTCCGAAGCTCAGGTGGAGCAAATTGAGCAAGGAAATGATAAAGTAGCTATTGAGGAATCAGAGCATTAACAACAGCCAACCGTTATGGCTCAGGTGGAGCAATCTCCACAAAGAGGACAAAATTCTCTTATCCCATATAAGCACCTGAGTCATTCAAAAGAAGCATAGCACTTGACAAACCTAAAAGGAATCGAAAACCAATTTAGCGGTTTAGATTCGAACTAGAAGAAGATGTGAGTCATGCTCTAAGTATAAGTCAAGGAGATCC

**Supplementary Sequence S2. The DNA reference sequences of *S_b_*-RNase.**

CTCCTTAAACTCTTAAAGTAACGATGTTTTTAGTTCTAGCATGTATTTTGATAATCAAAACCACCCATTCCCTATCAAATTTTTATATCTATACCGTTCAATTCATTGGGTTATATGAGTAGATCACTTATAAAAATTTTCAGATAATTTGTTGATCTTTAAGTCATTCAAAACTTCGATTTATTTTTTATGATCTTGAATGGTCCAGGTTTGATATAAAATGTGTAACCTTTTGTTTTAATCTTAGTCATTCAAGCTCATTAAGTAACAGATCTAAAGATGTCGGTCTATCTCTAAAAATAGTCAACCCTGATATATATATATATATATAATTAAGTGACCATCACATACTTAGAAGTAATCAACGATTTTGTAGTCTTTTTTCCTGTCAGCAGAGAGGAATCTTTTTTAGGAGACATTCTTCGTTTTCTTTCCAACCTCTGACTTCTCAAGTACGGATAACTCGCCTCTCCTCTCATACATCTTATATAAACAGCACATGATGCATGTGATCCTTAGCGATCACAAACTTTTCCCCTCTTCTCTTTATGCTGCCTATCTATACAGAGTATGGCAATGTCGAGATCATCAATCACTCTGATTGTTCTTGCATTTGCTCTCTGTTTTACAATGAGCATTGGTGGGTTTGTCTGCTTTATGGTATTACATATACATATACAGTTTTTTCTTTGAGTGGTTTTAAATACACATCTCAAATAACTTAATATATATATATATATCTTTATTATTTGATATTTCAAATCAGATTTAATTGGTATGTTAAATGATCAAAAGTGGCATATGTAATACAATTTAAATATTAAAAACAAACTTATATTTGGAAAATAATAATATGAAATATAAACCAAATTAAAATATTAAAAACGAAAGCTTATTTTTGGAAACTTATAAAGTGAATTAGAACAAATCAAATTTTTAAGATCGAGAACTTATTTAGGGAAACTATGTAAACAAAAATTTGCAAGGAAATTTAGATAATCAATTAATTTCTTTTTTGTTTTTAAACATTCATAATTTCCAAGTTTAGAGTGTCAAAGTGTCAAACGATGTAATTTTTATGCATATGAACACCTATTTCGTTATATTTCACATATTTTTCTTTTCACCCAAACACACTATTCAGATATGTATTTTTCATCTTGTTTATCATTTTCATGTTTAAGAATTTGAAATAAACAATGAATTTGTAAGGATTTCTGAAGAACTAACACAAGTAATAGATATGAACATCTATAACATGTTAATTTATATAAATATATACTAATTATACAATAATAATAGTGTTAATATAGTCACATAATATGATATTTCATGAATTTTTATATTAAGGGTATTTTAGGTACTTTAGGTTGTGTATTTAGTAAAATATCCGAATAAGAAATTAAATAAATAGTGTGTTGAGAAAGAGATGTGTATTAAGTTATTTGGGGTGTGTATATAAACTTTCTTTTTTTCTTTTTCTTTTTGAGAAGAATTATGTATACATTTAAATTCTTCTAAAAAAATGTATATACATTTAAATTACCACCTTTATTTGTTTTTTTTTTTTCGGAGGAAGTCCTTTATTTGCTTTTAAAATCTAGCAAGTATCTGAATTTTCATACTACTTTTAGATCATGGTTTGTTTTATGACGCGATAGTTGTTGAAAGAAGTGCAAAGACATTAGGTAGATAATACTTAATTAGATACAATCTTGCTATAGATTTGATGTCTTACTAAACTACACCCACAAAGAGCGCTTCCATGTATTGTGGGTATATCCCAGTTAGACCTCCAAAAACCCTAAGCCTCCACTTTTGTCAAAACACTCGTCCGGCGGTGACCGGATCTGTGATGGCCTTTGGCCTCGCTGGTCTCGGGGGTGCGCCGGACAAGTATCCTATTTGATTTCAATCACGGAGTAAGTCCGATATGAGCAAGGTTGAGGGGTTTCAAGCTTTCATGGTTTCAGAGCAAGGAGGAGTCGGCACAAGCAAGGGTGACTCAGCTAGATCTTGCAGCTAGTATCAATGGTGAGATCCCTTTTTTTTTATGCAGGTTTTTAGGGTGATTTCCATTTGGAGTCCGTCGACTTCCCGTTGCCATCAAATCGGGGATCTATGTTGGTGTGTGTTGTTGAGGGAGAGGGAGGAGTCTCATGGTTATGGGGTTTGAAGGTGGCGAGTTGCGATGTAGCGAGGTTGGCGACAGAGTTCTGGCTGTGGTTGGGATCTGACAACGACAGCAGCGGCACGACGATTTTGCTGGTGGTGAGATTGTTGCTAGTGGCGACTGCAGACTTAAGCCGAGATCATGGGTCAGATATGTTGTCTTGCTTCATTGGTTTGCTCTGGTCGTAATGTCTTGGGCCTAGCATTACTCTAGGTCCAAGATGGTTATCTCTTGTTTTCTAGGCCTTTTTTGGGCCTCTAAGGTTTTAAATTAGTTTTTGCTAATTTATCTCCAATAATTTTTCTATATAGGTTTCTAGGAATTCTAGGTAGATTTTGTGCATGCAGATTATGATGTTTCTTTTACATCTAGATAATGGACACTTCTTGTCGTACCATAATTGAGCGCCTTGATGACGGAAGGCTAGATTGTAACTTGCTAGGCTCATCATCAATGAGAAACAATGTACAATGTGTTGGTATGTGAATCACCTCAATTCATTGGCGTCCGTATCGATGTCATTCTGGCTGCGACTATTAATACAATGGGTTACATGCCCGCCTTTGCTTCAAAAAAAAAATGGATTTGACCTCTTAGAGCATATTTAGCAATGCTAGCTAATTTTAAGTTAAATTTTAGTCAAATTAGCTAAATTGTCATTTTGGCTAGCCACTTTAGAAATGCGGCTGCATCTATGCTCTCTATTTTAGCTAGCATTACATCAACATTATTCTTCAAATAAATATTAAATAGTTTAAATATATTTATATATCACGTAAAATATCTTACAAGGAATGTATTAAATTATAACTAGTCTCATCTGAGTCATCTTTCTCTCTATATTTAGCCAGCGAGATAGCTAAAAGTCATAATAGCTAAACTTTGGCTAGTCTGCTGGAGCTCCGAAAATATAATAAAAAAAATAAAAAAAACTAAACATGCTCTCTAAAATAGTTAAAAGCTAAAATAGAGAGTCTGCTAAAGTTACTCTTATCTACCACATTTGTCAGACGTTAATGTTGAGTGTGGTGCTCATAACGATTTGGATTAATTTCTGTTTAGTTGGAGGCCCGTTTGTATGGTAGGAAATCATGGTATGAAAATAAAATTAGTTTGAGGCCCGTTTGTGTGGTGCTCACTATCCTTACATTTCTTCACAATGCATTATGTATGCAATCGAATATCAATACTAATAACATCCAACATATGCTTTTATGGAAAACGATAACATCAATCATATAACTCAACGATTACACATGTTTTTTGGAAAAACAGTAACATCCAAGAGTTAATCACTCAATCGCACACAAGTCAACGATTTCAATTATAACTATTTATCACTTTAAATAGAGAAGATTCCCTAAGAGACCCTATCTGGAATCGAAGCTGTAGCTCCAACACTAAACTAAATTAGTAACTCGGTGACACACATTCTGAAATCATTATCTTTCTGCTTCTGCGATCTTGTAAGAGCACATACAATTCCTTGTGTCAATTCCTTGTTTCGAGAAAGTTAACAGATCACTTCAAATTGATATGATCATGCCCTTGAACTTTCTAAGGACACCCCAAATCCTTAATCTTTCCAAAACAATTCCAAATTAAACATCTTTCTTAATCAACAACAAGTAACATACTAATTGAGATATCCATCCTTTATTAACAAGATAATACACTAAGCTCCACCTTCTCCCTACAACGTCATCTTCTTTGTTATCAAAGATGAAGTTGTGCACAACACAAGAACTACCATAAGTCCAATTAGGCGATAATTGCCACCTCCACCTTAATTAAGCTCAAAGGACCACCATCAACTTCTACCTTTCTTGAATTCTAACACCACAACTCCACAACACTAACAATCGAATCAACAACTAATAGCAGCTTAGACATTTTTCAGACAACTCCAGTTTCGAACCCAATTCTTCTAAACTACTCTAAACCATCATCTTGTGAATAGTAACTTCACGGTGGTCATCCACTGCCGAAATCCCAACTGCTTCCAATTCTCCTATTCATCTAATTCCAACATATATAACAGTCACAAACATGCTAAGAGATTCATAACTACTGGAAATCATGAAACATAGACGGAAACACTACTGCCGCCATCGATCCACCATGCACAGTAGCCTACGGTCTTCCTCTGCCAAAACCTAACTTCTTCAAAACTCCACCAATATTGAATTCAACATAACAACACCATTCATACTCTCTTCCATTGCTTCTATCAATAACAAAATACACAATTCGAAACCCAAATGCATCACTTACGCCATCGTGCACGGAGGTCACACACGGTGGTCGACACAGCCAAAACCCAACTTCTCCAACACCCAACCAATCTCCATTTCAATTAAACAGAAAGGTAGAGCTCGACAAGTAGATGAAAGATTGATACTTGAATCGAAACTCAACTTGGTTGGGAAACACCAAGAACACGGCCTAAAACCTAAACAAACTTCTTCTCTTCAATTCTCGCTCCTCAAAGCTCGTCTTGGAAAACCAAGACCATAGAAATGTTCCTGAGATCGAGACGAAGCTATCCATACCAGTCATGCTCGTCATCGTGGACGAATTCGGAAACTCGTCGGAGAACCCAAAAATCCTTCCCCTCTTCGATTATCTCTTATTGGACTTCATATGAAGAAACTGAGGCCACAAGAAGAACGTAGAGGTCGAGACAAAGCCTTCGCCACCACTCTTTCTCCGCGTCGTTGTCGGAGGACGAAGCGACAGCTGGGACAGCTCGTCGCCTCCGCTTAGGTCAAGAATGACTTGATCTAGGTATCAATCTGATCTAAATTGATTTGATCCCTAAGAGCAAATGCACCCATGAGGCAAGGGCAATTGCTCATTTGCCAATTAAATTAATACTGCTGATTCATGATTCATCTCACATTAGCAATTGCCTGTTCTTCGTCAATGCACTAATTAAAGGCAATTTACTTGATAATCCACTATTCTCATTACTTGAATAATTCATTAATAATGATTTAATTCAAATGTTTGTTAACTTATTTTAATTTAATTAATAAGTAATGAGATAATTATACAACATTATTTTTTTGCTAAATAATCAATTTTAAAATATAAAATTTCTTCTTCATATTGCCAGTTACTAAAGGCTTCTCCCATTTTTGGATGGTAATTGGAGAAATGAAGAACTAAAATGGTGAGGAAGAAGATCAGGGGTGTTTGGGTATTTATAGAAGAGTTAGGATTATTTATTTATTTTATTTTTCGGGTGTATTTCTGTTTAAAGACAAATAAATTAAAAAGTTATCATAGTTGATCATTAACCAGGCCGTTAGATCGTCAATCTGATGGATACAATTAACTATCCGTTTGAAACTAGAAATTACTGTTGGGGAGAGACGTTGGGAGCAGCTGCACATGGGGTGGGAATTGGTGAGGCGATTCAATGGCCAAAAATTGGTAAGGCAAGTGGGACCTAGAAATTAGTCATACCGATTTTTTATGGGTTTAGGGTGCTTGACATTTCAGTCAATCAGCCACTCAATTAGTATGCTTTTATATATAATCAAATCAACGGTTGAGATCTTACAGTTTATAAAATCCAACGGTCCAGATGCAGCCGACTTTAATTTTTATTTTTAATTAACTTTTTAGATAATACTAACTTTTAAAAATATTAAAAATAACTCCGATGTCCGAAAACGCTTCTATAAACTCCTACGAACTCGTTACAACCTCTACCTTAATCATATGGGCTCGAAAGACAAATTTTAACAAAATAAAAATTTGAATAATTTTCAAAAAAGCTTCTAAAAATTTAAAGAATAATATAAATTGAAATAAAAGTAGCTCAAAATAAATTTTCCTTTCAAAAATACAAAATAAATTATCAAGATGTTACAAATCGCAAGTCTGAGTTACCTCCCAATGCTAGCATCCTACGTGCGTCAAAGGCCCTCTTCCGAGCTCTTTTCGGACCCAAACTGTTCTCAGGTTTAGGCTTATTCTTCGAACCCACTAGACGACTTCCCCTACGTTTCTTGGCAGGCGAAATGGTGAGGGTAGCATGCATATGTGGAATGTTGAATTCTGGAGCAAATGCCAACTCTAGAGGTTGGGTCTCTAGCATCAGACTGTCACAGCGGCTTCGCTTAAAGTTCCTTGTCTTTATATCTGCACCTCTCTTGGTATCTGTGATATTAGCTACCATTTCAATCTCATTGCTTAACTGTACCTGAGCCTCACCAGCATCACCACCAGGTTGGTCTAAAGGGGAGCTTAAACAGACAAAGCTGCACCGCTAGGTCCGCCGTCAAGATCCATGGGAGGGAGAAGGTGAACAAAGCCGACGTGCGCCTAAAGAGATTGGCGACTGCAGGGATATTACTACCTCCATGGGTAGCAGCAACAGGCGAAGCCGCTCCCAGCCCTCCCTTGGCCCTAACCATGATTAGGGTTAGGGTTAGGGTTTTGGGTAGGCGAACTCCTGCATGCCTCTGCAACCGATCCGACTGCACCACCAGAGCCACCGTCAAGCAGCACCACCACAGACAAGACCGATGGTCCCTCACAGCCTGCAGAACCATGTGCAAGCATATCGCACGTCTTGCAAAACCCAAACACTCTCATAACTAAATTTGAGATTAACCGTGGCTCTCAGAGATCCAAAGTCAAATGACATTAGCGGGTAGGCTTCCTTGACAGGATCAGCAATCCGGTGGCGCACTCGTGCGCACTCTCCCCGCCTTATACCAGGCTTATCCATGTCTTCGATCATACCTAAAGTCGATCCAACCATGGTCACTGCCTCTATACTGTACAAAGCTTGCATCAGACCCAAGATCTCAATCCAAACAAGGTAGGTCATAATCGGAACTTGAGATGCCGAGCACAAACCATCATAGTGCGCAACCAAAAAAGGTTCCGGCCAAAGAACCAGGTGCTGTCAAGGATGTGTTGCCGATCTTCTGCAAGATTGAACCGCATAGTAAAGTGACACTACACCATAAACTATCATTGATAGCATTGATAGCACTTGAAGAATGCCACAGAAATCAAAAGATCACCACAATAGAGGAGTTTATTTGATGCATACCATAATTCTTAGGCATAATCATTGTCTGCTGCCCATGCTATTATAGTACCAGGAGGATCTTCAATTCTAACCTCTCAATAAGTGGATCGATTATATTAGCATGCTTCAAGGGAAAAACTTGTAAGTTTTTCACACACCCCTTTGACACCTGCAACAATGCACATTACTCAAAATTTAAAACAAAGAAACATGATCAAAGACCAAAATCTGAGTTAAAGCTCATATGTACATAAATATATGATTAACTACCGTTTCTACAGCAGCTATTTAATTTCATGGTCTTGGACTGGCATTTGTTGCACCAAATGTTCGCCTCAATGGATATCTTTTGCTGCATAAACATATATATGTTATATAGTTAATCACAGAGTTAATCTTTGAGGTCTTTTAGACACGCATAGTGAAAGAGGATGAGAATACAAAGTTGGTTTGGGATCGGAGTAGTAGATTACAATACCTTCATATTCTCCTGAAAAAAATGTGGTTTCTCCAAGAAGAGAGCTAAGGATGTTCTTGCTGCTAATTCTATCAGAATGTAATAAAAACCTAGAAAACTGAGTCTGTCTTTTATAATACAAATGCAAACATATCTTTGCTAACATAACTGACACCAAAGAGGCGCCTCGCTTTTGACACCAAAGAGGCGACTCGCTTTCTAATAGTAGAGATAAAACTCATCACTGATTCTACAGGTACTTCATGTAACAGGGCCGGCCATCTAAATAAGAGTGAAAAAGCATGGTATATATATACTTGGGGATATACTTATATAAGATGACCAATGTCTCCAAAGCATATTTTGAAATTAAACTATAATGGGCACACTGAATTTTATTCTCATATAAATTTTTTATACCTGAATTGTGCCACCCTTCTCCAAGTGTAGGAAGTGCCAACAACCTACTTCCACAAGTCACATTACTATTTTCTCATGTAAATCAAAAACTAGCTATATTAGTTCTACTTGGGATAGATAGCATATATACCAGTTGTTTCTAGTATCCCATACTCGCTTTCCGCATATGTGACCAACAAAATTTACAGATAAAGTCAAGTGGTAACCAACTATTGTCCTCACACCCTGCAAACAAAGCCAAATGATCTAATAATTATATTCATCAAAGAAAAACAAAATAAGTTGATAGTACCACTGCACTGCTCGCAAATTCTCATCCCTTCTATACCAATTCCAAATCAGAGAATTTGGTCATGGGAGATGGTTTTACTGTAACTCCATGCCCTCGCAACTAAAGAATATATATATATATATATATATATATGTATATATATATCAGATGATTAATGTCTCCAAAGCATACTTTGAAACTAAACTATAATGGGCACATAGAATTTGACTCTTACATAATTTTTCATATCTGAATTGCAACTCTCAGCTACTCTGTAACAAACAAAGGAACTTGTAGGAATTTTCTATTTGGCCATTTACCATGACTGAATCCAAAAACTAAGCTCATTAGGATTTTCTGAAATAAGAAACAAGTACAGAAAACCCCTGATCATTTACTAATTTTGAGTGTTTGTCGTTTTAATTGGAGAAAGCTCAATCGAACAAGAACATGGAACGATTCACTAAAAGTAAACAATAACAATTGAAAACCCAACTGGACAAAAGATCCCCAATTGTCTCTAAAGCCTCAATAGTTGAACAACTATAATTAATAATCACCCTCACTCAACCAAATCTGATTACCCATCCACATGAATGCATAGAGGCAAAGAAAGATGATGAGAGAGGTCAGAGAAGAACCTTCACCAGTTAGAGTTTCTGAATTTGAATTACCTTTGTCCGTTTAGGGTTTGAAAATTCGAAACCGCACACCTCACATCTTCGGCAATTGAAGTTCCCACACGAGAAGGACAATTGCCAATTAGGTTTTCCAAACCCGACCTCAGATCTTCAGCCATGGCCTCCACGAGCTTTCGCCTATTAGGATTTCGCGAATAGGGGAAAGAGAGCTCTGAGAATTAGGGAGTGTGGGATTTGCGAGGAGGGGTCGAAGAGTGGACGTGGAGTGGAGCTCGTAACGACGGTTGAGGGAGCGGCGGAGATGGATTGGGAGAGAGATCGAGGTGGAGAGAACAAATGGGAACTGAAGAAGGAGAAGTGATGCGCCTTCATTTTCAACAAGTGTTTTGACTCTTTATTTGGGCGGGCAAGATAAGTGGGAAAGGCGAAATGAAAATTAATTTAGACCCCGCCTTCATTTTCGATAGGAGCGAGACTTTAGATAGCGTTTCCTTTTTCTTTTCTTTTTTTTCATTTTTTTTCTTTTTTTTTTCAGACTAAAATGGTCAATCATATCATAGCACTCAGTGAATAAACGCTATTATTAAGTGCTATCAATGTGCAATATTCTTGTAGTGTGATCTCCTTCTGCTCTCATGGTGAGCCGGTTCTTGAGTACCAAGATCAAAGGCAACGTGCCCATTAGGACCTTAGGGTTTGGTTGTTTTTTTAAAGAGAGTAACCTCCCAACAACAAAATGGTGTGGTGATACCGCGGCGGCTCCAAAGGAAACCTTGCCTCTGTCTTGGACAGCCAGGGCAGCGGCGAATCAATTGGCATCAGAGTTCGACATGTTATTCAAGGATGAAGGGTGCCAAGCGCCATTGAGAGAGAAGACCTAGACACCCTAGCAAAGCTCTTTTCAACGACGCTAACTCAATAGAGTTTTTTTCCTAATTTCATGTTAGGTTTAATGAGGCCACTAAAAACTCCAACAATGTACTATTATTGTTGAAGAAATAAATCGTTTTGATAAAAATATATATATATATATATATATATATTTTTTCTTAACTAAAGAGTGAAATGAAGTTTAACATCTAACCTAAAATAAATTGGGAAAATGCCCATTACATCAAAAACAGCTAATTAATAACCACTCAATGCTTAGATAGGTATTCTTTTACTGTGTGTTTGGATGCGGGTGAAATAATCGTGGAATTTGGGAGCTTAAATTCTCGACTTGAATTCCTCATAAAATAGGCATAATTTGTCAATCCTCTCAACTGAGGTTAGTCTAAATACTGATTCTTGTTATTTTAAAATTCTACATCATGAAATCCTAACAATAAAATTCTCAATCAACATGGAAACGACGACCGCAATGAAACAACATTGAGGACCAACGCCCATCGCTCAATATGGTCAGATCTTGTCAGGCCTTGATCACCTGCAACAGTCTCTTTCATATGCTTTTTAGGTGGTTTGAGTCCCCGTTCCAGAGAGATGTTCACTCTTCAGGCATTGAATTATGGTGAGTCATGGAACTAGTCATCCATCACCAACCTAAGTCCTAAGAGATAGGAGCCGGGAGAGGAAGCAGAATAGTGCTTGTTCTTAGAGGCCAAAAGATGACATCAATTGGGCGGTGCACGATCCTGTCTTCAAAAGAGGAAGAACCGGCGCTGTTAGAGGGGTTGGGTTGCGAAAGGATTCGCTTGGAATAATCTTGCTGACGACGGCTAGGGTCTAGAGAGAGTTTCAAGTTTCCTCAACGTTTGGATAAACCAATTCAAATTTCTATAAAAATTTCAAAGTCTTAGGAATTAAAATTTCATGAATTTAAATTCTATTGATTGAGAATTTTATTGTTTGGATTTCATGATGGTGTTGTATGCGGCCAAAGCGGCGTAGTCTTCCTTGCCGGCAACGAGAAGCAAGATGGATTGCTTCCATGTATTGAGCTTGGGCTTGGGCCCAATGGGTGGGTTGTTGACCTTTTCAGGCATTGTTTGGGCCTGGAGCCCTGAAGATTGATGGACTTCCTATCCTCCTGTTATTGCGGTCGTAGCATTTTTTGCGTTTCTATACCTTTTTTCTCTTTTAATTAGTTGCCCTACTTTTCACTGTTTAATTTTTATTTTACAGCTTACCAACTGTGCTATTGATCATAGATGTAAGCTTACTTTCATTAGTGAGCCTAAAATTTCGAATATTGGTAGTCTATGCTATATATCACTATAATTATAGGATTGCGAAGCCTTACATATCGTAAGTGGTAGCGGTAATGGTTATCTTGACATGAGTTTAATAAAATAATTATCATTTTCTAAACAAAAACTTCTATTAGGGGTACGATCGAGTAGTCCATATTCCCCTCAATACTATAAAAAATGCTCTCTCTCTAGCCTCTCTCAGGAGAGGAAACTTGGCGGTGGTGGTTCTCCACGGGTGGCAGCCTTGCACTTTCGATTGACAGGTGGATCTCTTCCCTCTCTTTCTTGTCAAATCGGTGGTGATGATGGCGGGCTTGCACGACGCCATTCCATGAACAAGATTCAATCAGAATTTGATCTGATCTTGGATTCGGATGGTTTGGGAATGATGAAGGGGATCGGTGTGCTTGGCGGTATCATGTCTGGCGGCAGTTGGGTGCTTTTTCTGGTCGGTGGGTTTCAGATCTTACCTTGCGGTTGTACACCTGTTCTTCCTTCTCTTTGTGCTATTGAGGAGAGTGGTGGTGCTGTGGAAGGAGTCAGCGACGGTGATGCGGCTCTGATTCAGAAAATGGCTACGTCGGCGTTGTTGGCCTCGGGTGGTGATGACGATGGCAAAGGGGTTATGGATGCTAAGGTTTCTTTCTTGTCTCCTTCAGGATTTGGGTTCGAATGGACTTAGGTTGGAGTTGCTAGGGTTTCCTTAGCTAGGCCTTAACATATGCAGTTGGGCTTGGACCCCTAGTTTAATCCAGGTAAACTAGTTTGCATTTGTATAGTATCGATAGTTTTTTACGGATCCTAGTTTGTTTGTAAAATCGTGCTTAATCACTATAAACTAGGTGTACTTAGGATTTTATAGCTTATGTTTTAGCTAGTGTAGGATTTGCAATTTTACAAGATGGTGCTTTCTGATGACCCCTAAAGGTGCGTCGTCTTTGTACTGTTTATTCTGATTTAAATGAAATGACTTATTGATAAAAAAAATGATTATCGTTTTCTTCAACAACAAAAAAATGCAGCGCCTAGATTTTAGAGAACAACGCCTAGACATTTATGTTTAAATTATTTTGTTTTTCTTTCAATAGATTAAAATTTGTTGCCTAATATAATTCGGTTCAGTGACATAATCTTCTATTTATAAGTGAGAAGTTTTGTCTAAAAATAAAGTACTTGTAGCAATTAAATTTTTTATGCGATCAAAATTATATGTATTGTTTCTCATGATCTTGTCACGATTTATTTCCATATAGGATCCTATCAATATTTCAAGTTTGTTGTGCAATGGCCACCAGCCCACTGCAGATTTTTTAAATGCCGCTATAGTAACCCGCCACAGATTTACACCATCCATGGGCTATGGCCAAGTAATCGTTCTAACGCTATCGGAAATAAGTGCCACGGATCGCCATTTCAACAGCCGGTATTGCGACTTTTCTCACTATATTCAAAATGCTGAGTACTATGAATGATCACCTCATCAGATATTTTTTTTAATGATTTTTAAGGTAAAAATGTTATTAAACAACTTTTTTTTGAAATAAATTACTAAACAACTTATAATATTATAACTTTTTCTCTATAACCTTTCGTAGAAGTACTTAGTATTGCATACCATAACCTTTTGAGACGTGATGCTTTTGACGATGTAGAATACTCTAAGCATAACCAGTTGTAATAAAGTAGTCGTTTGGTACTATTCTTTTAGATTTAGATCACTCTGAATTAATGTTCAAATCTTACAAAATTTTAACGAATGGGCACGGTTAATAAAAATTAGAAAGTTAGGGAGTACATATTTAAAATTAAGAGCACAAGGACTGGAAAAGAAGTTAATACAAACCTTAAGTGGGGTAACCTGAATTTATTTCAAATAAAAATTATATTAGTTTTAGAGAGTAATGTTAGGTGAACCAAATTTGGGGGAATCAGTTGCCGCAATACATGTCACTTAATGAAATTTTGTGATAAGTTGGACTTACTACCACATAAAGTGGGCAACAGGTGGTATAGTGCTAGGTGAATCACAATTTGAGGAACTACATGCAACCCACTTTATGTGACAGCTGATGTAGCAAAAACATATCAATTATGAAATTCTATAATAGGTTGGACTTGTTACCACATGATCAGTTGCCACATAAAATGCGATGTAAGTGGTATCCTAAAAGGTTGTTCACTTAGCATTAAAGCAGTTCTAACAGCTCCCCCATATATGAATGTTTCTCTATGTTAGAGAAAAATAAGCTTGTTTTGCTCTAAAAAATTCTCTATAACAATCCCTAACAGTGTGTTTGGATGAAGAAAAAATCAAGGGAATTTAAATGCAAATGAGGAATACCGCTGTGTTTGGATGAGACTTTTCTAAATTCTTGAGAAATTCTAAAATGATAGAATTTAAAAGTCCATTAATTAAAATCATTATATCTAAATTCTATTGTTTGCATGGTTGTAACTATCACGAATTTTGGAATGTCTAATGAATTTTGGAAAAATAAAATATTAGAAAAGAAAAAATAACTTGTTTTGGAAACTAATAAAGAGATATGAGGACACATTTCCGCACGATTTCCAAAATGACACCTATTTTGTTTTTGAAAGTAAAATCATATATATAAACGAAAGCCAGAATAACGATGTTACAATGACCACAAGAATCATAATAGTGTCACTATATTAGACAGTTAGCTCACTTATTGTGAGCTTAGCAGAGGATAAAACTAGTTACACACAAGATACTAGAGAGACATAAAACCGGAGTCAAGACGCTCAATTGCAATACTCCAAATCATATGAAACCATCTCGCTGCATCTAGCTCACACAGTCTGCCAAGCTTTCAAAGCACTAACACTAACTGAATTTTGGTGTTGCCTTTAGAGCAAATGCACCCATTTTAGGTGGGTTGGGCCAATTTTCAACCCAAGTTAATACATTATTCATTCAAATCAGTCTTTGGTCTTTGGTTCAACAATAGAACAACGAGATTAATCAAAGGACCGATCACTATTGACATATATAAACAATAGTTTTTTATTGACAAACATGTGTGGTCATGTGCAAAATGGTGTTATTGTGGCCCTCATTTATGGATTGGTCTGCGTTAATCCCTGGAGCGAGAATTACCCCATGCTATAGTCAGGTTAGGTGGGCTTCATATGGGACCCACGTCTAACTCAGCCCAATATCTCCTTGCTGGATTGCGTTTTTGGTCCTTTGACATGATCCACCCTCATTTTACCCCACTGCTGCATTTACTCTAAAAATCAGGGCCTGGAGGAGTGTTTGGGTAGAGCACCTTAGGAGCAGGGCTTAAAGGAAAGTCGTCGTCTGTTTTCCCATAAACTTTGTAGGTGTTAGCACCATGGGAGGGTAAGCCCATCCCATGATCAATTGAGGTCTAATATGGGTCATAGCCAGTCTCACCAAGCATGAGCCCAGACATCGGCCCAAAGATTTGGGTCTAGATATAGGTCCGAGCCTAGTGTTAAGAAACCGTGATCGACAGCCAAAGTAGCCATGTGTCGGCCTTCGATTTCCGATCAGCAACAGCGCCGCCTCACGCTTAAGACTTCTAGGAACTTGAACTAGGAAACTAGTCCAAAGTCGGGTAGTGCTAACCCGATTTGATTTCATCTCCACCAACGCGCTTAAAGCTTCTAGTATCAGAGATCCCCAAACCCTATTCCAATCGATAGGTTTCGTCATTGACTCTACAAGAGCCATCAATTTAAAGGCACTCTTTTCGGCTACTACTTTCCTTGATGTCCCAGACACCGGCCTAGTTTTCTTGAAATCATCTCCAACCACAGCAAAACACCATGGCTGGAGGCCTCGTTTGGTTGTGTTCGCCACGCCACTACTTGAGAGGATCACTCCTCGTTGAACCGCTACCGCTAAAAACCAACCACTTCATGGTCAGGCCGGGGGATCACTGGGTTCACTTGAAACCCTATTCCGCCATTTCAAAACCCTATGCCGCTCCCCAAAAGTCGGAGCATTCTCTCTTTTGGAAAAGGAAGAATAATGACAACTATTTTGTGGAAATTCAAGCGAGGAATTTAAACAATGAGTTCCTTTGTTTTCTTTTCCAAAGAAATTTAAAATCTCCTATTTGATAAGGTCAAACGGGGGAATTTTAGGAATTAGGAAATCCTCGCTTTCAATTTAACTTCGAGAAATTTTTCAGCATTACGGGAGGACCACATGACAATATGACGTGGTCCTACATTCCAATCAAATATTGACACTTGGATTTCACTAAAGAAAAATTACATCAGCTATTTTGTCAAAGACAATTTTGGGTTTGTTGTTGCTTTTTAAATCCTAAACTACAAACTCTAAACCCTAGACCTAAAACCCTAACCCCAAACCCCAAACCCCAAACCCTAAACCCTAAACCCTAGTCCCTAAACCCTAGTCCAAACTCAACAAAGACCCATGAAGGTCTTTTCATAAAAAATAGAAAACCTTAGTTTATATTTTAGTTGTAATAAATCTACATGTCAATATTTGATTGGAATATGTAGGACCACGTCATACTGTCACGTGGTTCTCTTGTAGCATCTAAAAATTTCTCTTTAACTGTATTGTTTTAATCCTCATCCAAACACATTGTACATGAATGACCTTGTTTGTCATTTGAAACTTTGCAATTATGAATTTCTTAGTGGACAAAAAAAAAATAACGTTGTGTTTGAATGCGGGGAAATTCCCTGGAATTTAATAGAAAGTAAGGATTTTATAATTTCTAAATGTCAATTCTCTTGTTTGGCATTATCAAATAGAGAATTTTAAATTTAGGTCATTTAAAATTCAGATGGAATTAAAAACAAAGGAATTCATTGATTAAATTCCTCACTTGAGTTCCACCAAAAATAGGTGTCATTTACAAATTACATTGCAGGATTCAATTTTCTTTTCCAGAACAAGTCTTTTTAAAATTTTATTTTTTATATTTCTTTCTAGTTTTCTTAATCTATCACAAACTTCAAATCCCAAATATACATAACCAAACAATGAAAATTGTAATTAACGGAATTTCAATTGATAGATATCTAAATTCCATCATTTCAGAATTCCTCAAGAATTAAGAAAACTCTCATTCAAACGCAACAAGTGAATTGATTGTCTAAATTCCTCACTTCAATTTCCATCAAGATATGTGTCATTTCGAAGTTTCTACTTCGATTCTTATCTCTTTATTAGTTTCCAAAACAATTTATATTTGTTTTTAAAAAATATTTATTTATTTTTTTTAATTTTCTCAATCCATTACACATTTCAAATTCTGGATGAATACAACCACACAAAAGAATTTGAATTTTACAGAATTTTAATTGACAGAGTTTCAAATTCCTCCATCATTTCACAATTTCTCAAGAATTTGAAAAGACTCATCCAAATATTGCGTAAGAGTGTATGGTTGAGAGAAAAAAAAATGTTGGAATTTAAATGAAAGTGAGGATTTCATAAATTTTAAAAGTCATTTTGGAATTACCGCACCCCAAATCCAAGTCGTGCAATGGTCCTCCGCCTCCCATCCCTTCTCCTTCCAAACCCAAGCCGCAATGGATTCGGGCTCCAATCACAGTGCTTGTCGCCGATGCTAAGCCGAATTTTCTCAGCCTCAAACTGTCCTCTAACAGCAAACAATAATCCTTTGTGGTTGATGAATTTTCAACCACGACTTCTTTGTACGAAGAAAATCCATCGAATTTCACAACCCACCTTTATACTTCGTTTAGCGGCCAACACTGATGGTGATTTTGTCACCGCCAAGTTTGTGGTAGTAGTATTTTTTCCGTTGGTGTTTTTTTCTTTGTGGTAGTAGCTTTCGTTGTCAGTGGTAGTATCTTTTGTTGTTTGTGGTAGTAGTTTCTGTTGCTGACAGTAGTATCTTTTGTTGCTGGTGGTAGTAGTTTTTGTTGCTCGTAGTAGTAGCTTTTGTTCATGGCGGTAGTATCTTTTGTTGTCAATGACAGTAGCTTTCTTTATTAGTGGTAGTAGCTTATGTTGTTGTCGATAGTATCTTTAATTGTCAGTTGTAGTACTTTTTGTGGTCACCAGAATGCTCATCGGAGGTCAGCTAGAGGTTGGCCGGAGACTTTTTCAAGGAGAAAAATGGTTGTTGACTTTTTACTCTAGTGATATTTTTGTAAATATAAGAGATATTCTAATTTTTCAGTTGGATGACAGTCTGTAATTTTGTTGAACTTTAATACCTAATATAAAACTCTATTTAGGCTTAAATAGACTTATGTGGGTAAAAATGTTTGAGTAGACCAATATAGAAAAAAAAAATGTCTCAAAATGGACTTATATGTAATTGGCCTTTTTTTTTTTTTTTATCCTTTTTCGTGACATAGTTCATCAAGCTTTTTTTTAGTTATAAAAAATGATATTTATATAATTTATTGATTTTTTCTTTTGTCAAGCATTTATTATGGGGCCTTAAAAAAATTTCCGCCTATAGCCTCGAAGCACACAGGGCCAACCCTATTGCTAGGGAGGATTCTAATTCCTCTATAAACTATATTTACCGAGAGAAATTTCCTTCCTTTCCGGAAAATTTGACTTGATTCTATTTAGATTTGGTTGTTACCTTTTAATCTCTTCTTCTAAGTTTAATAGAACCTGGAAATAAAGGTGCTATAAACCGTTATGAAAAGGTGGGTAAATTTTGGTTTGTAAGTCTAGACTAGGCTTGGTTTGTTAGCATAAAGAGGTCATTTTTTATTGTCGACCCTGATGGGTGGGTTATCTTAATATATACTGCGTGCTGCATAATATAATTGGTTGATTTTTGACTAAAAAAGAATTCATTGTTTAAATTCCTCACTTGAGAATTTTTACCAAAATAGTTGTCGTTTACAAGTTCCTTTGTATCATTCAATTTTCATTTCCAAACAATTTTTATATATATTATTTTTTATATTTATACTAAACTTTCTTAATCTATTACACACTTATAAACACACTAAAACCCGTTCTCTCCCCCAAAGCCTCCAAAAACCCTAAGCCTCCACTTCTTCCAAAACACTTGTCTGACGGCGACTGGATATGTGATGGCCTTTGGCCTCACTAGTCTTGGGTGTGTGCCGAACGGGTATCCTATTTTGATTTCAATCACGGAGTAAGTCTGATCTGAGCAAGGTTAAGGGGTTTCAAGCTTTCATGGTTTCTGAGCAAGTAGGCGTCGGCACAAGCAAGGGTGACTTGGCTAGATCTCATAGCCGACATCAATGGTGAGATCCATTTTTTTTGGCATAGGTTTTTGGGGTGATTTTTGTTTGGAGCTTGCCAACTTCCCACTACCGTTGAATCGGGGATCTATGTTGGTGCGTGCTGCTGAGGGAGGAGTCTCATGGTTGTGGGGTTTGAAAGTGGTGGGTTGTGATGTGACTGGGTCGGTGACGGAGTTCTAGCTGTGGTTGGGATCCGACAAGGCGACAACGGCAGAGGTGGCATGACAGTGTTGCTGGTGGTGAGATGGTTGCTAGTGGTGATTGCAGACTTGGGCCGAGATCATGGGCCGGACCTATTTGACCTTGCTTCACTGGTTTGCTTTGGTCCTAATGTCTTGGGCCTAGGGTTTTTACTCTGGGTCCAAGATGGTTATCTCTAGTTTTCTAAGCCTTTTTTGGGCCTCTAAGGTTTTAAATTAGTCTATTTTGCTAGTTTATTTCAAATAATTTTACTATGTAGGTTTCTAGGAACTTTAGCTAGATAAGTTTTATGTCTACAGATTATGATGTTTCTTTTACATTTAGATAATAGACACTTCTTATCGTACCACAATTGAGCGCCTTGGCGACGAAAGTTTTCATACAAAATTATCTCTATGTAGAGCTAGATTATAGCTTGCTACGCTCATCGTTGATGAGTGACAATGTACAATGTAATGATATATGAATCACTCAATTCATTAGGATCTGTATCGGTGTCATTCTGGCTGCGATTACTAATACAAAAAGTTACATTCCCGCCTTTGCTTCAAAAAAAATAAAAAATCTACTATACACTTCAAATCCCAAATACACACAACCAAACAATAAAAATTGCAAAGATGAAAAACCCAAGCTCTCTCTCTCTCTCTCTCTCTCTCTCTCTCTCTCTCTCTCAGCTATGGGTGGTAGAGCGCCGCCACCTCCGGCCTCGGAACTCCTCTTCACTCTGCGGTGCCTCGGCCTCGGAACTCCTCTGCCTTCTGTAATTAAGGGAATATAACAAAATGAGGTTGGTTTGGCCTGAAAGTCTGGGTTGATTTTCAGCAAACGTTATTTACAAGGTCTGGGTTGATTTTCTTTTGTTTTGTGTCTTTCACTTTTGGCAAGTTTTCAGTCTGAACCCGACCATCTTTCCTTGTTTTTGTTCCATATGAACATCATCCCAGGCAATTAACAAAGTCTTTATGCTTAGATTTTAGATCGAGCTATAATATTTATCGAACTGATTGTTGGTTATGTAGAGATTGATCACCAACAAAATCTCTAAAAAAAATTTGAAGAAATAAGGAGATTTTTTTATTGTAAAATAGAGAATATGTTGAGTTGAAGTATGGCGATTAGTAAAAATTTAACTTTTTGCTTCATAAACATGTTGGAATTGCTCTTTCAGTTTTAAATGGAGATTTATTTTTTCGACAATTTCCCAATGAAGTTTTTGATCCAAACTTCTGCATGTAACGAAACCAATTGGATAATATTGATTAACTTTTTTCAAAGGATTTAATATTTTATTTGATTTTTATGAGGTGTTGAACTCCGAAAGAGGGTGGTAAGAATAAGTCAGCATAAATTCCAACTCTTTAATTTTTTTTTTTTTCTAAATGCTTTCAATATTTGAATGCCCAGGCCCCTCCATTGGAAGCCAATTTGAAGATCTCTTGGCCCAACCTAGAAAATACGAGTGATTTGCAGTTTTGGGAACGTCAGTGGGACAGACATGGCATGTGTTCAGAGCCGACGTTTACGCAAACGCAGTACTTCACACGAGCGCATGAAATTTGGATGACGGATGACATCAATGTTACTGATATCCTCCGAAAAGTTAACGTCTTATCAGGGACACAAAAAGAGTACGCAGAGATAGAATACCCGATTGAATTAAAAATTCAAAAGACACCTCTTCTTCGCTGCCTAAACCAAAAAAACTCTCAGTCGCACTCTCAGATGTTGCATGAAGTGGTAATTTGCTGGGACCATAAGGCTAAAAAGATGACGGACTGTAATGCTGCGGAAGCAACATGCTCAAGGAAGAGTCCAATTGATATTCTATAGAGTTTCTCAAAAATAGGAAAGTTACTCTGTAATCACTAGCCTCTGCCCTCTCGACACGAAAATTCGTATCATTTGATATTTTAGAGAAATATAAAACAGTAACTAAATAATGAATTACATCAAAAATAAAAAATAAAACAAATTATAAAAATATGTTTTTCCTTCCTCTTTTTTTTTGAAAGAAAAATTTGTCTTTCCTTATATTTTCTATGAGATTATTGACAATATTCTTCTTAATGTTTTTCTTTTTTGATAGAAGGGCCTTTACGGCCAATTTCATTAATTAGAAACCAGAATGACACGAATACATGTCTTCTCTTGTCAAACGTTTGGGCAAGTTAAAGACTTGGCATGCTAGCACCATTCATTACAAATTTCGCTCACTTAATTCAATACAGAAAAGAAAAATTTGAACCTTAATTAGACCATGGAACAAACCACAACAATTAGACACACACACAAAGAGCTTAGACTCCTAAAAAAGGAATTATTGTAAATAGACTAGCTACATACTTAAAAATGGGCCAAAGTAAAC
